# Revision of the genus *Pseudapanteles* (Hymenoptera, Braconidae, Microgastrinae), with emphasis on the species in Area de Conservación Guanacaste, northwestern Costa Rica

**DOI:** 10.3897/zookeys.446.8195

**Published:** 2014-10-14

**Authors:** Jose L. Fernández-Triana, Daniel H. Janzen, Winnie Hallwachs, James B. Whitfield, M. Alex Smith, Robert Kula

**Affiliations:** 1 Canadian National Collection of Insects, 960 Carling Ave., Ottawa, ON K1A 0C6 Canada; 2 Biodiversity Institute of Ontario, University of Guelph, Guelph, ON N1G 2W1 Canada; 3 Department of Biology, University of Pennsylvania, Philadelphia, PA 19104-6018 USA; 4 Department of Entomology, University of Illinois, Urbana, IL 61801 USA; 5 Department of Integrative Biology, University of Guelph, Guelph, ON N1G 2W1 Canada; 6 Systematic Entomology Laboratory, Beltsville Agricultural Research Center, Agricultural Research Service, U.S. Department of Agriculture, c/o National Museum of Natural History, Smithsonian Institution, P.O. Box 37012, MRC-168, Washington, DC 20013-7012, USA

**Keywords:** *Pseudapanteles*, Microgastrinae, New World, Area de Conservación Guanacaste, taxonomic review, parasitoid wasps, caterpillars, DNA barcoding, host species, Lucid software

## Abstract

*Pseudapanteles* is a moderately diverse genus of Microgastrinae parasitoid wasps (Hymenoptera: Braconidae), endemic to the New World and with the vast majority of its species (including many undescribed) in the Neotropical region. We describe here 25 new species from Area de Conservación Guanacaste (ACG), northwestern Costa Rica, based on 400 studied specimens. A key to all 36 known species of *Pseudapanteles* is provided (except for *Pseudapanteles brunneus*, only known from a single male), and species are placed in three newly created species-groups. Host records are known for only 25% of the species; most are solitary parasitoids of the caterpillars of several families of small Lepidoptera (Crambidae, Elachistidae, Gelechiidae, Incurvariidae, Sesiidae, Tineidae). DNA barcodes (part of the CO1 gene) were obtained for 30 species (83%), and provide a start for future study of the genus beyond ACG. Brief descriptions (generated by Lucid 3.5 software) and extensive illustrations are provided for all species. The following new taxonomic and nomenclatural acts are proposed: *Pseudapanteles moerens* (Nixon, 1965), **comb. n.**, *Pseudapanteles brunneus* Ashmead, 1900, **comb. rev.**, a lectotype is designated for *Pseudapanteles ruficollis* (Cameron, 1911), and the following 25 species nova of *Pseudapanteles* (all authored by Fernández-Triana and Whitfield): *alfiopivai*, *alvaroumanai*, *analorenaguevarae*, *carlosespinachi*, *carlosrodriguezi*, *christianafigueresae*, *hernanbravoi*, *jorgerodriguezi*, *josefigueresi*, *laurachinchillae*, *luisguillermosolisi*, *margaritapenonae*, *mariobozai*, *mariocarvajali*, *maureenballesteroae*, *munifigueresae*, *oscarariasi*, *ottonsolisi*, *pedroleoni*, *raulsolorzanoi*, *renecastroi*, *rodrigogamezi*, *rosemarykarpinskiae*, *soniapicadoae*, *teofilodelatorrei*.

## Introduction

Area de Conservación Guanacaste (ACG), in northwestern Costa Rica, has been inventorying all caterpillar taxa, their food plants, and their parasitoids since 1978 (Janzen et al. 2009, 2012, Fernández-Triana et al. 2014). The resulting thousands of specimens available for study provide the world’s best tropical location-based dataset for studying the taxonomy and host relationships of caterpillar parasitoids.

For the past decade, Microgastrinae wasps (Hymenoptera: Braconidae) have been one of the most frequently reared and intensively studied groups of parasitoids in ACG (references to previously published papers were summarized in Fernández-Triana et al. 2014), although hundreds of species remain undescribed.

This paper is a continuation of those studies. It revises *Pseudapanteles*, a moderately diverse genus of Microgastrinae, which includes nine described species but many additional undescribed species in collections, mostly from South America. Ashmead (1898) originally described the genus, but it was not until Mason (1981) reclassified Microgastrinae that *Pseudapanteles* became widely accepted as a distinct genus. Mason redefined *Pseudapanteles* and restricted it to contain only eight New World species (six in the Neotropics), while also acknowledging that many undescribed Neotropical species were present in collections (Mason 1981). The limits of the genus (at least based on diagnostic morphological characters) have not changed, and scarce progress has been made since Mason’s work, with only one new species from the Nearctic, *Pseudapanteles gouleti* (Fernández-Triana 2010), being recently described.

*Pseudapanteles* species are mostly solitary parasitoids of caterpillars of several families of small Lepidoptera (Crambidae, Elachistidae, Gelechiidae, Incurvariidae, Sesiidae, Tineidae). However, most of the wasp species remain without known lepidopteran hosts.

We describe here 25 new species from ACG and provide a key and illustrations for all previously described species.

## Methods

**Figure 1.**
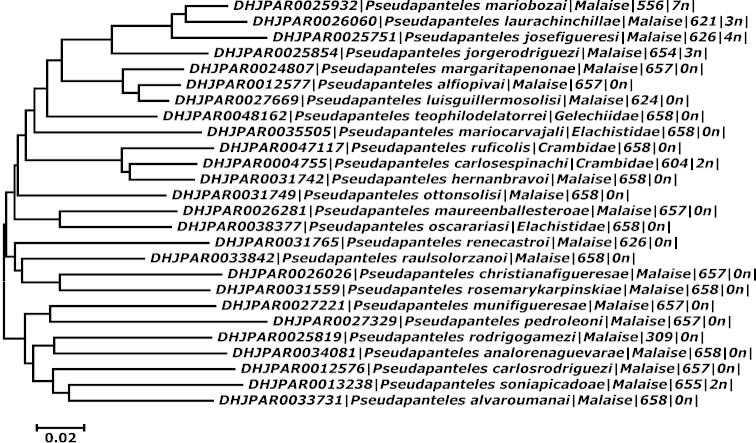
Neighbor-Joining (NJ – Saitou and Nei 1987) tree based on Kimura 2-parameter distances (K2P – Kimura 1980) made using MEGA6 (Tamura et al. 2013) of a single representative sequence from each species, selected based on longer read length and lower number of ambiguities. An NJ tree of all ACG specimens, made using BOLD, can be seen in Suppl. material 1.

This study is based on wasp specimens from ACG that were either reared from caterpillar hosts or collected using Malaise traps; their host caterpillars and other ecological information are considered along with the 658 bp DNA barcode region of the cytochrome *c* oxidase I (COI) gene (Hebert et al. 2003) when available (Fig. 1). We also studied the holotypes of all 10 previously described species of *Pseudapanteles*. They are deposited in the Canadian National Collection of Insects, Ottawa, Canada (CNC), the Natural History Museum, London, England (BMNH), and the National Museum of Natural History, Smithsonian Institution, Washington DC, United States (NMNH).

Specimens of the new species are deposited in the CNC, BMNH, NMNH, the Illinois Natural History Survey, Champaign, United States (INHS), and the Instituto Nacional de Biodiversidad, Santo Domingo, Costa Rica (INBio).

Morphological terms and measurements of structures are mostly as used by Mason (1981), Huber and Sharkey (1993), Whitfield (1997), Karlsson and Ronquist (2012), and Fernández-Triana et al. (2014). Natural history information (e.g., geographical distribution, hosts) is also provided in the key when available for a species. Those data are included in brackets at the end of the corresponding couplet and are intended as supplementary information to aid identification.

Descriptions of the new species are based on the study of all female specimens that were available for study (to reflect intraspecific variation), but always include data from the holotype. As an exception, three new species were described from males only because they were distinct enough to be recognized; the males of those three species will run through the key, but males of most species may not be readily identified unless associated with females via rearing or molecular data.

Lucid 3.5.4 (http://www.lucidcentral.com/) software was used to generate automatic descriptions of the species and to prepare Lucid identification keys. A dataset of 15 characters and 95 character-states was used to provide uniform descriptions for all new species. The description format includes one phrase per character, with the character mentioned first and the character-state following after a colon, e.g., “Metatarsus color: pale”. Whenever a species scored more than one character-state, the description included all of the pertaining character-states separated by “or”, e.g., “Metatarsus color: pale or dark”. Whenever a character-state was coded as uncertain due to poor condition of a specimen, the description includes the details of the character-state as best assessed, followed by a question mark, e.g., “Metatarsus color: pale (?)”. Sometimes a character could not be coded due to missing body parts in the available specimens; in such instances the feature was left out of the description for that particular species.

In most cases we used a simplified convention to code color, considering it as either pale (light yellow, orange-yellow, light brown-yellow) or dark (dark brown, black). For details on the exact color patterns on the body, we provide extensive photographic illustrations for every species.

We had to use logical characters in some couplets of the dichotomous key (e.g., “if”, “then”, “and”, “or”, “and/or”). Those words are shown in bold and italic to be explicit that in those cases more than one character state has to be considered.

Most photos were taken with a Keyence VHX-1000 Digital Microscope, using a lens with a range of 13–130 ×. Multiple images through the focal plane were taken of a structure and these were combined to produce a single in-focus image, using the software associated with the Keyence System.

Images of holotypes deposited in the NMNH were obtained using a GT Vision EntoVision imaging system consisting of a firewire JVC KY-75 3CCD digital camera mounted on a Leica M16 zoom lens via a Leica z-step microscope stand. The camera fed a desktop computer where the Archimed software program was used to export image stacks, and the CZPBatch software program was used to generate a composite image from the exported image stacks. Composite images were edited using Adode Photoshop CS4 to remove artifacts from stack processing and standardize background color. Plates were assembled using Adobe Illustrator CS4.

A map with the distribution of all species was generated using SimpleMappr (Shorthouse 2010).

DNA barcodes for all ACG inventory *Pseudapanteles* were obtained using DNA extracts prepared from single legs using a glass fibre protocol (Ivanova et al. 2006). Briefly, total genomic DNA was re-suspended in 30 μl of dH2O, and a 658-bp region near the 5’ terminus of the COI gene was amplified using standard primers (LepF1–LepR1) following established protocols (Smith et al. 2006, 2007, 2008). If the initial 658 bp amplification was unsuccessful, composite sequences were generated using internal primers. All information for the sequences associated with each individual specimen can be retrieved from the Barcode of Life Data System (BOLD) (Ratnasingham and Hebert 2007) by Process ID (sequence accession) or here: http://dx.doi.org/10.5883/DS-ASPSE.

In the taxonomic treatment of species, full details of the collecting (type) locality are given only for the holotype. We provide the country and province for paratypes, followed by ACG database codes (in the format “yy-SRNP-xxxxxx” for the host caterpillar or parasitoid lot reared from it, or “DHJPARxxxxxxx” for an individual parasitoid specimen). Those codes allow for the retrieval of detailed information of any specimen at http://janzen.sas.upenn.edu.

The new species from ACG were named to honor many of the Costa Rican political figures who have been important in all aspects of the founding, growth and evolution of ACG.

## Results and discusion

**Figure 2.**
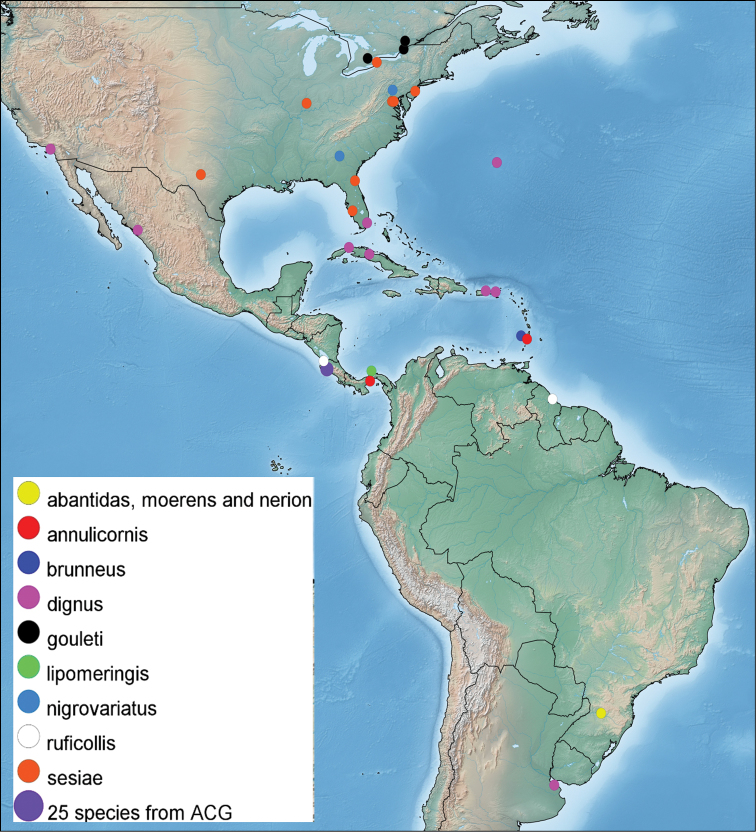
Distribution map of the described species of *Pseudapanteles* in the New World.

*Pseudapanteles* is widely distributed in the New World (Fig. 2), ranging from 34°S in Argentina to 45°N in Canada (Whitfield 1995, 1997, Fernández-Triana 2010, 2014, Yu et al. 2012; data herein). Most of the species are Neotropical, with just a few extending north into the Nearctic Region. The collections we have examined contain many tens of additional undescribed species, mostly from South America (this area is understudied yet is probably the richest). We estimate that the actual richness of the genus will easily surpass 100 species when intensive studies, such as the present one in ACG, are done elsewhere.

Mason (1981) characterized the hosts for species of *Pseudapanteles* as being plant-boring microlepidopterans, and subsequent rearing, mostly done in ACG, supports this assertion. However, there are also some leafminer hosts which appear to be restricted to only a few of the *Pseudapanteles* species found so far.

The genus *Pseudapanteles* is characterized by its elongate glossa which is strongly bilobed apically (as in Figs 40, 52, 95, 139, 144, 149, 152, 175), propodeum with a strongly defined median longitudinal carina (as in Figs 13, 18, 34, 44, 69, 77, 88, 93, 97, 101, 125, 140, 154, 177) but no transverse carina (traces of a transverse carina are very rarely present in a few Neotropical species), mediotergite 1 with a sharp median sulcus (as in Figs 13, 17, 46, 73, 88, 93, 101, 120, 125, 138, 182, 185), hypopygium with a large translucent fold with many pleats (as in Figs 6, 15, 30, 42, 57, 72, 128, 133, 141, 168, 171), and ovipositor sheaths at least 0.7 × as long as metatibia length. The only other genus that could be confused with *Pseudapanteles* is the more recently described *Mariapanteles* due to similar morphological features. However, *Mariapanteles* differs in having a complete or almost complete transverse carina on the propodeum which forks around the spiracles and reaches the lateral margins of the propodeum, and the hypopygium with no or few pleats (Whitfield et al. 2012). Another feature discussed by Whitfield et al. (2012) as being diagnostic to separate the two genera, the elongate glossa, is present in species of both genera and thus is no longer useful.

Below we describe 25 new species from ACG and propose two new combinations: *Pseudapanteles moerens* (Nixon, 1965), comb. n. (transferred from *Apanteles*), and *Pseudapanteles brunneus* Ashmead, 1900, comb. rev. (transferred from *Apanteles*). We recognize 36 species of *Pseudapanteles* as a result of this research (Table 1).

**Table 1.** Species of *Pseudapanteles* currently recognized and their known distribution. All known records for Costa Rica are from Area de Conservación Guanacaste. The (*) after Hawaii means that *Pseudapanteles dignus* is not a native species but was introduced there.

Species	Species-group	Known distribution
*Pseudapanteles abantidas* (Nixon, 1965)	*gouleti*	Brazil
*Pseudapanteles alfiopivai* Fernández-Triana & Whitfield, sp. n.	*annulicornis*	Costa Rica
*Pseudapanteles alvaroumanai* Fernández-Triana & Whitfield, sp. n.	*annulicornis*	Costa Rica
*Pseudapanteles analorenaguevarae* Fernández-Triana & Whitfield, sp. n.	*annulicornis*	Costa Rica
*Pseudapanteles annulicornis* Ashmead, 1900	*annulicornis*	Panama, St. Vincent
*Pseudapanteles brunneus* Ashmead, 1900, comb. rev.	*annulicornis*	St. Vincent
*Pseudapanteles carlosespinachi* Fernández-Triana & Whitfield, sp. n.	*annulicornis*	Costa Rica
*Pseudapanteles carlosrodriguezi* Fernández-Triana & Whitfield, sp. n.	*gouleti*	Costa Rica
*Pseudapanteles christianafigueresae* Fernández-Triana & Whitfield, sp. n.	*annulicornis*	Costa Rica
*Pseudapanteles dignus* (Muesebeck, 1938)	*annulicornis*	Argentina, Bermuda, Cuba, Hawaii (*), Mexico, Puerto Rico, United States, Virgin Islands
*Pseudapanteles gouleti* Fernández-Triana, 2010	*gouleti*	Canada
*Pseudapanteles hernanbravoi* Fernández-Triana & Whitfield, sp. n.	*annulicornis*	Costa Rica
*Pseudapanteles jorgerodriguezi* Fernández-Triana & Whitfield, sp. n.	*annulicornis*	Costa Rica
*Pseudapanteles josefigueresi* Fernández-Triana & Whitfield, sp. n.	*annulicornis*	Costa Rica
*Pseudapanteles laurachinchillae* Fernández-Triana & Whitfield, sp. n.	*annulicornis*	Costa Rica
*Pseudapanteles lipomeringis* (Muesebeck, 1958)	*annulicornis*	Panama
*Pseudapanteles luisguillermosolisi* Fernández-Triana & Whitfield, sp. n.	*annulicornis*	Costa Rica
*Pseudapanteles margaritapenonae* Fernández-Triana & Whitfield, sp. n.	*annulicornis*	Costa Rica
*Pseudapanteles mariobozai* Fernández-Triana & Whitfield, sp. n.	*annulicornis*	Costa Rica
*Pseudapanteles mariocarvajali* Fernández-Triana & Whitfield, sp. n.	*mariocarvajali*	Costa Rica
*Pseudapanteles maureenballesteroae* Fernández-Triana & Whitfield, sp. n.	*gouleti*	Costa Rica
*Pseudapanteles moerens* (Nixon, 1965), comb. n.	*annulicornis*	Brazil
*Pseudapanteles munifigueresae* Fernández-Triana & Whitfield, sp. n.	*annulicornis*	Costa Rica
*Pseudapanteles nerion* (Nixon, 1965)	*annulicornis*	Brazil
*Pseudapanteles nigrovariatus* (Muesebeck, 1921)	*annulicornis*	United States
*Pseudapanteles oscarariasi* Fernández-Triana & Whitfield, sp. n.	*gouleti*	Costa Rica
*Pseudapanteles ottonsolisi* Fernández-Triana & Whitfield, sp. n.	*annulicornis*	Costa Rica
*Pseudapanteles pedroleoni* Fernández-Triana & Whitfield, sp. n.	*annulicornis*	Costa Rica
*Pseudapanteles raulsolorzanoi* Fernández-Triana & Whitfield, sp. n.	*gouleti*	Costa Rica
*Pseudapanteles renecastroi* Fernández-Triana & Whitfield, sp. n.	*annulicornis*	Costa Rica
*Pseudapanteles rodrigogamezi* Fernández-Triana & Whitfield, sp. n.	*annulicornis*	Costa Rica
*Pseudapanteles rosemarykarpinskiae* Fernández-Triana & Whitfield, sp. n.	*gouleti*	Costa Rica
*Pseudapanteles ruficollis* (Cameron, 1911)	*annulicornis*	Costa Rica, Cuba, Guyana
*Pseudapanteles sesiae* (Viereck, 1912)	*annulicornis*	Canada, United States
*Pseudapanteles soniapicadoae* Fernández-Triana & Whitfield, sp. n.	*gouleti*	Costa Rica
*Pseudapanteles teofilodelatorrei* Fernández-Triana & Whitfield, sp. n.	*annulicornis*	Costa Rica

The new species described from ACG more than double the previous number of known species of *Pseudapanteles*. We are aware of an additional four to six species in the ACG inventory which are only represented by males and are virtually impossible to fully distinguish morphologically; they will remain undescribed until female specimens are available.

The known species of *Pseudapanteles* can be placed in three morphologically distinctive species groups, all of which are newly proposed in this paper. The *mariocarvajali* species-group comprises one species from ACG, which is unique on the basis of the almost quadrate mediotergite 2, and also has a large body size and fore wing length (>3.4 mm). Known hosts include species in two genera of Elachistidae, *Chlamydastis* and *Stenoma*. We have not seen more species of this group in the collections studied.

The *gouleti* species-group comprises eight described species, and a few other undescribed ones seen in collections. It includes two subgroups, one with the propodeum strongly sculptured (with transverse striation that sometimes look like carinae), and another subgroup with the propodeum clearly differentiated into an elevated central area and depressed posterolateral corners (with variable sculpturing). Known hosts are species of Elachistidae and Incurvariidae. The species *rosemarykarpinskiae* shows some features, especially the forewing venation and sculpturing of T1 and T2, that resemble the genus *Rhygoplitis* – a not particularly close genus based on previous studies, and thus is likely to be another example of convergence within Microgastrinae, as it has been pointed out in the past (e.g., Mason 1981). The *gouleti* species-group is likely to be split when the Neotropical fauna (especially that of South America) is further studied; some of its component species might even be placed in *Mariapanteles*. Conversely, the genus *Mariapanteles* might ultimately be synonymized with *Pseudapanteles* (e.g., Whitfield et al. 2012). Lacking a more complete and robust phylogeny of Microgastrinae, we tentatively consider this a species-group of *Pseudapanteles*.

The *annulicornis* species-group comprises the majority of the known species (27), as well as many tens of undescribed species seen in collections. It includes species of *Pseudapanteles* with a rather smooth propodeum and a strong median carina, the most commonly encountered propodeal sculpture condition in the genus; body size varies from the smallest known specimens of *Pseudapanteles* (~1.6 mm) to specimens up to 3.0 mm long (excluding the ovipositor). Known hosts are species of Crambidae, Gelechiidae, Sesiidae, and Tineidae. This group may also be split into several in the future, after the Neotropical fauna has been studied in detail.

### Key to species of *Pseudapanteles*

[Below we use “T” to refer to mediotergites (e.g., T1 = mediotergite 1). The key is intended for female specimens, although males of some species will run to the correct couplet. The species *Pseudapanteles brunneus* Ashmead, 1900, described from St. Vincent Island (Caribbean), is only known from the male holotype, and cannot be identified using this key; however, there are only two known species of *Pseudapanteles* from St. Vincent Island: *Pseudapanteles brunneus*, which is dark brown on most of the meso- and metasoma dorsally, and *Pseudapanteles annulicornis*, which is entirely orange-yellow].

**Table:** 

1	T2 subquadrate, width at posterior margin 1.7–1.8 × its length (Figs 100, 101); body length and fore wing length at least 3.4 mm (*mariocarvajali* species-group) [Hosts: *Chlamydastis* and *Stenoma*, Elachistidae. Distribution: ACG]	***Pseudapanteles mariocarvajali* Fernández-Triana & Whitfield, sp. n.**
–	T2 more transverse, width at posterior margin at least 2.7 × its length, usually much more (Figs 5, 13, 17, 26, 34, 41, 44, 49, 58, 64, 67, 73, 78, 80, 88, 91, 98, 112, 120, 125, 127, 135, 140, 148, 155, 161, 165, 167, 170, 179, 185); body length and fore wing at most 3.0 mm, usually much less (***if*** rarely T2 width at posterior margin 2.1 × its length, then body and fore wing length 1.6 mm)	**2**
2(1)	Propodeum mostly smooth and shiny, with well-defined median carina (at most with very few, short rugosities transverse to median carina) (as in Figs 13, 18, 34, 44, 49, 64, 69, 77, 88, 93, 97, 98, 109, 125, 145, 154, 159, 167, 185); ***and*** propodeum not differentiated into elevated central area and depressed posterolateral corners (*annulicornis* species-group)	**3**
–	Propodeum dull and mostly sculptured, covered by numerous transverse rugosities in addition to well-defined median carina (as in Figs 58, 135, 148, 150, 164, 165); ***if*** propodeum less sculptured, ***then*** clearly differentiated into elevated central area and depressed posterolateral corners (as in Fig. 177) (*gouleti* species-group)	**28**
3(2)	Mesosoma entirely or almost entirely dark brown (at most orange-yellow on propleuron, pronotum partially, small spot on upper corner of mesopleuron, and small marks centrally on anteromesoscutum) (as in Figs 14, 19, 23, 34–36, 38–40, 44–46, 48–50, 77, 78)	**4**
–	Mesosoma with extensive orange to orange-yellow coloration (as in Figs 7–12, 24, 25, 59–62, 79–82, 84–88)	**17**
4(3)	All coxae entirely yellow or orange-yellow (at most with very small brown spot dorsally on anterior 0.1 of metacoxa) (Figs 49, 50, 74, 78, 121–125)	**5**
–	Metacoxa entirely or partially brown (with at least brown spot covering anterior 0.3 of coxa) (as in Figs 46, 64, 65, 71, 72, 94, 105, 116, 154, 155)	**7**
5(4)	Anterior 0.6 of mediotergite 1 and most of laterotergites orange-yellow (Fig. 49) ***and*** pterostigma transparent with thin margins brown (Fig. 51); ovipositor sheaths 0.7 × as long as metatibia; mediotergite 1 less strongly narrowing towards posterior margin (maximum width less than 2.0 × tergite width at posterior margin) (Fig. 49) [Hosts: six genera of Gelechiidae (*Keiferia*, *Phthorimaea*, *Symmetrischema*, *Tildenia*, *Tuta*). Distribution: Argentina, Bermuda, Cuba, Mexico, Puerto Rico, United States, Virgin Islands; introduced to Hawaii]	***Pseudapanteles dignus* (Muesebeck, 1938)**
–	Metasoma dorsally entirely brown or mostly brown (Figs 75, 78, 121–125) ***and/or*** pterostigma entirely brown (Fig. 75); ovipositor sheaths at least 0.9 × as long as metatibia (Fig. 121, 122); mediotergite 1 strongly narrowing towards posterior margin (maximum width more than 2.0 × tergite width at posterior margin) (Figs 78, 122, 125)	6
6(5)	Metasoma almost entirely dark brown (except laterotergites 1 and 2) (Figs 121–125); T2 width at posterior margin 2.9 × its length (Fig. 125); ovipositor sheaths as long as metatibia (Figs 121, 122) [Distribution: Brazil]	***Pseudapanteles nerion* Nixon, 1965**
–	Metasoma yellow-orange on anterior 0.5–0.6 of T1 and most of laterotergites and hypopygium (Fig. 74); T2 width at posterior margin 3.6–3.7 × its length (Fig. 78); ovipositor sheaths slightly shorter (0.9 x) than metatibia (Fig. 74) [Distribution: Costa Rica, ACG]	***Pseudapanteles laurachinchillae* Fernández-Triana & Whitfield, sp. n.**
7(4)	Head, flagellomeres, mesosoma and metasoma mostly dark brown to black (Figs 32, 34–36, 169–173); body length and fore wing length usually 2.3–3.0 mm	**8**
–	Head, mesosoma, and/or metasoma with yellow-orange coloration in some areas (as in Figs 14, 16, 22, 23, 45), ***if*** mostly dark brown, then body length and fore wing length less than 1.8 mm; body length usually less than 2.2 mm, ***if*** 2.5–2.8 mm ***then*** antenna with central flagellomeres white	**9**
8(7)	Body length and fore wing length usually 3.0 mm; T1 relatively slightly narrowing towards posterior margin, its maximum width at most 1.4 × its width at posterior margin (Fig. 170); T2 width at posterior margin 2.5 × its length (Fig. 170) [Host: *Synanthedon scitula*, Sesiidae. Nearctic species. Distribution: Canada (Ontario) and the United States (District of Columbia, Florida, Indiana, New Jersey, Texas, Virginia)]	***Pseudapanteles sesiae* (Viereck, 1912)**
–	Body length and fore wing length 2.3–2.5 mm; T1 relatively strongly narrowing towards posterior margin, its maximum width 2.9 × its width at posterior margin (Figs 34–36); T2 width at posterior margin 3.2 × its length (Fig. 34) [Host: *Desmia* sp., Crambidae. Neotropical species. Distribution: Costa Rica, ACG]	***Pseudapanteles carlosespinachi* Fernández-Triana & Whitfield, sp. n.**
9(7)	Antenna with central flagellomeres white and remaining flagellomeres brown (Figs 19, 22, 42); body length 2.5–2.8 mm, fore wing length 2.6–2.9 mm	**10**
–	Antenna with all flagellomeres dark brown; body length 1.6–2.2 mm, fore wing length 1.6–2.2 mm	**11**
10(9)	Head mostly brown-black posteriorly, but orange on most of frons and face (Fig. 45); flagellomeres 6–10 (and posterior half of flagellomere 5) white (Fig. 42); anteromesoscutum entirely brown to black (Figs 44, 46); metatibia and metatarsus entirely yellow to orange (Figs 42, 46, 47)	***Pseudapanteles christianafigueresae* Fernández-Triana & Whitfield, sp. n.**
–	Head posteriorly, frons and face brown-black (Fig. 19); flagellomeres 7–9 white (Figs 19, 22); anteromesoscutum with orange marks centrally (Figs 22, 23); metatarsus entirely, and posterior 0.2 of metatibia brown (Fig. 22)	***Pseudapanteles analorenaguevarae* Fernández-Triana & Whitfield, sp. n.**
11 (9)	Propleuron, pronotum partially, small spot on upper corner of mesopleuron, and small marks centrally on anteromesoscutum orange-yellow, rest of mesosoma dark brown (Figs 14, 16); head posteriorly dark brown to black, but partially orange on frons and face (Fig. 16)	***Pseudapanteles alvaroumanai* Fernández-Triana & Whitfield, sp. n.**
–	Mesosoma entirely dark brown; head posteriorly, frons and face brown to black (Figs 95–98, 116, 117, 119, 120, 156, 157, 159–161)	**12**
12(11)	Metatibia dark brown at least on posterior 0.6 (as in Figs 116, 156, 158, 159); T1 maximum width (at approximately half length of tergite) at least 2.9 × its width at posterior margin (as in Figs 120, 161); T2 width at posterior margin at most 2.8 × its length	**13**
–	Metatibia dark brown at most on posterior 0.2 (Figs 65, 151, partially seen in Figs 71, 72); T1 maximum width (at approximately half length of tergite) 2.2–2.5 × its width at posterior margin; T2 width at posterior margin 3.7–4.1 × its length	**15**
13(12)	Metatibia dark brown on posterior 0.9 (partially seen in Figs 156, 158, 159); pterostigma yellow-white, with very thin brown margins (Fig. 158); T1 length 4.6 × its width at posterior margin (Fig. 161); body length and fore wing length 1.6 mm	***Pseudapanteles rodrigogamezi* Fernández-Triana & Whitfield, sp. n.**
–	Metatibia dark brown on posterior 0.6 (partially seen in Figs 94, 116); pterostigma entirely brown (Fig. 96); T1 length at least 5.5 × its width at posterior margin; body length and fore wing length at least 1.8 mm	**14**
14(13)	T1 maximum width (at approximately half length of tergite) 3.8 × its width at posterior margin; T2 width at posterior margin 2.8 × its length (Fig. 120); body length and fore wing length 1.8 mm	***Pseudapanteles munifigueresae* Fernández-Triana & Whitfield, sp. n.**
–	T1 maximum width (at approximately half length of tergite) 2.8 × its width at posterior margin; T2 width at posterior margin 2.2 × its length (Figs 96–98); body length and fore wing length at least 2.1 mm	***Pseudapanteles mariobozai* Fernández-Triana & Whitfield, sp. n.**
15(12)	Ovipositor sheaths 0.7 × as long as metatibia (Fig. 71); T1 length 4.0 × its width at posterior margin (Fig. 73)	***Pseudapanteles josefigueresi* Fernández-Triana & Whitfield, sp. n.**
–	Ovipositor sheaths 0.9–1.0 × as long as metatibia (Figs 65, 151, 155); T1 length at least 4.5 × its width at posterior margin	**16**
16(15)	T2 mostly longitudinally striate (except for small smooth central area) (Fig. 67); propodeum mostly smooth, with only median longitudinal carina (Fig. 66, 69); scutoscutellar sulcus with 6 impressed pits; ovipositor sheaths 0.9 × as long as metatibia (Fig. 65) [Rain forest, 575 m]	***Pseudapanteles jorgerodriguezi* Fernández-Triana & Whitfield, sp. n.**
–	T2 mostly smooth and polished (Figs 154, 155); propodeum with short, carina like sculpture on lateral and posterior margins in addition to median longitudinal carina (Fig. 154); scutoscutellar sulcus with at least 8 impressed pits; ovipositor sheaths 1.0 × as long as metatibia (Figs 151, 155) [Dry forest under 300m]	***Pseudapanteles renecastroi* Fernández-Triana & Whitfield, sp. n.**
17(3)	Head entirely yellow-orange (Figs 79, 81, 83, 110, 111, 113)	**18**
–	Head mostly dark brown to black posteriorly, orange on most of frons and face (Figs 9, 11, 12, 59, 61, 62, 126, 128–130, 180, 183, 184)	**21**
18(17)	Meso- and metasoma entirely yellow, at most with darker (brown) areas on mesoscutellar arm, metanotum and along median longitudinal carina of propodeum (Figs 79–82, 110–115) [Distribution: Brazil, Panama]	**19**
–	Meso- and metasoma at least partially dark brown to black (Figs 136–140, 143–145) [Distribution: Costa Rica, ACG]	**20**
19(18)	T1 narrowing towards posterior margin (Fig. 80); T2 smooth and subtriangular, width at posterior margin twice width at anterior margin; meso- and metasoma entirely yellow (Figs 79–82) [Host: *Lipomerinx prismatica*, Tineidae. Distribution: Panama]	***Pseudapanteles lipomeringis* (Muesebeck, 1958)**
–	T1 barely narrowing towards posterior margin, its length 1.7 × its width at posterior margin (Figs 112, 115); T2 mostly with longitudinal striation and much more transverse (width at posterior margin 1.2 × width at anterior margin) (Fig. 112); darker areas (brown) on mesoscutellar arm, metanotum and along median longitudinal carina of propodeum (Figs 110, 111, 114) [Distribution: Brazil]	***Pseudapanteles moerens* (Nixon, 1965)**
20(18)	Anteromesoscutum mostly orange but with brown marks laterally and centrally on anterior 0.3 (Figs 144, 145), rest of mesosoma orange (Figs 141–143); T2 smooth (Fig. 143)	***Pseudapanteles pedroleoni* Fernández-Triana & Whitfield, sp. n.**
–	Anteromesoscutum entirely orange (Figs 138–140); mesopleuron, metapleuron, axillar complex, metascutellum and propodeum dark brown to black (Figs 136–140); T2 mostly longitudinally striate (Fig. 137)	***Pseudapanteles ottonsolisi* Fernández-Triana & Whitfield, sp. n.**
21(17)	Most of mesosoma (except for metanotum and propodeum black), metasoma and legs reddish brown (Figs 126–130) [Distribution: United States (Georgia and Pennsylvania)]	***Pseudapanteles nigrovariatus* (Muesebeck, 1921)**
–	Mesosoma, metasoma and legs with different color patterns	**22**
22(21)	Metacoxa dark brown to black (Figs 59, 60, 64, 180, 185); ovipositor sheaths at least 1.0 × as long as metatibia (Figs 59, 180, 182); T1 length at most 2.3 × its width at posterior margin; T1 maximum width (reached at approximately half length of tergite) 1.8 × its width at posterior margin	**23**
–	All coxae yellow (Figs 9, 13); ovipositor sheaths at most 0.9 × as long as metatibia (Figs 9); T1 length 3.4–5.0 × its width at posterior margin; T1 maximum width (reached at approximately half length of tergite) 2.3–2.9 × its width at posterior margin	**24**
23(22)	Body length 2.8 mm, fore wing length 3.0 mm; pterostigma brown with anterior spot pale (Fig. 181); T1 length 1.8 × its width at posterior margin (Figs 182, 185); T2 smooth, its width at posterior margin 4.5 × its length [Host: unidentified Gelechiidae]	***Pseudapanteles teofilodelatorrei* Fernández-Triana & Whitfield, sp. n.**
–	Body length 2.5 mm, fore wing length 2.7 mm; pterostigma pale with thin brown margins (Fig. 60); T1 length 2.3 × its width at posterior margin (partially seen in Figs 60, 61, 64); T2 mostly longitudinally striate, its width at posterior margin 3.4 × its length (Fig. 64)	***Pseudapanteles hernanbravoi* Fernández-Triana & Whitfield, sp. n.**
24(22)	All flagellomeres brown (Figs 166–168); T2 light brown (Figs 167, 168); metatibia yellow, with posterior 0.1–0.2 dark brown to black, metatarsus dark brown to black (Figs 167, 168); pterostigma pale, with thin brown margins (Fig. 168); ovipositor sheaths 0.9 × as long as metatibia (Figs 167, 168) [Hosts: *Desmia* spp., *Spoladea recurvalis*, Crambidae]	***Pseudapanteles ruficollis* (Cameron, 1911)**
–	Central flagellomeres white-yellow, rest dark brown to black (Figs 9, 11, 24, 28, 29); T2 orange-yellow (Figs 13, 26); metatibia and metatarsus yellow (Figs 10, 13, 24); pterostigma entirely dark or brown with anterior spot pale (Figs 10, 25); ovipositor sheaths 0.7–0.8 × as long as metatibia (Figs 9, 24)	**25**
25(24)	T2 mostly longitudinally striate; antenna brown, with flagellomeres 4–8 white, white band clearly occupying more than one third of antenna length (Fig. 9)	***Pseudapanteles alfiopivai* Fernández-Triana & Whitfield, sp. n.**
–	T2 mostly smooth and polished; antenna brown with flagellomeres 6–8 white (rarely also posterior half of flagellomere 5), white band clearly occupying less than one third of antenna length (Figs 24, 28, 29)	**26**
26(25)	Metasoma entirely orange-yellow (Figs 24–27) [Distribution: Panama, St. Vincent]	***Pseudapanteles annulicornis* Ashmead, 1900**
–	Metasoma with T3+ partially brown (Figs 85, 88, 91, 93) [Distribution: Costa Rica, ACG]	**27**
27(26)	Mesosoma mostly orange-yellow but with darker areas on propodeum, metapleuron, metascutellum and axillar complex (darker areas not clearly visible in holotype illustrated in Figs 89–93, but clearly marked on most other specimens)	***Pseudapanteles margaritapenonae* Fernández-Triana & Whitfield, sp. n.**
–	Mesosoma entirely orange-yellow (Fig. 84–88)	***Pseudapanteles luisguillermosolisi* Fernández-Triana & Whitfield, sp. n.**
28(2)	Pterostigma yellow-white, with very thin brown margins (Fig. 106); ***and*** propodeum only slightly sculptured on posterolateral corners (Fig. 109); ***and*** propodeum differentiated into elevated central area (which is shiny) and depressed posterolateral corners; ***and*** metasoma tergites dark brown except anterior 0.6 of T1 yellow (Figs 106, 107, 109)	***Pseudapanteles maureenballesteroae* Fernández-Triana & Whitfield, sp. n.**
–	Not as above, ***either*** pterostigma entirely or mostly brown (at most with small pale spot anteriorly); ***or*** propodeum dull and mostly sculptured, covered by numerous transverse rugosities in addition to well-defined median carina (as in Figs 55, 58); ***or*** metasoma tergites with different coloration (mostly yellow ***or*** entirely dark brown to black)	**29**
29(28)	Mesosoma and metasoma (dorsally) entirely dark brown to black (Figs 37–41)	***Pseudapanteles carlosrodriguezi* Fernández-Triana & Whitfield, sp. n.**
–	Either mesosoma or metasoma with extensive yellow to orange areas (Figs 55, 58)	**30**
30(29)	Metasomal tergites mostly yellow except dark brown T1 and light brown T2 (Figs 55, 58); mesosoma entirely black (Figs 53–55, 58) [Host: *Paraclemensia acerifoliella*, Incurvariidae. Distribution: Nearctic, Canada]	***Pseudapanteles gouleti* Fernández-Triana, 2010**
–	Metasomal tergites entirely dark brown to black; mesosoma usually with at least a small area orange [Distribution: Neotropical, Brazil and Costa Rica (ACG)]	**31**
31(30)	T1 almost parallel-sided, its length 2.4 × its width apically, and its maximum width (at approximately half length of tergite) 1.3 × width at posterior margin (Fig. 131); pronotal collar yellow-orange (Figs 131, 134); anteromesoscutum entirely orange-yellow (Figs 131, 134, 135) [Hosts: *Antaeotricha* (Elachistidae), two other unidentified Elachistidae]	***Pseudapanteles oscarariasi* Fernández-Triana & Whitfield, sp. n.**
–	T1 clearly narrowing towards posterior margin, its length at least 3.3 × its width apically (usually much more), and its maximum width (at approximately half length of tergite) at least 1.9 × width at posterior margin (Figs 5, 150, 165, 176); pronotal collar dark brown to black (Figs 7, 162); anteromesoscutum entirely or mostly dark brown to black (Figs 148, 150, 165, 174, 175, 178), if mostly orange, then at least with brown spot centrally on anterior 0.2 (as partially seen in Fig. 8)	**32**
32(31)	Anteromesoscutum mostly orange, with only brown spot centrally on anterior 0.2 (as partially seen in Fig. 8) [Distribution: Brazil]	***Pseudapanteles abantidas* (Nixon, 1965)**
–	Anteromesoscutum entirely or mostly dark brown to black (Figs 148, 150, 174, 175, 178)	**33**
33(32)	Propodeum clearly differentiated into elevated central area and depressed posterolateral corners (Fig. 177); propodeum mostly smooth, with well-defined median carina and few short rugosities transverse to that carina (Fig. 177); antenna relatively shorter on its anterior half, with flagellomere 2 2.5 × as long as wide, and flagellomere 8 2.2 × as long as wide (Figs 174, 178)	***Pseudapanteles soniapicadoae* Fernández-Triana & Whitfield, sp. n.**
–	Propodeum not differentiated into elevated central area and depressed posterolateral corners (Figs 148, 150, 164, 165); propodeum dull and mostly sculptured, covered by numerous transverse rugosities in addition to well-defined median carina (Figs 148, 150, 164, 165); antenna relatively longer on its anterior half, with flagellomere 2 at least 3.0 × as long as wide, and flagellomere 8 at least 2.6 × as long as wide (Figs 162, 163)	**34**
34(33)	Anteromesoscutum, axillar complex and head (except for clypeus, labrum and mandibles) entirely dark brown to black (Figs 162, 163, 165); scape brown, same color as flagellomeres (Figs 162, 163)	***Pseudapanteles rosemarykarpinskiae* Fernández-Triana & Whitfield, sp. n.**
–	Anteromesoscutum and axillar complex with some orange spots, head mostly brown-black posteriorly but orange on most of frons and face (Figs 146, 148–150); scape yellow, contrasting with brown flagellomeres (Fig. 149)	***Pseudapanteles raulsolorzanoi* Fernández-Triana & Whitfield, sp. n.**

## Taxonomic treatment

### Pseudapanteles abantidas

Taxon classificationAnimaliaHymenopteraBraconidae

(Nixon, 1965)

Figs 3 –8

Apanteles abantidas Nixon, 1965: 142 (original description).
Pseudapanteles abantidus : Mason 1981: 86 (revised combination).

#### Holotype.

♀ in BMNH (examined). BRAZIL, Nova Teutonia, 27°11'S, 52°23'W, 12.vii.1937, Fritz Plaumann, B.M. 1937-656.

#### Male.

Unknown.

#### Diagnosis.

It belongs to the *gouleti* species-group, and can be separated from other species within that group based on the coloration of anteromesoscutum and metasomal tergites, as well as shape of T1.

#### Comments.

Only the holotype specimen is known.

### Pseudapanteles alfiopivai

Taxon classificationAnimaliaHymenopteraBraconidae

Fernández-Triana & Whitfield sp. n.

http://zoobank.org/30BCA7DB-5CB5-4D9F-826A-1D290355FD5B

Figs 9 –13

#### Holotype.

♀ in CNC. COSTA RICA, ACG, Guanacaste Province, Sector El Hacha, Sendero Bejuquilla, 280m, 11.03004, -85.52699, 17.viii.1998. ACG database code: DHJPAR0012577.

#### Paratypes.

3 ♂ (CNC). COSTA RICA, ACG database codes: DHJPAR0013128, DHJPAR0025345, DHJPAR0031764.

#### Diagnosis.

It belongs to the *annulicornis* species-group, and can be separated from other species within that group based on the combination of flagellomeres 4–8 white-yellow (occupying more than one third of antenna length), head mostly dark brown to black posteriorly, all coxae yellow, and T2 mostly striate.

#### Description.

**Female.** Body length 2.0–2.1 mm. Fore wing length 2.2–2.3 mm. Head color: mostly dark brown to black; except for orange on most of frons and face, and yellow clypeus, labrum, mandibles, and spot on lower corner of gena near oral foramen. Flagellomere color: central flagellomeres white-yellow, rest dark brown to black. Mesosoma color: entirely orange to yellow-orange. Metasoma color (dorsally): entirely orange to yellow-orange. Coxae color: all pale. Metatibia color: pale. Metatarsus color: pale. Pterostigma color: mostly dark, but with anterior pale spot. Mediotergite 1 length/width at posterior margin 4.6–5.0 ×. Mediotergite 1 maximum width/width at posterior margin 2.7–2.8 ×. Mediotergite 2 width at posterior margin/length: 3.2–3.3 ×. Mediotergite 2 sculpture: Mostly with longitudinally striate sculpture (sometimes with small, smooth area centrally). Ovipositor sheaths length: 0.8 × as long as metatibia.

**Male.** As female, but with all flagellomeres brown.

#### Molecular data.

Sequences in BOLD: 4, barcode compliant sequences: 4.

#### Biology/ecology.

Malaise-trapped.

#### Distribution.

Costa Rica, ACG dry forest and rain forest.

#### Etymology.

This species is named in honour of Dr. Alfio Piva, a Costa Rican former Vice-President, in recognition of his many years of administrative support to Costa Rica’s INBio (Instituto Nacional de Biodiversidad) and therefore of ACG, and of his policy efforts on behalf of conserving biodiversity in Costa Rica.

### Pseudapanteles alvaroumanai

Taxon classificationAnimaliaHymenopteraBraconidae

Fernández-Triana & Whitfield sp. n.

http://zoobank.org/679BABD8-BB46-4A4E-9D70-C600623B13CE

Figs 14 –18

#### Holotype.

♀ in CNC. COSTA RICA, ACG, Guanacaste Province, Sector Cacao, Sendero Arenales, 1080m, 10.92471, -85.46738, 18.xii.2008. ACG database code: DHJPAR0031316.

#### Paratypes.

16 ♂ (CNC, NMNH). COSTA RICA, ACG database codes: DHJPAR0013423, DHJPAR0013648, DHJPAR0013654, DHJPAR0026205, DHJPAR0026226, DHJPAR0031220, DHJPAR0033731, DHJPAR0033732, DHJPAR0033734, DHJPAR0033742, DHJPAR0033748, DHJPAR0033768, DHJPAR0033896, DHJPAR0033902, DHJPAR0033903, DHJPAR0033906.

#### Diagnosis.

It belongs to the *annulicornis* species-group, and can be separated from other species within that group based on the combination of flagellomeres brown, coloration of mesosoma (propleuron, pronotum partially, small spot on upper corner of mesopleuron, and small marks centrally on anteromesoscutum orange-yellow, rest dark brown), and metacoxa brown.

#### Description.

**Female.** Body length 2.0–2.1 mm. Fore wing length 2.2–2.3 mm. Head color: mostly dark brown to black; except for orange on most of frons and face, and yellow clypeus, labrum, mandibles, and spot on lower corner of gena near oral foramen. Flagellomere color: all flagellomere brown to black. Mesosoma color: mostly dark brown to black, with pronotum, propleura, anteromesoscutum, spot on mesopleura, and scutellar disc at least partially orange. Metasoma color (dorsally): mostly dark brown to black, except for yellow-orange anterior 0.4–0.6 of mediotergite 1. Coxae color: pale/pale/mostly or completely dark. Metatibia color: mostly pale, with posterior 0.1–0.2 dark. Metatarsus color: pale. Pterostigma color: mostly dark, but with anterior pale spot. Mediotergite 1 length/width at posterior margin 3.6–4.0 ×. Mediotergite 1 maximum width/width at posterior margin 2.5–2.6 ×. Mediotergite 2 width at posterior margin/length: 3.6–3.7 ×. Mediotergite 2 sculpture: Mostly with longitudinally striate sculpture (sometimes with small, smooth area centrally). Ovipositor sheaths length: 1.0 × as long as metatibia.

**Male.** As female, but with coloration of some specimens slightly darker.

#### Molecular data.

Sequences in BOLD: 26, barcode compliant sequences: 26.

#### Biology/ecology.

Malaise-trapped.

#### Distribution.

Costa Rica, ACG cloud forest, dry forest and rain forest.

#### Etymology.

This species is named in honour of Dr. Alvaro Umaña in recognition of his untiring efforts on behalf of ACG from its initiation in 1985–86 to the present day, and from being then Costa Rica’s first Minister of the Environment (MINAE) to today’s global advocate for ACG as an example of conservation through biodiversity development.

### Pseudapanteles analorenaguevarae

Taxon classificationAnimaliaHymenopteraBraconidae

Fernández-Triana & Whitfield sp. n.

http://zoobank.org/8FB50730-FD59-46C8-8F5C-D023BA01E0F5

Figs 19 –23

#### Holotype.

♀ in CNC. COSTA RICA, ACG, Guanacaste Province, Sector Cacao, Sendero Circular, 1185 meters, 10.92714, -85.46683, 18.xii.2008. ACG database code: DHJPAR0031187.

#### Paratypes.

11 ♂ (CNC). COSTA RICA, ACG database codes: DHJPAR0013526, DHJPAR0031186, DHJPAR0031191, DHJPAR0031192, DHJPAR0031193, DHJPAR0031202, DHJPAR0031204, DHJPAR0031209, DHJPAR0031302, DHJPAR0031307, DHJPAR0031321.

#### Diagnosis.

It belongs to the *annulicornis* species-group, and can be separated from other species within that group based on the combination of head brown, flagellomeres 7–9 white, anteromesoscutum with orange marks centrally, and metatarsus and posterior 0.2 of metatibia brown.

#### Description.

**Female.** Body length 2.8–2.9 mm. Fore wing length 3.0–3.1 mm. Head color: mostly dark brown to black, except for yellow clypeus, labrum, mandibles, and spot on lower corner of gena near oral foramen. Flagellomere color: central flagellomere white-yellow, rest dark brown to black. Mesosoma color: mostly dark brown to black, with pronotum, propleura, anteromesoscutum, spot on mesopleura, and scutellar disc at least partially orange. Metasoma color (dorsally): mostly dark brown to black, except for yellow-orange anterior 0.4–0.6 of mediotergite 1. Coxae color: pale/pale/pale but with anterior 0.1–0.2 dark. Metatibia color: mostly pale, with posterior 0.1–0.2 dark. Metatarsus color: dark. Pterostigma color: mostly dark, but with anterior pale spot. Mediotergite 1 length/width at posterior margin 2.6–3.0 ×. Mediotergite 1 maximum width/width at posterior margin 1.7–1.8 ×. Mediotergite 2 width at posterior margin/length: 3.4–3.5 ×. Mediotergite 2 sculpture: Mostly with longitudinally striate sculpture (sometimes with small, smooth area centrally). Ovipositor sheaths length: 1.0 × as long as metatibia.

**Male.** As female, but with all flagellomeres brown, and sometimes anteromesoscutum and metasoma darker.

#### Molecular data.

Sequences in BOLD: 20, barcode compliant sequences: 20.

#### Biology/ecology.

Malaise-trapped.

#### Distribution.

Costa Rica, ACG cloud forest.

#### Etymology.

This species is named in honour of Sra. Ana Lorena Guevara, a key figure in INBio’s (Instituto Nacional de Biodiversidad) development of biodiversity prospecting, and a Vice-Minister for the Environment who supported ACG growth and development throughout her four years in office in the 2010’s.

### Pseudapanteles annulicornis

Taxon classificationAnimaliaHymenopteraBraconidae

Ashmead, 1900

Figs 24 –31

Pseudapanteles annulicornis Ashmead, 1900: 292 (original description).
Apanteles annulicornis : Szépligeti 1904: 109 (revised combination).
Pseudapanteles annulicornis : Mason 1981: 86 (revised combination).
Pseudapanteles brunneus Ashmead, 1900: 292 (original description). Synonymized under *annulicornis* (as *Apanteles brunneus*) in Wilkinson 1930: 154.

#### Holotype.

♀ in BMNH (examined). ST. VINCENT AND THE GRENADINES, St. Vincent Island (no further details known about type locality). B.M. Type HYM. 3.c.1077.

#### Other material examined.

2 ♀ in CNC, St. Vincent island; 1 ♀ in CNC, Panama, Cerro Campana, 8°40'N, 79°50'W, 850m.

#### Diagnosis.

It belongs to the *annulicornis* species-group, and can be separated from other species within that group based on the combination of metasoma entirely orange-yellow, mostly smooth and polished, and antenna brown with flagellomeres 6–8 white (rarely also posterior half of flagellomere 5), the band clearly occupying less than one third of antenna length.

#### Molecular data.

Sequences in BOLD: 2, barcode compliant sequences: None.

#### Distribution.

Panama, St. Vincent Island.

#### Comments.

One female from Panama (in CNC collection) represents the first record of *Pseudapanteles annulicornis* for Central America, and suggests that the species might be more widespread than previously known. That specimen is morphologically very similar to the holotype and two other females from St. Vincent (not part of the type series; collected in 1972, and deposited in the CNC); the only difference observed was the white band on the antenna (starting on flagellomere 5 for the Panama specimen versus starting on flagellomere 6 on specimens from St. Vincent). Two partial DNA barcodes (99 and 164 base pairs respectively) were obtained and also support the view of a single species, as the partial barcodes differed in 2 base pairs between the Panama specimen and one of the females collected in 1972 in St. Vincent.

### Pseudapanteles brunneus

Taxon classificationAnimaliaHymenopteraBraconidae

Ashmead, 1900

Pseudapanteles brunneus Ashmead, 1900: 292 (original description).
Apanteles brunneus : Szépligeti, 1904: 109 (revised combination).
Apanteles annulicornis : Wilkinson 1930: 154 (synonymized with *Pseudapanteles annulicornis* Ashmead, 1900).
Apanteles brunneus : Nixon 1965: 141 (revised status, see comments below).
Pseudapanteles brunneus : Mason 1981: 86 (revised combination).

#### Holotype.

♂ in BMNH (not examined). ST. VINCENT AND THE GRENADINES, St. Vincent Island (no further details known about the holotype locality).

#### Comments.

Only the holotype specimen is known. Wilkinson (1930) synonymized *Apanteles brunneus* with *Apanteles annulicornis*, but that was later questioned by Nixon (1965) after examining the hind wings of both holotypes. Nixon implied reinstatement of *Apanteles brunneus* as a valid species but he did not revise its status explicitly. However, Mason (1981: 86) treated *brunneus* as a valid species of *Pseudapanteles*. This is the only species that we were not able to photograph for the present revision.

### Pseudapanteles carlosespinachi

Taxon classificationAnimaliaHymenopteraBraconidae

Fernández-Triana & Whitfield sp. n.

http://zoobank.org/62386CBD-D249-43FA-A0B4-D2173A86C771

Figs 32 –36

#### Holotype.

♂ in CNC. COSTA RICA, ACG, Alajuela Province, Sector San Cristobal, Rio Blanco Abajo, 500m, 10.90037, -85.37254, 11.iii.2006. ACG database code: DHJPAR0004755.

#### Other material examined.

1 ♀ (CNC). COSTA RICA, ACG database codes: DHJPAR0039928.

#### Diagnosis.

It belongs to the *annulicornis* species-group, and can be separated from other species within that group based on the combination of head, flagellomeres, mesosoma and metasoma mostly dark brown to black, body length and fore wing length 2.3–2.5 mm, and shape of T1 and T2.

#### Description.

**Male.** Body length 2.0–2.1 mm. Fore wing length 2.2–2.3 mm. Head color: mostly dark brown to black, except for yellow clypeus, labrum, mandibles, and spot on lower corner of gena near oral foramen. Mesosoma color: entirely dark brown to black. Metasoma color (dorsally): mostly dark brown to black, except for yellow-orange anterior 0.4–0.6 of mediotergite 1. Coxae color: pale/pale/mostly or completely dark. Metatibia color: mostly pale, with posterior 0.1–0.2 dark. Metatarsus color: dark. Pterostigma color: pale, with thin dark margins.

**Female.** See Comments section below.

#### Molecular data.

Sequences in BOLD: 3, barcode compliant sequences: 2.

#### Biology/ecology.

Hosts: *Desmia* sp. with interim name of Solis100 (Crambidae).

#### Distribution.

Costa Rica, ACG rain forest.

#### Etymology.

This species is named in honour of Sr. Carlos Espinach in recognition of his economic policy efforts for Costa Rica’s government since the early 1990’s, all of which greatly enhanced ACG growth and survival since the mid-1980’s.

#### Comments.

Only the holotype male was used to morphologically characterize the species. The female specimen available for study was in poor condition and entirely bleached, with a coloration that most likely does not represent the actual females of this species.

### Pseudapanteles carlosrodriguezi

Taxon classificationAnimaliaHymenopteraBraconidae

Fernández-Triana & Whitfield sp. n.

http://zoobank.org/1C6E1FE6-BF98-4D25-9B06-C372062EBDC0

Figs 37 –41

#### Holotype.

♀ in CNC. COSTA RICA, ACG, Guanacaste Province, Sector El Hacha, Sendero Bejuquilla, 280m, 11.03004, -85.52699, 3.v.1999. ACG database code: DHJPAR0012576.

#### Paratypes.

2 ♂ (CNC). COSTA RICA, ACG database codes: DHJPAR0013545, DHJPAR0013549.

#### Diagnosis.

It belongs to the *gouleti* species-group, and can be separated from other species within that group based on the combination of mesosoma and metasoma (dorsally) entirely dark brown to black.

#### Description.

**Female.** Body length 2.0–2.1 mm. Fore wing length 2.0–2.1 mm. Head color: mostly dark brown to black, except for yellow clypeus, labrum, mandibles, and spot on lower corner of gena near oral foramen. Flagellomere color: all flagellomere brown to black. Mesosoma color: entirely dark brown to black. Metasoma color (dorsally): entirely dark brown to black. Coxae color: all pale. Metatibia color: mostly pale, with posterior 0.1–0.2 dark. Metatarsus color: dark. Pterostigma color: mostly dark, but with anterior pale spot. Mediotergite 1 length/width at posterior margin 3.1–3.5 ×. Mediotergite 1 maximum width/width at posterior margin 2.1–2.2 ×. Mediotergite 2 width at posterior margin/length: 3.2–3.3 ×. Mediotergite 2 sculpture: Mostly with longitudinally striate sculpture (sometimes with small, smooth area centrally). Ovipositor sheaths length: 1.0 × as long as metatibia.

**Male.** As female, with slightly darker body coloration.

#### Molecular data.

Sequences in BOLD: 4, barcode compliant sequences: 4.

#### Biology/ecology.

Malaise-trapped.

#### Distribution.

Costa Rica, ACG dry forest.

#### Etymology.

This species is named in honour of Sr. Carlos Manuel Rodriguez, who has faithfully supported ACG policy for conservation through its biodiversity development from the early 1990’s through the present day, and has been especially important for the development of geothermal resources and land acquisition as a legal advisor, Vice-Minister and Minister of the Environment, all with special and detailed knowledge of Sector Santa Rosa of ACG.

### Pseudapanteles christianafigueresae

Taxon classificationAnimaliaHymenopteraBraconidae

Fernández-Triana & Whitfield sp. n.

http://zoobank.org/759313A8-A5FE-4CBF-AB5E-2796421F25B3

Figs 42 –47

#### Holotype.

♀ in CNC. COSTA RICA, Alajuela Province, ACG, Sector San Cristobal, Bosque Trampa Malaise, 815m, 10.86280, -85.38460, 13.xii.2007. ACG database code: DHJPAR0025960.

#### Paratypes.

17 ♀, 41 # (BMNH, CNC, INBio, INHS, NMNH). COSTA RICA, ACG database codes: DHJPAR0024675, DHJPAR0024987, DHJPAR0025702, DHJPAR0025895, DHJPAR0025902, DHJPAR0025985, DHJPAR0025966, DHJPAR0026012, DHJPAR0026026, DHJPAR0026065, DHJPAR0026081, DHJPAR0026084, DHJPAR0026088, DHJPAR0026652, DHJPAR0026717, DHJPAR0027075, DHJPAR0027138, DHJPAR0027164, DHJPAR0027242, DHJPAR0027392, DHJPAR0027591, DHJPAR0027621, DHJPAR0027627, DHJPAR0027646, DHJPAR0027655, DHJPAR0027656, DHJPAR0027657, DHJPAR0027658, DHJPAR0027661, DHJPAR0027663, DHJPAR0027664, DHJPAR0027667, DHJPAR0027699, DHJPAR0027670, DHJPAR0027673, DHJPAR0027675, DHJPAR0027689, DHJPAR0027690, DHJPAR0027691, DHJPAR0027693, DHJPAR0027698, DHJPAR0027700, DHJPAR0027701, DHJPAR0031297.

#### Diagnosis.

It belongs to the *annulicornis* species-group, and can be separated from other species within that group based on the combination of head mostly brown-black posteriorly, but orange on most of frons and face, flagellomeres 6–10 (and posterior half of flagellomere 5) white, anteromesoscutum entirely brown to black, and metatibia and metatarsus entirely yellow to orange.

#### Description.

**Female.** Body length 2.4–2.5 mm or 2.6–2.7 mm. Fore wing length 2.6–2.7 mm. Head color: mostly dark brown to black; except for orange on most of frons and face, and yellow clypeus, labrum, mandibles, and spot on lower corner of gena near oral foramen. Flagellomere color: central flagellomere white-yellow, rest dark brown to black. Mesosoma color: entirely dark brown to black. Metasoma color (dorsally): mostly dark brown to black, except for yellow-orange anterior 0.4–0.6 of mediotergite 1. Coxae color: pale/pale/pale but with anterior 0.1–0.2 dark. Metatibia color: pale, rarely mostly pale, with posterior 0.1–0.2 dark. Metatarsus color: pale, rarely dark. Pterostigma color: entirely dark. Mediotergite 1 length/width at posterior margin 4.1–4.5 ×. Mediotergite 1 maximum width/width at posterior margin 2.3–2.4 ×. Mediotergite 2 width at posterior margin/length: 3.6–3.7 × or 3.8–3.9 ×. Mediotergite 2 sculpture: Mostly with longitudinally striate sculpture (sometimes with small, smooth area centrally). Ovipositor sheaths length: 1.0 × as long as metatibia or 1.1 × as long as metatibia.

**Male.** As female, but with all flagellomeres brown.

#### Molecular data.

Sequences in BOLD: 47, barcode compliant sequences: 38.

#### Biology/ecology.

Malaise-trapped.

#### Distribution.

Costa Rica, ACG rain forest.

#### Etymology.

This species is named in honour of Sra. Christiana Figueres for her persistent interest in ACG survival since the early 1990’s, and up through her magnificent current efforts to get the world to reverse its climate change via the UN organizational capacity.

### Pseudapanteles dignus

Taxon classificationAnimaliaHymenopteraBraconidae

(Muesebeck, 1938)

Figs 48 –52

Apanteles dignus Muesebeck, 1938: 203 (original description).
Apanteles dignus : Shenefelt 1972: 492 (incorrect mention of date of original description as 1928).
Pseudapanteles dignus : Mason 1981: 86 (revised combination).

#### Holotype.

♀ in NMNH (examined). UNITED STATES, California, Santa Ana. USNM type No. 52890.

#### Other material examined.

2 ♂ in CNC, Mexico (intercepted in Canada, Ontario, Windsor).

#### Diagnosis.

It belongs to the *annulicornis* species-group, and can be separated from other species within that group based on the combination of mesosoma and coxae color, anterior 0.6 of mediotergite 1 and most of laterotergites orange-yellow, pterostigma transparent with thin margins brown and T1 shape.

#### Molecular data.

Sequences in BOLD: 1, barcode compliant sequences: 1.

#### Biology/ecology.

Hosts: *Keiferia lycopersicella*, *Phthorimaea operculella*, *Symmetrischema capsica*, *Tildenia gudmannella*, *Tuta absoluta* (Gelechiidae).

#### Distribution.

Argentina, Bermuda, Cuba, Mexico, Puerto Rico, United States (California, Florida, introduced to Hawaii), US Virgin Islands.

#### Comments.

In the CNC there are two male specimens (March, 1936, from Windsor, Ontario Canada); their labels state that the wasp specimens were intercepted on tomato from Mexico infested by *Keiferia lycopersicella*. Due to the known distribution of the species (mostly Neotropical, only marginally reaching the southern Nearctic), and the fact that those specimens were intercepted during the Canadian winter, we have no evidence that the species occurs in Canada.

### Pseudapanteles gouleti

Taxon classificationAnimaliaHymenopteraBraconidae

Fernández-Triana, 2010

Figs 53 –58

Pseudapanteles gouleti Fernández-Triana, 2010: 23 (original description).

#### Holotype.

♀ in CNC (examined). CANADA, Ontario, Ottawa, 45°21.365'N, 75°42.416'W.

#### Other material examined.

All specimens mentioned in Fernández-Triana (2010 and 2014).

#### Diagnosis.

It belongs to the *gouleti* species-group, and can be separated from other species within that group based on the combination of mesosoma entirely black and metasomal tergites mostly yellow except dark brown T1 and light brown T2.

#### Molecular data.

Sequences in BOLD: 15, barcode compliant sequences: 13.

#### Biology/ecology.

Hosts: *Paraclemensia acerifoliella* (Incurvariidae).

#### Distribution.

Canada (Ontario in an area between 43–46°N and 74–80°W).

#### Comments.

The species was recently proposed as of interest for conservation purposes (Fernández-Triana 2014).

### Pseudapanteles hernanbravoi

Taxon classificationAnimaliaHymenopteraBraconidae

Fernández-Triana & Whitfield sp. n.

http://zoobank.org/9F00A887-B7AA-42F1-8F96-94497AA5AF33

Figs 59 –64

#### Holotype.

♀ in CNC. COSTA RICA, ACG, Guanacaste Province, Sector Santa Rosa, Bosque San Emilio, 300m, 10.84389, -85.61384, 19.iv.1999. ACG database code: DHJPAR0013144.

#### Paratypes.

1 ♀ (CNC). COSTA RICA, ACG database codes: DHJPAR0031742.

#### Diagnosis.

It belongs to the *annulicornis* species-group, and can be separated from other species within that group based on the combination of body length 2.5 mm, fore wing length 2.7 mm; pterostigma pale with thin brown margins, T1 shape and T2 mostly longitudinally striate.

#### Description.

**Female.** Body length 2.4–2.5 mm. Fore wing length 2.6–2.7 mm. Head color: mostly dark brown to black; except for orange on most of frons and face, and yellow clypeus, labrum, mandibles, and spot on lower corner of gena near oral foramen. Flagellomere color: first 6–8 flagellomere lighter in color than the rest (which are dark brown to black), making the antenna look as bicolored. Mesosoma color: mostly orange, with parts or all of propodeum, metapleuron, metascutellum, and axillar complex brown to black. Metasoma color (dorsally): mostly yellow-orange, except for mediotergites 4–7 which are centrally brown. Coxae color: pale/pale/mostly or completely dark. Metatibia color: mostly pale, with posterior 0.1–0.2 dark. Metatarsus color: dark. Pterostigma color: pale, with thin dark margins. Mediotergite 1 length/width at posterior margin 2.1–2.5 ×. Mediotergite 1 maximum width/width at posterior margin 1.7–1.8 ×. Mediotergite 2 width at posterior margin/length: 3.4–3.5 ×. Mediotergite 2 sculpture: Mostly with longitudinally striate sculpture (sometimes with small, smooth area centrally). Ovipositor sheaths length: 1.1 × as long as metatibia.

**Male.** Unknown.

#### Molecular data.

Sequences in BOLD: 2, barcode compliant sequences: 2.

#### Biology/ecology.

Malaise-trapped.

#### Distribution.

Costa Rica, ACG dry forest.

#### Etymology.

This species is named in honour of Sr. Hernan Bravo, who, as a Costa Rican Minister of the Environment and later, directly and indirectly supported ACG’s conservation through its biodiversity development, and especially with respect to geothermal resources

### Pseudapanteles jorgerodriguezi

Taxon classificationAnimaliaHymenopteraBraconidae

Fernández-Triana & Whitfield sp. n.

http://zoobank.org/0B34D64D-441E-42D0-9FEC-A4F87D24D964

Figs 65 –69

#### Holotype.

♀ in CNC. COSTA RICA, ACG, Alajuela Province, Sector San Cristobal, Estacion San Gerardo, 575m, 10.88009, -85.38887, 22.vii.2007. ACG database code: DHJPAR0025854.

#### Diagnosis.

It belongs to the *annulicornis* species-group, and can be separated from other species within that group based on the combination of ovipositor sheaths length, T2 mostly longitudinally striate (except for small smooth central area), propodeum mostly smooth (with only median longitudinal carina), and scutoscutellar sulcus with 6 impressed pits.

#### Description.

**Female.** Body length 2.0–2.1 mm. Fore wing length 2.2–2.3 mm. Head color: mostly dark brown to black, except for yellow clypeus, labrum, mandibles, and spot on lower corner of gena near oral foramen. Flagellomere color: all flagellomere brown to black. Mesosoma color: entirely dark brown to black. Metasoma color (dorsally): mostly dark brown to black, except for yellow-orange anterior 0.4–0.6 of mediotergite 1. Coxae color: pale/pale/mostly or completely dark. Metatibia color: mostly pale, with posterior 0.1–0.2 dark. Metatarsus color: dark. Pterostigma color: entirely dark. Mediotergite 1 length/width at posterior margin 4.1–4.5 ×. Mediotergite 1 maximum width/width at posterior margin 2.1–2.2 ×. Mediotergite 2 width at posterior margin/length: 4.0–4.1 ×. Mediotergite 2 sculpture: Mostly with longitudinally striate sculpture (sometimes with small, smooth area centrally). Ovipositor sheaths length: 0.9 × as long as metatibia.

**Male.** Unknown.

#### Molecular data.

Sequences in BOLD: 1, barcode compliant sequences: 1.

#### Biology/ecology.

Malaise-trapped.

#### Distribution.

Costa Rica, ACG rain forest.

#### Etymology.

This species is named in honour of Sr. Jorge Rodriguez, who as a forester and a Costa Rican Vice-Minister and Minister of the Environment helped ACG forge new paths of self-support through Environmental Service Payments (Pagos para Servicios Ambientales).

### Pseudapanteles josefigueresi

Taxon classificationAnimaliaHymenopteraBraconidae

Fernández-Triana & Whitfield sp. n.

http://zoobank.org/CC82E7D6-0250-4318-9C17-6A8BC695AE27

Figs 70 –73

#### Holotype.

♀ in CNC. COSTA RICA, ACG, Alajuela Province, Sector San Cristobal, Potrero Argentina, 520m, 10.89021, -85.38803, 16.vi.2007. ACG database code: DHJPAR0025751.

#### Diagnosis.

It belongs to the *annulicornis* species-group, and can be separated from other species within that group based on the combination of relatively short ovipositor sheaths (0.7 × as long as metatibia) and T1 shape (T1 length 4.0 × its width at posterior margin).

#### Description.

**Female.** Body length 2.0–2.1 mm. Fore wing length 2.2–2.3 mm. Head color: mostly dark brown to black, except for yellow clypeus, labrum, mandibles, and spot on lower corner of gena near oral foramen. Flagellomere color: all flagellomere brown to black. Mesosoma color: entirely dark brown to black. Metasoma color (dorsally): mostly dark brown to black, except for yellow-orange anterior 0.4–0.6 of mediotergite 1. Coxae color: pale/pale/mostly or completely dark. Metatibia color: mostly pale, with posterior 0.1–0.2 dark. Metatarsus color: dark. Pterostigma color: entirely dark. Mediotergite 1 length/width at posterior margin 3.6–4.0 ×. Mediotergite 1 maximum width/width at posterior margin 2.1–2.2 ×. Mediotergite 2 width at posterior margin/length: 4.0–4.1 ×. Mediotergite 2 sculpture: Mostly with longitudinally striate sculpture (sometimes with small, smooth area centrally). Ovipositor sheaths length: 0.7 × as long as metatibia.

**Male.** Unknown.

#### Molecular data.

Sequences in BOLD: 1, barcode compliant sequences: 1.

#### Biology/ecology.

Malaise-trapped.

#### Distribution.

Costa Rica, ACG rain forest.

#### Etymology.

This species is named in honour of Costa Rica’s former President Jose Maria Figueres in recognition of his steady and imaginative support of ACG foundation, growth and survival through non-damaging biodiversity development, beginning in the late 1980’s and continuing to the present day.

### Pseudapanteles laurachinchillae

Taxon classificationAnimaliaHymenopteraBraconidae

Fernández-Triana & Whitfield sp. n.

http://zoobank.org/AA6DCACA-9954-4AE4-B53B-C0F8BE06BD19

Figs 74 –78

#### Holotype.

♀ in CNC. COSTA RICA, ACG, Alajuela Province, Sector San Cristobal, Potrero Argentina, 520m, 10.89021, -85.38803, 20.ix.2007. ACG database code: DHJPAR0025675.

#### Paratypes.

1 ♀ (CNC). COSTA RICA, ACG database codes: DHJPAR0026060.

#### Diagnosis.

It belongs to the *annulicornis* species-group, and can be separated from other species within that group based on the combination of metasoma yellow-orange on anterior 0.5–0.6 of T1 and most of laterotergites and hypopygium, T2 shape and ovipositor sheaths slightly shorter (0.9 ×) than metatibia.

#### Description.

**Female.** Body length 2.0–2.1 mm or 2.2–2.3 mm. Fore wing length 2.2–2.3 mm. Head color: mostly dark brown to black, except for yellow clypeus, labrum, mandibles, and spot on lower corner of gena near oral foramen. Flagellomere color: all flagellomere brown to black. Mesosoma color: entirely dark brown to black. Metasoma color (dorsally): mostly dark brown to black, except for yellow-orange anterior 0.4–0.6 of mediotergite 1. Coxae color: all pale. Metatibia color: mostly pale, with posterior 0.1–0.2 dark. Metatarsus color: dark. Pterostigma color: entirely dark. Mediotergite 1 length/width at posterior margin 4.1–4.5 ×. Mediotergite 1 maximum width/width at posterior margin 2.1–2.2 ×. Mediotergite 2 width at posterior margin/length: 3.6–3.7 ×. Mediotergite 2 sculpture: Mostly with longitudinally striate sculpture (sometimes with small, smooth area centrally). Ovipositor sheaths length: 0.9 × as long as metatibia.

**Male.** Unknown.

#### Molecular data.

Sequences in BOLD: 2, barcode compliant sequences: 1.

#### Biology/ecology.

Malaise-trapped.

#### Distribution.

Costa Rica, ACG rain forest.

#### Etymology.

This species is named in honour of Sra. Laura Chinchilla, the first female president of Costa Rica and in gratitude for her persistent tolerance of ACG efforts to push the conservation envelope during her term in office.

### Pseudapanteles lipomeringis

Taxon classificationAnimaliaHymenopteraBraconidae

(Muesebeck, 1958)

Figs 79 –83

Apanteles lipomeringis Muesebeck, 1958: 433 (original description).
Pseudapanteles lipomeringis : Mason 1981: 86 (revised combination).

#### Holotype.

♀ in NMNH (examined). PANAMA, Summit, Canal Zone. USNM type No. 2793.

#### Diagnosis.

It belongs to the *annulicornis* species-group, and can be separated from other species within that group based on the combination of mesosoma and metasoma entirely yellow, and shape of T1 and T2.

#### Biology/ecology.

Host: *Lipomerinx prismatica* (Tineidae).

#### Distribution.

Panama.

### Pseudapanteles luisguillermosolisi

Taxon classificationAnimaliaHymenopteraBraconidae

Fernández-Triana & Whitfield sp. n.

http://zoobank.org/BB44CB0B-B2D1-4F26-8D3E-0991551E6571

Figs 84 –88

#### Holotype.

♀ in CNC. COSTA RICA, ACG, Alajuela Province, Sector San Cristobal, Bosque Trampa Malaise, 815m, 10.86280, -85.38460, 11.iii.2008. ACG database code: DHJPAR0027669.

#### Paratypes.

1 ♂ (CNC). COSTA RICA, ACG database codes: 08-SRNP-3967.

#### Diagnosis.

It belongs to the *annulicornis* species-group, and can be separated from other species within that group based on the combination of T2 mostly smooth and polished, antenna brown with flagellomeres 6–8 white (rarely also posterior half of flagellomere 5, white band clearly occupying less than one third of antenna length), and metasoma with T3+ partially brown. Those features are shared with the morphologically similar *Pseudapanteles margaritapenonae*, but *Pseudapanteles luisguillermosolisi* has the mesosoma entirely orange-yellow (while *margaritapenonae* has darker areas on propodeum, metapleuron, metascutellum and axillar complex).

#### Description.

**Female.** Body length 2.4–2.5 mm. Fore wing length 2.6–2.7 mm. Head color: mostly dark brown to black; except for orange on most of frons and face, and yellow clypeus, labrum, mandibles, and spot on lower corner of gena near oral foramen. Flagellomere color: central flagellomere white-yellow, rest dark brown to black. Mesosoma color: entirely orange to yellow-orange. Metasoma color (dorsally): mediotergites 1–2 orange-yellow, rest of mediotergites brown. Coxae color: all pale. Metatibia color: pale. Metatarsus color: pale. Pterostigma color: mostly dark, but with anterior pale spot. Mediotergite 1 length/width at posterior margin 4.1–4.5 ×. Mediotergite 1 maximum width/width at posterior margin 2.3–2.4 ×. Mediotergite 2 width at posterior margin/length: 4.0–4.1 ×. Mediotergite 2 sculpture: Mostly smoth and polished. Ovipositor sheaths length: 0.7 × as long as metatibia.

**Male.** The only known specimen is missing its head, but the coloration of mesosoma and metasoma is darker than the female holotype.

#### Molecular data.

Sequences in BOLD: 1, barcode compliant sequences: 1.

#### Biology/ecology.

Malaise-trapped.

#### Distribution.

Costa Rica, ACG rain forest.

#### Etymology.

This species is named in honour of Sr. Luis Guillermo Solis, the newly-elected President of Costa Rica, and in appreciation of the new opportunity for further administrative evolution that his election offers to ACG in its quest for sustainable conservation through self-directed non-damaging biodiversity development.

#### Comments.

*Pseudapanteles luisguillermosolisi* is morphologically similar to *Pseudapanteles margaritapenonae*, but the mesosoma is entirely orange yellow in the former compared to orange yellow but with darker areas on the propodeum, metapleuron, metascutellum and axillar complex in the latter. Also, these species are at least 25 base pairs different (4%) in the DNA barcoding region.

### Pseudapanteles margaritapenonae

Taxon classificationAnimaliaHymenopteraBraconidae

Fernández-Triana & Whitfield sp. n.

http://zoobank.org/67A1080E-DD85-48C7-92B5-A3F422FB7AA4

Figs 89 –93

#### Holotype.

♀ in CNC. COSTA RICA, Alajuela Province, ACG, Sector San Cristobal, Rio Blanco Abajo, 500m, Latitude: 10.90037, Longitude: -85.37254, 12.iii.2008. ACG database code: DHJPAR0026704.

#### Paratypes.

13 ♀, 141 ♂ (BMNH, CNC, INBio, INHS, NMNH). COSTA RICA, ACG database codes: DHJPAR0024807, DHJPAR0024910, DHJPAR0025022, DHJPAR0025055, DHJPAR0025061, DHJPAR0025075, DHJPAR0025079, DHJPAR0025083, DHJPAR0025101, DHJPAR0025109, DHJPAR0025110, DHJPAR0025118, DHJPAR0025178, DHJPAR0025317, DHJPAR0025342, DHJPAR0025355, DHJPAR0025406, DHJPAR0025449, DHJPAR0025470, DHJPAR0025521, DHJPAR0025824, DHJPAR0025826, DHJPAR0025827, DHJPAR0025830, DHJPAR0025831, DHJPAR0025840, DHJPAR0025858, DHJPAR0025860, DHJPAR0025866, DHJPAR0025910, DHJPAR0025952, DHJPAR0025959, DHJPAR0026008, DHJPAR0026033, DHJPAR0026107, DHJPAR0026206, DHJPAR0026247, DHJPAR0026268, DHJPAR0026275, DHJPAR0026287, DHJPAR0026289, DHJPAR0026326, DHJPAR0026336, DHJPAR0026390, DHJPAR0026444, DHJPAR0026454, DHJPAR0026464, DHJPAR0026485, DHJPAR0026488, DHJPAR0026497, DHJPAR0026511, DHJPAR0026514, DHJPAR0026525, DHJPAR0026526, DHJPAR0026549, DHJPAR0026556, DHJPAR0026592, DHJPAR0026623, DHJPAR0026653, DHJPAR0027668, DHJPAR0026671, DHJPAR0026672, DHJPAR0026690, DHJPAR0026703, DHJPAR0026711, DHJPAR0026722, DHJPAR0026726, DHJPAR0026731, DHJPAR0026744, DHJPAR0026745, DHJPAR0026752, DHJPAR0026769, DHJPAR0026771, DHJPAR0026783, DHJPAR0026786, DHJPAR0026796, DHJPAR0026798, DHJPAR0026806, DHJPAR0026822, DHJPAR0026835, DHJPAR0026844, DHJPAR0026861, DHJPAR0026872, DHJPAR0026878, DHJPAR0026940, DHJPAR0026943, DHJPAR0026964, DHJPAR0026972, DHJPAR0026973, DHJPAR0026981, DHJPAR0026995, DHJPAR0026998, DHJPAR0027000, DHJPAR0027036, DHJPAR0027058, DHJPAR0027076, DHJPAR0027088, DHJPAR0027093, DHJPAR0027094, DHJPAR0027107, DHJPAR0027108, DHJPAR0027128, DHJPAR0027129, DHJPAR0026150, DHJPAR0027151, DHJPAR0027162, DHJPAR0027168, DHJPAR0027176, DHJPAR0027177, DHJPAR0027180, DHJPAR0027182, DHJPAR0027183, DHJPAR0027193, DHJPAR0027203, DHJPAR0027206, DHJPAR0027209, DHJPAR0027212, DHJPAR0027229, DHJPAR0027252, DHJPAR0027255, DHJPAR0027256, DHJPAR0027302, DHJPAR0027312, DHJPAR0027330, DHJPAR0027331, DHJPAR0027346, DHJPAR0027353, DHJPAR0027355, DHJPAR0027369, DHJPAR0027375, DHJPAR0027376, DHJPAR0027385, DHJPAR0027393, DHJPAR0027400, DHJPAR0027401, DHJPAR0027403, DHJPAR0027405, DHJPAR0027407, DHJPAR0027410, DHJPAR0027419, DHJPAR0027425, DHJPAR0027432, DHJPAR0027437, DHJPAR0027440, DHJPAR0027446, DHJPAR0027450, DHJPAR0027451, DHJPAR0027452, DHJPAR0027453, DHJPAR0027455, DHJPAR0027460, DHJPAR0027534, DHJPAR0027614, DHJPAR0033744.

#### Diagnosis.

It belongs to the *annulicornis* species-group, and can be separated from other species within that group based on the combination of T2 mostly smooth and polished, antenna brown with flagellomeres 6–8 white (rarely also posterior half of flagellomere 5, white band clearly occupying less than one third of antenna length), and metasoma with T3+ partially brown. Those features are shared with the morphologically similar *Pseudapanteles luisguillermosolisi*, but *Pseudapanteles margaritapenonae* has darker areas on propodeum, metapleuron, metascutellum and axillar complex, while *luisguillermosolisi* has the mesosoma entirely orange-yellow.

#### Description.

**Female.** Body length 2.2–2.3 mm or 2.4–2.5 mm. Fore wing length 2.4–2.5 mm or 2.6–2.7 mm. Head color: mostly dark brown to black; except for orange on most of frons and face, and yellow clypeus, labrum, mandibles, and spot on lower corner of gena near oral foramen. Flagellomere color: central flagellomere white-yellow, rest dark brown to black. Mesosoma color: orange to yellow-orange, with propodeum, metascutellum and parts of axillar complex darker than rest of mesosoma; rarely anteromesoscutum with dark marks laterally and centrally on anterior 0.3. Metasoma color (dorsally): mediotergites 1–2 orange-yellow, rest of mediotergites brown. Coxae color: all pale. Metatibia color: pale. Metatarsus color: pale. Pterostigma color: entirely dark, rarely mostly dark, but with anterior pale spot. Mediotergite 1 length/width at posterior margin 4.1–4.5 × or 4.6–5.0 ×. Mediotergite 1 maximum width/width at posterior margin 2.3–2.4 × or 2.5–2.6 ×. Mediotergite 2 width at posterior margin/length: 2.9–3.1 x, 3.2–3.3 x, rarely 3.4–3.5 ×. Mediotergite 2 sculpture: Mostly smoth and polished. Ovipositor sheaths length: 0.7 × as long as metatibia.

**Male.** As female, but with all flagellomeres brown and darker body coloration (especially on anteromesoscutum and propodeum).

#### Molecular data.

Sequences in BOLD: 157, barcode compliant sequences: 152.

#### Biology/ecology.

Malaise-trapped, ACG rainforest.

#### Distribution.

Costa Rica, ACG rain forest.

#### Etymology.

This species is named in honour of Sra. Margarita Penon who listened patiently to a half hour of awkward academic description, addressed to Costa Rica’s political structure, of the ACG concept, translated it into two sentences for President-elect Oscar Arias in 1986, and thereby set the process in motion that protects all of these wasps and hundreds of thousands of other ACG species.

#### Comments.

*Pseudapanteles margaritapenonae* is rather variable morphologically, with color varying from very dark brown (including marks on anteromesoscutum) to almost lacking dark areas. The latter extreme approaches the coloration of *Pseudapanteles luisguillermosolisi*. However, these species differ as described in the diagnosis and the comments for *Pseudapanteles luisguillermosolisi*.

### Pseudapanteles mariobozai

Taxon classificationAnimaliaHymenopteraBraconidae

Fernández-Triana & Whitfield sp. n.

http://zoobank.org/F7632E9A-FDD5-406B-964F-24D7CF30CEF1

Figs 94 –98

#### Holotype.

♂ in CNC. COSTA RICA, ACG, Alajuela Province, Sector San Cristobal, Bosque Trampa Malaise, 815m, 10.86280, -85.38460, 16.vi.2007. ACG database code: DHJPAR0025932.

#### Diagnosis.

It belongs to the *annulicornis* species-group, and can be separated from other species within that group based on the relatively extensive dark area on metatibia (0.6 its length), brown pterostigma, T1 and T2 shape, and body length of at least 2.1 mm.

#### Description.

**Male.** Body length 2.2–2.3 mm. Fore wing length 2.2–2.3 mm. Head color: mostly dark brown to black, except for yellow clypeus, labrum, mandibles, and spot on lower corner of gena near oral foramen. Mesosoma color: entirely dark brown to black. Metasoma color (dorsally): entirely dark brown to black. Coxae color: pale/pale/mostly or completely dark. Metatibia color: mostly dark, with anterior 0.4 pale. Metatarsus color: dark. Pterostigma color: entirely dark.

**Female.** Unknown.

#### Molecular data.

Sequences in BOLD: 1, barcode compliant sequences: None.

#### Biology/ecology.

Malaise-trapped.

#### Distribution.

Costa Rica, ACG rain forest.

#### Etymology.

This species is named in honour of Sr. Mario Boza in recognition of his co-midwifery and constant caretaking of the Costa Rican National Park System, today the Areas Silvestres Protegidas (ASPs) of the Sistema de Areas de Conservacion (SINAC) of MINAE, as well as being the Director of Fundación Neotrópica when it received its first major donation for the ACG power line and land purchase in 1985.

### Pseudapanteles mariocarvajali

Taxon classificationAnimaliaHymenopteraBraconidae

Fernández-Triana & Whitfield sp. n.

http://zoobank.org/51166172-A491-4511-91CE-1250C24208E0

Figs 99 –104

#### Holotype.

♀ in CNC. COSTA RICA, ACG, Guanacaste Province, Sector San Cristobal, Tajo Angeles, 540m, 10.86472, -85.41531, 12.xii.2010. ACG database code: DHJPAR0041506.

#### Paratypes.

6 ♀, 4 ♂ (CNC). COSTA RICA, ACG database codes: DHJPAR0035505, DHJPAR0039022, DHJPAR0041914, DHJPAR0041975, DHJPAR0042032, DHJPAR0052339, DHJPAR0053022, DHJPAR0054758, DHJPAR0055487, DHJPAR0055525.

#### Diagnosis.

It is the only known species in the *mariocarvajali* group, and can be separated from all other known species of *Pseudapanteles* based on the shape of T2, and length of body and fore wing.

#### Description.

**Female.** Body length 3.4–3.5 mm. Fore wing length 3.4–3.5 mm. Head color: entirely yellow to orange. Flagellomere color: all flagellomere brown to black. Mesosoma color: mostly orange, with parts or all of propodeum, metapleuron, metascutellum, and axillar complex brown to black. Metasoma color (dorsally): mostly dark brown to black, except for yellow-orange anterior 0.4–0.6 of mediotergite 1. Coxae color: all pale. Metatibia color: mostly pale, with posterior 0.1–0.2 dark. Metatarsus color: dark. Pterostigma color: entirely dark. Mediotergite 1 length/width at posterior margin 3.1–3.5 ×. Mediotergite 1 maximum width/width at posterior margin 1.7–1.8 ×. Mediotergite 2 width at posterior margin/length: 1.8–1.9 ×. Mediotergite 2 sculpture: Mostly with longitudinally striate sculpture (sometimes with small, smooth area centrally). Ovipositor sheaths length: 1.2 × as long as metatibia or 1.3 × as long as metatibia.

**Male.** Much darker coloration than female, especially on metascutellum, propodeum, metacoxa and metasoma.

#### Molecular data.

Sequences in BOLD: 10, barcode compliant sequences: 10.

#### Biology/ecology.

Hosts: *Stenoma adytomes*, *Stenoma* sp. with interim name Janzen687 (Elachistidae).

#### Distribution.

Costa Rica, ACG dry forest and rain forest.

#### Etymology.

This species is named in honour of Sr. Mario Carvajal in recognition of his support, as Minister of Agriculture, for ACG biodiversity conservation through biodiversity development, and watchful support of ACG resources in the Fundacion de Parques Nacionales and in ACG.

### Pseudapanteles maureenballesteroae

Taxon classificationAnimaliaHymenopteraBraconidae

Fernández-Triana & Whitfield sp. n.

http://zoobank.org/7116A566-F7B3-40D7-8FA1-CBAF0C8820E6

Figs 105 –109

#### Holotype.

♂ in CNC. COSTA RICA, ACG, Alajuela Province, Sector San Cristobal, Estación San Gerardo, 575 m, 10.88009, -85.38887, 15.iv.2008. ACG database code: DHJPAR0026281.

#### Diagnosis.

It belongs to the *gouleti* species-group, and can be separated from other species within that group by the combination of pterostigma yellow-white, with very thin brown margins, propodeum only slightly sculptured on posterolateral corners and differentiated into elevated central area (which is shiny) and depressed posterolateral corners, and metasoma tergites dark brown except for anterior 0.6 of T1 which is yellow.

#### Description.

**Male.** Body length 2.0–2.1 mm. Fore wing length 2.2–2.3 mm. Head color: mostly dark brown to black, except for yellow clypeus, labrum, mandibles, and spot on lower corner of gena near oral foramen. Mesosoma color: entirely dark brown to black. Metasoma color (dorsally): mostly dark brown to black, except for yellow-orange anterior 0.4–0.6 of mediotergite 1. Coxae color: pale/pale/mostly or completely dark. Metatibia color: mostly pale, with posterior 0.1–0.2 dark. Metatarsus color: dark. Pterostigma color: pale, with thin dark margins.

**Female.** Unknown.

#### Molecular data.

Sequences in BOLD: 1, barcode compliant sequences: 1.

#### Biology/ecology.

Malaise-trapped.

#### Distribution.

Costa Rica, ACG rain forest.

#### Etymology.

This species is named in honour of Sra. Maureen Ballestero, Diputada from Guanacaste, and stimulator and promoter of ACG’s efforts to develop its geothermal resources as part of its quest for financial independence and conservation through non-damaging biodiversity development.

### Pseudapanteles moerens

Taxon classificationAnimaliaHymenopteraBraconidae

(Nixon, 1965) comb. n.

Figs 110 –115

Apanteles moerens Nixon, 1965: 145 (original description).

#### Holotype.

♀ in BMNH (examined). BRAZIL, Nova Teutonia, 27°11'S, 52°23'W, 16.ix.1935, Fritz Plaumann, B.M. Type HYM. 3c.1483.

#### Diagnosis.

It belongs to the *annulicornis* species-group, and can be separated from other species within that group based on head entirely yellow-orange, coloration of mesosoma and metasoma, and shape of T1 and T2.

#### Comments.

Only the holotype specimen is known. Based on examination of the fore wing venation, propodeum median carina, mediotergites, hypopygium, ovipositor and ovipositor sheaths, this species clearly belongs to *Pseudapanteles*.

### Pseudapanteles munifigueresae

Taxon classificationAnimaliaHymenopteraBraconidae

Fernández-Triana & Whitfield sp. n.

http://zoobank.org/66B99E52-662B-40BA-A20E-BE4D111172B8

Figs 116 –120

#### Holotype.

♀ in CNC. COSTA RICA, ACG, Alajuela Province, Sector San Cristobal, Rio Blanco Abajo, 500m, 10.90037, -85.37254, 23.iv.2008. ACG database code: DHJPAR0027221.

#### Diagnosis.

It belongs to the *annulicornis* species-group, and can be separated from other species within that group based on metatibia relatively extensively dark (on posterior 0.6), shape of T1 and T2, and length of body and fore wing.

#### Description.

**Female.** Body length 1.8–1.9 mm. Fore wing length 1.8–1.9 mm. Head color: mostly dark brown to black, except for yellow clypeus, labrum, mandibles, and spot on lower corner of gena near oral foramen. Flagellomere color: all flagellomere brown to black. Mesosoma color: entirely dark brown to black. Metasoma color (dorsally): mostly dark brown to black, except for yellow-orange anterior 0.4–0.6 of mediotergite 1. Coxae color: pale/pale/mostly or completely dark. Metatibia color: mostly dark, with anterior 0.4 pale. Metatarsus color: dark. Pterostigma color: entirely dark. Mediotergite 1 length/width at posterior margin 5.6–6.0 ×. Mediotergite 1 maximum width/width at posterior margin 3.5 × or more. Mediotergite 2 width at posterior margin/length: 2.7–2.8 ×. Mediotergite 2 sculpture: Mostly smoth and polished. Ovipositor sheaths length: 0.7 × as long as metatibia.

**Male.** Unknown.

#### Molecular data.

Sequences in BOLD: 1, barcode compliant sequences: 1.

#### Biology/ecology.

Malaise-trapped.

#### Distribution.

Costa Rica, ACG rain forest.

#### Etymology.

This species is named in honour of Sra. Muni Figueres in recognition of her understanding and support of ACG biodiversity development since the late 1980’s, and most recently as Costa Rica’s Ambassador to the United States, based in Washington, D.C.

### Pseudapanteles nerion

Taxon classificationAnimaliaHymenopteraBraconidae

(Nixon, 1965)

Figs 121 –125

Apanteles nerion Nixon, 1965: 142 (original description).
Pseudapanteles nerion : Mason 1981: 86 (revised combination).

#### Holotype.

♀ in BMNH (examined). BRAZIL, Nova Teutonia, 27°11'S, 52°23'W, 26.iv.1938, Fritz Plaumann, B.M. 1938-682.

#### Diagnosis.

It belongs to the *annulicornis* species-group, and can be separated from other species within that group based on metasoma almost entirely dark brown (except for laterotergites 1 and 2), T2 shape, and relatively long ovipositor sheaths (as long as metatibia).

#### Comments.

Only the holotype specimen is known.

### Pseudapanteles nigrovariatus

Taxon classificationAnimaliaHymenopteraBraconidae

(Muesebeck, 1921)

Figs 126 –130

Apanteles nigrovariatus Muesebeck, 1921: 523 (original description).
Pseudapanteles nigrovariatus : Mason 1981: 86 (revised combination).

#### Holotype.

♀ in NMNH (examined). UNITED STATES, Pennsylvania, Mount Holly Springs. USNM type No. 22522.

#### Diagnosis.

It belongs to the *annulicornis* species-group, and can be separated from other species within that group based on most of mesosoma (except for metanotum and propodeum black), metasoma and legs reddish brown.

#### Distribution.

United States (Georgia, Pennsylvania).

### Pseudapanteles oscarariasi

Taxon classificationAnimaliaHymenopteraBraconidae

Fernández-Triana & Whitfield sp. n.

http://zoobank.org/2DDD7EA1-6AFF-43D2-903C-625389624E2A

Figs 131 –135

#### Holotype.

♀ in CNC. COSTA RICA, ACG, Alajuela Province, Sector Rincon Rain Forest, Sendero Albergue Crater, 980m, 10.84886, -85.3281, 16.v.2010. ACG database code: DHJPAR0040498.

#### Paratypes.

1 ♀, 2 ♂ (CNC). COSTA RICA, ACG database codes: DHJPAR0039450, DHJPAR0043037, 10-SRNP-2415.

#### Diagnosis.

It belongs to the *gouleti* species-group, and can be separated from other species within that group based on pronotal collar yellow-orange, anteromesoscutum entirely orange-yellow, metasomal tergites entirely dark brown to black, and shape of T1.

#### Description.

**Female.** Body length 2.6–2.7 mm. Fore wing length 2.8–2.9 mm. Mesosoma color: mostly dark brown to black, with pronotum, propleura, anteromesoscutum, spot on mesopleura, and scutellar disc at least partially orange. Metasoma color (dorsally): mostly dark brown to black, except for yellow-orange anterior 0.4–0.6 of mediotergite 1. Coxae color: pale/pale/mostly or completely dark. Pterostigma color: entirely dark. Mediotergite 1 length/width at posterior margin 2.1–2.5 ×. Mediotergite 1 maximum width/width at posterior margin 1.3–1.4 ×. Mediotergite 2 width at posterior margin/length: 2.5–2.6 ×. Mediotergite 2 sculpture: Mostly with longitudinally striate sculpture (sometimes with small, smooth area centrally).

**Male.** Much darker coloration than female, especially on mesosoma and metasoma.

#### Molecular data.

Sequences in BOLD: 5, barcode compliant sequences: 5.

#### Biology/ecology.

Hosts: Elachistidae: *Antaeotricha* sp. with interim name Janzen888, and two other confamilials.

#### Distribution.

Costa Rica, ACG cloud forest and rain forest.

#### Etymology.

This species is named in honour of former President Oscar Arias who, upon listening to Margarita Penon’s summary of the ACG concept in 1986, set ACG survival policy in motion with “Sounds good to me if it doesn’t cost Costa Rica anything”; it hasn’t.

### Pseudapanteles ottonsolisi

Taxon classificationAnimaliaHymenopteraBraconidae

Fernández-Triana & Whitfield sp. n.

http://zoobank.org/2E2D90DA-24AD-4B17-935B-18B77D33B1A5

Figs 136 –140

#### Holotype.

♀ in CNC. COSTA RICA, ACG, Guanacaste Province, Sector Santa Rosa, Area Administrativa, 295m, 10.83764, -85.61871, 25.xii.2008. ACG database code: DHJPAR0031749.

#### Diagnosis.

It belongs to the *annulicornis* species-group, and can be separated from other species within that group based on head entirely yellow-orange, anteromesoscutum entirely orange, T2 mostly longitudinally striate, and mesopleuron, metapleuron, axillar complex, metascutellum and propodeum dark brown to black.

#### Description.

**Female.** Body length 2.6–2.7 mm. Fore wing length 2.8–2.9 mm. Head color: entirely yellow to orange. Flagellomere color: all flagellomere brown to black. Mesosoma color: mostly dark brown to black, with pronotum, propleura, anteromesoscutum, spot on mesopleura, and scutellar disc at least partially orange. Metasoma color (dorsally): mostly dark brown to black, with anterior 0.8 of mediotergite 1 and lateral areas on mediotergites 3–7 yellow-orange. Coxae color: pale/pale/mostly or completely dark. Metatibia color: mostly pale, with posterior 0.1–0.2 dark. Metatarsus color: dark. Pterostigma color: mostly dark, but with anterior pale spot. Mediotergite 1 length/width at posterior margin 2.1–2.5 ×. Mediotergite 1 maximum width/width at posterior margin 1.7–1.8 ×. Mediotergite 2 width at posterior margin/length: 3.8–3.9 ×. Mediotergite 2 sculpture: Mostly with longitudinally striate sculpture (sometimes with small, smooth area centrally). Ovipositor sheaths length: 0.9 × as long as metatibia.

**Male.** Unknown.

#### Molecular data.

Sequences in BOLD: 1, barcode compliant sequences: 1.

#### Biology/ecology.

Malaise-trapped.

#### Distribution.

Costa Rica, ACG dry forest.

#### Etymology.

This species is named in honour of Sr. Otton Solis in recognition of his steadfast policy support of the ACG concept throughout two decades of Costa Rican political turmoil and the foundation of the party, PAC (Partido Accion Ciudadana), of Costa Rica’s President-elect Luis Guillermo Solis (no relative).

### Pseudapanteles pedroleoni

Taxon classificationAnimaliaHymenopteraBraconidae

Fernández-Triana & Whitfield sp. n.

http://zoobank.org/EDCC38BC-B9FD-4568-AF3A-1759CDF2A233

Figs 141 –145

#### Holotype.

♀ in CNC. COSTA RICA, ACG, Alajuela Province, Sector San Cristobal, Rio Blanco Abajo, 500m, 10.90037, -85.37254, 6.v.2008. ACG database code: DHJPAR0027329.

#### Diagnosis.

It belongs to the *annulicornis* species-group, and can be separated from other species within that group based on head entirely yellow-orange, anteromesoscutum with brown marks laterally and centrally on anterior 0.3, rest of mesosoma orange, and T2 smooth.

#### Description.

**Female.** Body length 2.6–2.7 mm. Fore wing length 2.6–2.7 mm. Head color: entirely yellow to orange. Flagellomere color: all flagellomere brown to black. Mesosoma color: mostly orange to yellow-orange, but with anteromesoscutum with dark marks laterally and centrally on anterior 0.3. Metasoma color (dorsally): mostly dark brown to black, except for yellow-orange anterior 0.4–0.6 of mediotergite 1. Coxae color: all pale. Metatibia color: pale. Metatarsus color: pale. Pterostigma color: entirely dark, rarely mostly dark, but with anterior pale spot. Mediotergite 1 length/width at posterior margin 2.6–3.0 ×. Mediotergite 1 maximum width/width at posterior margin 1.9–2.0 ×. Mediotergite 2 width at posterior margin/length: 3.8–3.9 ×. Mediotergite 2 sculpture: Mostly smoth and polished. Ovipositor sheaths length: 1.0 × as long as metatibia.

**Male.** Unknown.

#### Molecular data.

Sequences in BOLD: 1, barcode compliant sequences: 1.

#### Biology/ecology.

Malaise-trapped.

#### Distribution.

Costa Rica, ACG rain forest.

#### Etymology.

This species is named in honour of Dr. Pedro Leon, fellow ACG watchdog, policy and biodiversity advisor, and analyst, and Director of the Fundacion de Parques Nacionales in its seminal role in ACG development, and companion throughout the ACG long march (Janzen 2000) and efforts to endow the entire Costa Rican national park system.

### Pseudapanteles raulsolorzanoi

Taxon classificationAnimaliaHymenopteraBraconidae

Fernández-Triana & Whitfield sp. n.

http://zoobank.org/0472BB51-E8C4-4299-B7FE-594879F7191C

Figs 146 –150

#### Holotype.

♀ in CNC. COSTA RICA, ACG, Guanacaste Province, Sector Cacao, Cerro Pedregal, 1080m, 10.92767, -85.47449, 22.xi.2008. ACG database code: DHJPAR0033842.

#### Paratypes.

1 ♀, 12 ♂ (BMNH, CNC, INBio, INHS, NMNH). COSTA RICA, ACG database codes: DHJPAR0013239, DHJPAR0013404, DHJPAR0013405, DHJPAR0013411, DHJPAR0013416, DHJPAR0013419, DHJPAR0013420, DHJPAR0013422, DHJPAR0013425, DHJPAR0013426, DHJPAR0013427, DHJPAR0013605, DHJPAR0013609.

#### Diagnosis.

It belongs to the *gouleti* species-group, and can be separated from other species within that group based on anteromesoscutum and axillar complex with some orange spots, head mostly brown-black posteriorly but orange on most of frons and face, and scape yellow, contrasting with brown flagellomeres.

#### Description.

**Female.** Body length 2.2–2.3 mm. Fore wing length 2.4–2.5 mm. Head color: mostly dark brown to black; except for orange on most of frons and face, and yellow clypeus, labrum, mandibles, and spot on lower corner of gena near oral foramen. Flagellomere color: all flagellomere brown to black. Mesosoma color: mostly dark brown to black, except for posterior 0.4 of anteromesoscutum orange. Metasoma color (dorsally): mostly dark brown to black, except for yellow-orange anterior 0.4–0.6 of mediotergite 1. Coxae color: pale/pale/mostly or completely dark. Metatibia color: pale. Metatarsus color: pale. Pterostigma color: entirely dark. Mediotergite 1 length/width at posterior margin 4.1–4.5 ×. Mediotergite 1 maximum width/width at posterior margin 1.9–2.0 ×. Mediotergite 2 width at posterior margin/length: 3.2–3.3 ×. Mediotergite 2 sculpture: Mostly with longitudinally striate sculpture (sometimes with small, smooth area centrally). Ovipositor sheaths length: 0.8 × as long as metatibia.

**Male.** As female, but with darker coloration on mesosoma, metasoma and legs.

#### Molecular data.

Sequences in BOLD: 16, barcode compliant sequences: 16.

#### Biology/ecology.

Malaise-trapped.

#### Distribution.

Costa Rica, ACG dry forest.

#### Etymology.

This species is named in honour of Sr. Raul Solorzano, an environmental Vice-Minister and steadfast supporter of the foundation and survival of ACG in the swirling waters of governmental changes and 25 years of growth from a staid small national park to a dynamic large institution (http://www.acguanacaste.ac.cr).

### Pseudapanteles renecastroi

Taxon classificationAnimaliaHymenopteraBraconidae

Fernández-Triana & Whitfield sp. n.

http://zoobank.org/47C63BCE-44E9-428F-940E-B09C1E9BE729

Figs 151 –155

#### Holotype.

♀ in CNC. COSTA RICA, ACG, Guanacaste Province, Sector Santa Rosa, Area Administrativa, 295m, 10.83764, -85.61871, 25.xii.2008. ACG database code: DHJPAR0031765.

#### Paratypes.

2 ♀ (CNC). COSTA RICA, ACG database codes: DHJPAR0013149, DHJPAR0031639.

#### Diagnosis.

It belongs to the *annulicornis* species-group, and can be separated from other species within that group based on T2 mostly smooth, propodeum with short, carina like sculpture on lateral and posterior margins in addition to median longitudinal carina, and ovipositor sheaths relatively long (1.0 × as long as metatibia).

#### Description.

**Female.** Body length 2.0–2.1 mm. Fore wing length 2.0–2.1 mm. Head color: mostly dark brown to black, except for yellow clypeus, labrum, mandibles, and spot on lower corner of gena near oral foramen. Flagellomere color: all flagellomere brown to black. Mesosoma color: entirely dark brown to black. Metasoma color (dorsally): mostly dark brown to black, except for yellow-orange anterior 0.4–0.6 of mediotergite 1. Coxae color: pale/pale/mostly or completely dark. Metatibia color: mostly pale, with posterior 0.1–0.2 dark. Metatarsus color: dark. Pterostigma color: entirely dark. Mediotergite 1 length/width at posterior margin 4.6–5.0 ×. Mediotergite 1 maximum width/width at posterior margin 2.5–2.6 ×. Mediotergite 2 width at posterior margin/length: 3.6–3.7 × or 4.0–4.1 ×. Mediotergite 2 sculpture: Mostly smoth and polished. Ovipositor sheaths length: 1.0 × as long as metatibia.

**Male.** Unknown.

#### Molecular data.

Sequences in BOLD: 2, barcode compliant sequences: 1.

#### Biology/ecology.

Malaise-trapped.

#### Distribution.

Costa Rica, ACG dry forest.

#### Etymology.

This species is named in honour of Dr. Rene Castro, a Minister of Costa Rica’s MINAE, who helped ACG and INBio in their early years of exploring conservation through non-damaging biodiversity development, promoted the development of Costa Rica’s carbon market, and tolerated the growing pains of decentralized administration of Costa Rica’s conserved wildlands.

### Pseudapanteles rodrigogamezi

Taxon classificationAnimaliaHymenopteraBraconidae

Fernández-Triana & Whitfield sp. n.

http://zoobank.org/A8733711-9881-44C3-9E07-3B508DF79FA8

Figs 156 –161

#### Holotype.

♀ in CNC. COSTA RICA, ACG, Alajuela Province, Sector Rincon Rain Forest, Estacion Caribe, 415m, 10.90187, -85.27495, 23.vi.2007. ACG database code: DHJPAR0025819.

#### Diagnosis.

It belongs to the *annulicornis* species-group, and can be separated from other species within that group based on metatibia dark brown on posterior 0.9, pterostigma yellow-white, with very thin brown margins, T1 shape, and length of body and fore wing (1.6 mm).

#### Description.

**Female.** Body length 1.6–1.7 mm. Fore wing length 1.6–1.7 mm. Head color: mostly dark brown to black; except for orange on most of frons and face, and yellow clypeus, labrum, mandibles, and spot on lower corner of gena near oral foramen. Flagellomere color: all flagellomere brown to black. Mesosoma color: entirely dark brown to black. Metasoma color (dorsally): mostly dark brown to black, except for yellow-orange anterior 0.4–0.6 of mediotergite 1. Coxae color: pale/pale/mostly or completely dark. Metatibia color: mostly dark, with anterior 0.1 pale. Metatarsus color: dark. Pterostigma color: entirely pale. Mediotergite 1 length/width at posterior margin 4.6–5.0 ×. Mediotergite 1 maximum width/width at posterior margin 2.9–3.0 ×. Mediotergite 2 width at posterior margin/length: 2.0–2.1 ×. Mediotergite 2 sculpture: Mostly smoth and polished. Ovipositor sheaths length: 0.6 × as long as metatibia.

**Male.** Unknown.

#### Molecular data.

Sequences in BOLD: 1, barcode compliant sequences: 1.

#### Biology/ecology.

Malaise-trapped.

#### Distribution.

Costa Rica, ACG rain forest.

#### Etymology.

This species is named in honour of Dr. Rodrigo Gamez in recognition of his founding and developing INBio, guiding the emergence of Costa Rican national conservation policy for non-damaging use, and being the primary supporter of ACG’s early efforts to establish its endowment-supported budgetary system.

### Pseudapanteles rosemarykarpinskiae

Taxon classificationAnimaliaHymenopteraBraconidae

Fernández-Triana & Whitfield sp. n.

http://zoobank.org/C676C9E8-EF9B-4833-983A-B2C7C918607F

Figs 162 –165

#### Holotype.

♀ in CNC. COSTA RICA, ACG, Guancaste Province, Sector Santa Rosa, Bosque San Emilio, 300m, 10.84389, -85.61384, 2.vi.2007. ACG database code: DHJPAR0013244.

#### Paratypes.

15 ♀ (BMNH, CNC, INBio, INHS, NMNH). COSTA RICA, ACG database codes: DHJPAR0013246, DHJPAR0024750, DHJPAR0031583, DHJPAR0031599, DHJPAR0031644, DHJPAR0031648, DHJPAR0031654, DHJPAR0031715, DHJPAR0031746, DHJPAR0031748, DHJPAR0031754, DHJPAR0031817, DHJPAR0031833, DHJPAR0031835, DHJPAR0031836.

#### Diagnosis.

It belongs to the *gouleti* species-group, and can be separated from other species within that group based on anteromesoscutum, axillar complex and head (except for clypeus, labrum and mandibles) entirely dark brown to black, and scape brown, same color as flagellomeres.

#### Description.

**Female.** Body length 2.2–2.3 mm. Fore wing length 2.0–2.1 mm or 2.2–2.3 mm. Head color: mostly dark brown to black, except for yellow clypeus, labrum, mandibles, and spot on lower corner of gena near oral foramen. Flagellomere color: all flagellomere brown to black. Mesosoma color: entirely dark brown to black. Metasoma color (dorsally): entirely dark brown to black. Coxae color: pale/pale/mostly or completely dark. Metatibia color: pale. Metatarsus color: pale. Pterostigma color: entirely dark. Mediotergite 1 length/width at posterior margin 3.6–4.0 ×. Mediotergite 1 maximum width/width at posterior margin 1.9–2.0 ×. Mediotergite 2 width at posterior margin/length: 2.9–3.1 × or 3.8–3.9 ×. Mediotergite 2 sculpture: Mostly with longitudinally striate sculpture (sometimes with small, smooth area centrally). Ovipositor sheaths length: 1.0 × as long as metatibia or 1.1 × as long as metatibia.

**Male.** Unknown.

#### Molecular data.

Sequences in BOLD: 41, barcode compliant sequences: 39.

#### Biology/ecology.

Malaise-trapped.

#### Distribution.

Costa Rica, ACG dry forest.

#### Etymology.

This species is named in honour of Sra. Rosemary Karpinski’s teamwork with Dr. Rodrigo Gamez to obtain the political approval that allowed for the germination and growth of ACG.

### Pseudapanteles ruficollis

Taxon classificationAnimaliaHymenopteraBraconidae

(Cameron, 1911)

Figs 166 –168

Xanthomicrogaster ruficollis Cameron, 1911: 325 (original description).
Apanteles ruficollis : Wilkinson 1930: 281 (revised combination).
Pseudapanteles ruficollis : Mason 1981: 86 (revised combination).

#### Lectotype.

**Figures 166–168.**
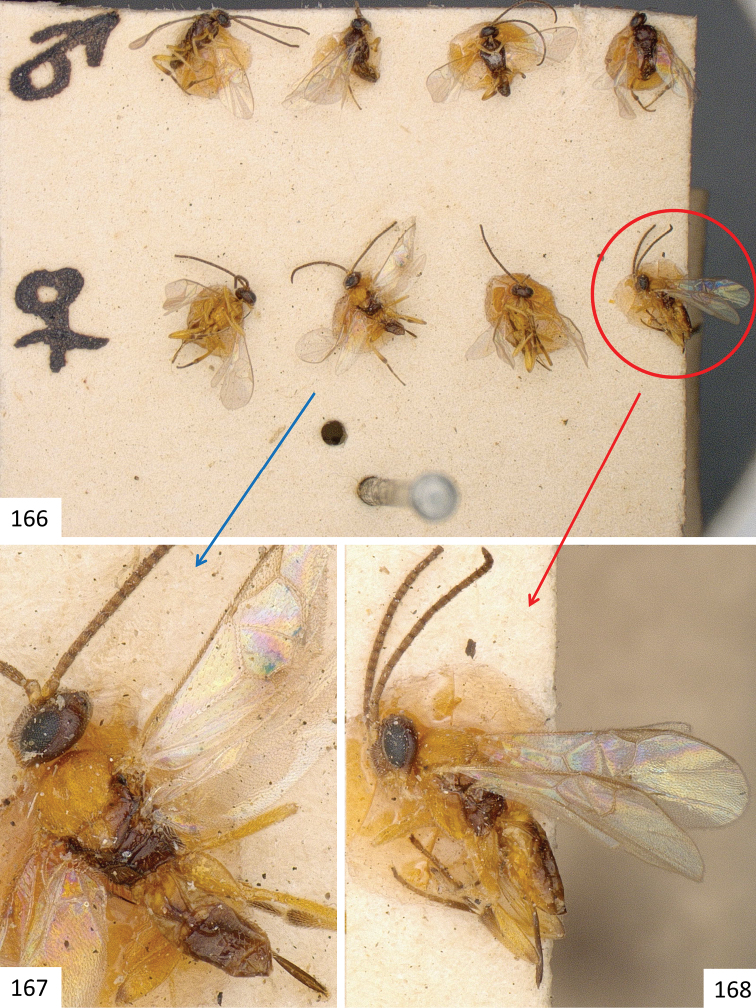
*Pseudapanteles ruficollis*. **166** Details of the card with the lectotype (shown with a red circle superimposed to the figure) and paralectotypes **167** Female paralectotype, habitus, dorsal view **168** Female lectotype, habitus, lateral view.

♀ in BMNH (examined). GUYANA, no other locality or date information. Here we designate, to increase nomenclatural stability, a lectotype from the syntype series (which includes 4 ♀ and 4 ♂ all glued on the same card, with voucher code: “B.M. Type HYM. 3.c.985”). The lectotype is the female positioned at the lower row, left corner of the card (Figs 166, 168). The paralectotypes are conspecific with the lectotype.

#### Other material examined.

24 ♀, 11 ♂ (CNC, NMNH). COSTA RICA, ACG, Alajuela Province, Sector Rincon Rain Forest, Estacion Llanura, 135m, 10.93332, -85.25331. ACG database codes: DHJPAR0026267, DHJPAR0047117, DHJPAR0047133, DHJPAR0052908, DHJPAR0053737, DHJPAR0053754, DHJPAR0053787, 11-SRNP-76958, 13-SRNP-76587.

#### Diagnosis.

It belongs to the *annulicornis* species-group, and can be separated from other species within that group based on a combination of all flagellomeres brown, T2 light brown, metatibia yellow with posterior 0.1–0.2 dark brown to black, metatarsus dark brown to black, pterostigma pale with thin brown margins, and ovipositor sheaths 0.9 × as long as metatibia.

#### Description.

**Female.** Body length 2.4–2.5 mm or 2.6–2.7 mm. Fore wing length 2.6–2.7 mm. Head color: mostly dark brown to black; except for orange on most of frons and face, and yellow clypeus, labrum, mandibles, and spot on lower corner of gena near oral foramen. Flagellomere color: all flagellomere brown to black. Mesosoma color: mostly orange, with parts or all of propodeum, metapleuron, metascutellum, and axillar complex brown to black. Metasoma color (dorsally): mostly dark brown to black, except for yellow-orange anterior 0.4–0.6 of mediotergite 1. Coxae color: all pale. Metatibia color: mostly pale, with posterior 0.1–0.2 dark. Metatarsus color: dark. Pterostigma color: pale, with thin dark margins. Mediotergite 1 length/width at posterior margin 3.6–4.0 x, rarely 3.1–3.5 × or 4.1–4.5 ×. Mediotergite 1 maximum width/width at posterior margin 2.5–2.6 ×, 2.7–2.8 ×, rarely 2.9–3.0 ×. Mediotergite 2 width at posterior margin/length: 3.8–3.9 × or 4.0–4.1 ×. Mediotergite 2 sculpture: Mostly smoth and polished. Ovipositor sheaths length: 0.9 × as long as metatibia.

**Male.** Much darker coloration than female, especially on anteromesoscutum, propodeum, metacoxa, and metasoma.

#### Molecular data.

Sequences in BOLD: 5, barcode compliant sequences: 5.

#### Biology/ecology.

Hosts: *Desmia ufeus*, *Desmia* spp. with interim names Janzen18 and Janzen19; *Spoladea recurvalis* (Crambidae).

#### Distribution.

Cuba, Costa Rica (ACG), Guyana.

#### Comments.

The syntype series (from Guyana) is morphologically indistinguishable from the ACG specimens, so we treat them as conspecific. The description provided above is based on ACG specimens. All specimens of *Pseudapanteles ruficollis* in ACG have been reared from three species of *Desmia* caterpillars feeding on Vitaceae. In ACG, this wasp has not been reared from *Spoladea recurvalis* (Crambidae), the host reported for its rearing in Guyana, despite more than 190 rearing records for ACG *Spoladea recurvalis* (that produced no microgastrine braconids). This moth is host-specific to Amaranthaceae herbs, and no *Desmia* have been reared from any of the thousands of caterpillar rearing records from ACG Amaranthaceae. However, adult *Spoladea* are black with white spots, as are *Desmia*, and are frequently misidentified as “a species of *Desmia*”. It is very likely that the Guyana record of *Pseudapanteles ruficollis* is actually a rearing from a batch of *Desmia* caterpillars, since the caterpillar that produced the wasps obviously could not be directly identified from an adult.

### Pseudapanteles sesiae

Taxon classificationAnimaliaHymenopteraBraconidae

(Viereck, 1912)

Figs 169 –173

Apanteles (Pseudapanteles) sesiae Viereck, 1912: 146 (original description).
Pseudapanteles sesiae : Mason 1981: 86 (revised combination).

#### Holotype.

♀ in NMNH (examined). UNITED STATES, Virginia, Vienna. USNM type No. 14324.

#### Other material examined.

5 ♂ (CNC). Canada, Ontario, Niagara Falls; United States, Florida, Fort Ogden; Virgina, Falls Church; Virginia, Vienna.

#### Diagnosis.

It belongs to the *annulicornis* species-group, and can be separated from other species within that group based on head, flagellomeres, mesosoma and metasoma dark brown to black, and body and fore wing length usually 3.0 mm.

#### Molecular data.

Sequences in BOLD: 2, barcode compliant sequences: none.

#### Biology/ecology.

Host: *Synanthedon scitula* (Sesiidae).

#### Distribution.

Canada (Ontario), United States (District of Columbia, Florida, Indiana, New Jersey, Texas, Virginia).

#### Comments.

Two partial DNA barcodes (100 and 164 base pairs respectively) were obtained from specimens from Canada (Ontario) and United States (Florida).

### Pseudapanteles soniapicadoae

Taxon classificationAnimaliaHymenopteraBraconidae

Fernández-Triana & Whitfield sp. n.

http://zoobank.org/908ACA74-A905-4BBA-95F5-C1B02F788E23

Figs 174 –179

#### Holotype.

♀ in CNC. COSTA RICA, ACG, Guanacaste Province, Sector Santa Rosa, Bosque Humedo, 290m, 10.85145, -85.60801, 23.ii.1998. ACG database code: DHJPAR0013238.

#### Diagnosis.

It belongs to the *gouleti* species-group, and can be separated from other species within that group by the combination of anteromesoscutum mostly dark brown to black, propodeum mostly smooth, with well-defined median carina and few short rugosities transverse to that carina, and antenna relatively shorter on its anterior half (with second flagellomere 2.5 × as long as wide, and eight flagellomere 2.2 × as long as wide).

#### Description.

**Female.** Body length 1.8–1.9 mm. Fore wing length 2.0–2.1 mm. Head color: mostly dark brown to black; except for orange on most of frons and face, and yellow clypeus, labrum, mandibles, and spot on lower corner of gena near oral foramen. Flagellomere color: all flagellomere brown to black. Mesosoma color: mostly dark brown to black, with pronotum, propleura, anteromesoscutum, spot on mesopleura, and scutellar disc at least partially orange. Metasoma color (dorsally): entirely dark brown to black. Coxae color: pale/pale/mostly or completely dark. Metatibia color: mostly pale, with posterior 0.1–0.2 dark. Metatarsus color: dark. Pterostigma color: mostly dark, but with anterior pale spot. Mediotergite 1 length/width at posterior margin 3.1–3.5 ×. Mediotergite 1 maximum width/width at posterior margin 1.9–2.0 ×. Mediotergite 2 width at posterior margin/length: 3.6–3.7 ×. Mediotergite 2 sculpture: Mostly with longitudinally striate sculpture (sometimes with small, smooth area centrally). Ovipositor sheaths length: 1.0 × as long as metatibia.

**Male.** Unknown.

#### Molecular data.

Sequences in BOLD: 1, barcode compliant sequences: 1.

#### Biology/ecology.

Malaise-trapped.

#### Distribution.

Costa Rica, ACG dry forest.

#### Etymology.

This species is named in honour of Sra. Sonia Picado in recognition of her wise counsel in advising the legal-administrative process of the expropriation of Sector Santa Elena for ACG, while she was Costa Rica’s Ambassador to the United States.

### Pseudapanteles teofilodelatorrei

Taxon classificationAnimaliaHymenopteraBraconidae

Fernández-Triana & Whitfield sp. n.

http://zoobank.org/E8035231-A2F2-4AC6-B22E-E8229B471E72

Figs 180 –185

#### Holotype.

♀ in CNC. COSTA RICA, ACG, Guanacaste Province, Sector El Hacha, Quebrada La Leona, 255m, 11.03028, -85.54781, 3.v.2011. ACG database code: DHJPAR0048162.

#### Paratype.

1 ♀ (CNC). COSTA RICA, ACG database codes: DHJPAR0045346.

#### Diagnosis.

It belongs to the *annulicornis* species-group, and can be separated from other species within that group based on metacoxa dark brown to black, ovipositor sheaths at least 1.0 × as long as metatibia, length of body and fore wing over 2.8 mm, and shape of T1 and T2.

#### Description.

**Female.** Body length 2.8–2.9 mm. Fore wing length 3.0–3.1 mm. Head color: mostly dark brown to black; except for orange on most of frons and face, and yellow clypeus, labrum, mandibles, and spot on lower corner of gena near oral foramen. Flagellomere color: all flagellomere brown to black. Mesosoma color: mostly dark brown to black, with pronotum, propleura, anteromesoscutum, spot on mesopleura, and scutellar disc at least partially orange. Metasoma color (dorsally): mediotergites 1–2 orange-yellow, rest of mediotergites brown. Coxae color: pale/pale/mostly or completely dark. Metatibia color: mostly pale, with posterior 0.1–0.2 dark. Metatarsus color: dark. Pterostigma color: mostly dark, but with anterior pale spot. Mediotergite 1 length/width at posterior margin 1.6–2.0 ×. Mediotergite 1 maximum width/width at posterior margin 1.5–1.6 ×. Mediotergite 2 width at posterior margin/length: 4.4–4.5 ×. Mediotergite 2 sculpture: Mostly smoth and polished. Ovipositor sheaths length: 1.0 × as long as metatibia.

**Male.** Unknown.

#### Molecular data.

Sequences in BOLD: 3, barcode compliant sequences: 3.

#### Biology/ecology.

Hosts: Undetermined Gelechiidae with interim name gelJanzen01 Janzen830.

#### Distribution.

Costa Rica, ACG dry forest.

#### Etymology.

This species is named in honour of Dr. Teofilo de la Torre in recognition of his many years of guidance of ICE (Instituto Nacional de Electricidad), the National Electricity Institute, and his acceptance of ICE partnerships with ACG in biodiversity development.

## Supplementary Material

XML Treatment for Pseudapanteles abantidas

XML Treatment for Pseudapanteles alfiopivai

XML Treatment for Pseudapanteles alvaroumanai

XML Treatment for Pseudapanteles analorenaguevarae

XML Treatment for Pseudapanteles annulicornis

XML Treatment for Pseudapanteles brunneus

XML Treatment for Pseudapanteles carlosespinachi

XML Treatment for Pseudapanteles carlosrodriguezi

XML Treatment for Pseudapanteles christianafigueresae

XML Treatment for Pseudapanteles dignus

XML Treatment for Pseudapanteles gouleti

XML Treatment for Pseudapanteles hernanbravoi

XML Treatment for Pseudapanteles jorgerodriguezi

XML Treatment for Pseudapanteles josefigueresi

XML Treatment for Pseudapanteles laurachinchillae

XML Treatment for Pseudapanteles lipomeringis

XML Treatment for Pseudapanteles luisguillermosolisi

XML Treatment for Pseudapanteles margaritapenonae

XML Treatment for Pseudapanteles mariobozai

XML Treatment for Pseudapanteles mariocarvajali

XML Treatment for Pseudapanteles maureenballesteroae

XML Treatment for Pseudapanteles moerens

XML Treatment for Pseudapanteles munifigueresae

XML Treatment for Pseudapanteles nerion

XML Treatment for Pseudapanteles nigrovariatus

XML Treatment for Pseudapanteles oscarariasi

XML Treatment for Pseudapanteles ottonsolisi

XML Treatment for Pseudapanteles pedroleoni

XML Treatment for Pseudapanteles raulsolorzanoi

XML Treatment for Pseudapanteles renecastroi

XML Treatment for Pseudapanteles rodrigogamezi

XML Treatment for Pseudapanteles rosemarykarpinskiae

XML Treatment for Pseudapanteles ruficollis

XML Treatment for Pseudapanteles sesiae

XML Treatment for Pseudapanteles soniapicadoae

XML Treatment for Pseudapanteles teofilodelatorrei

## Figures and Tables

**Figures 3–8.**
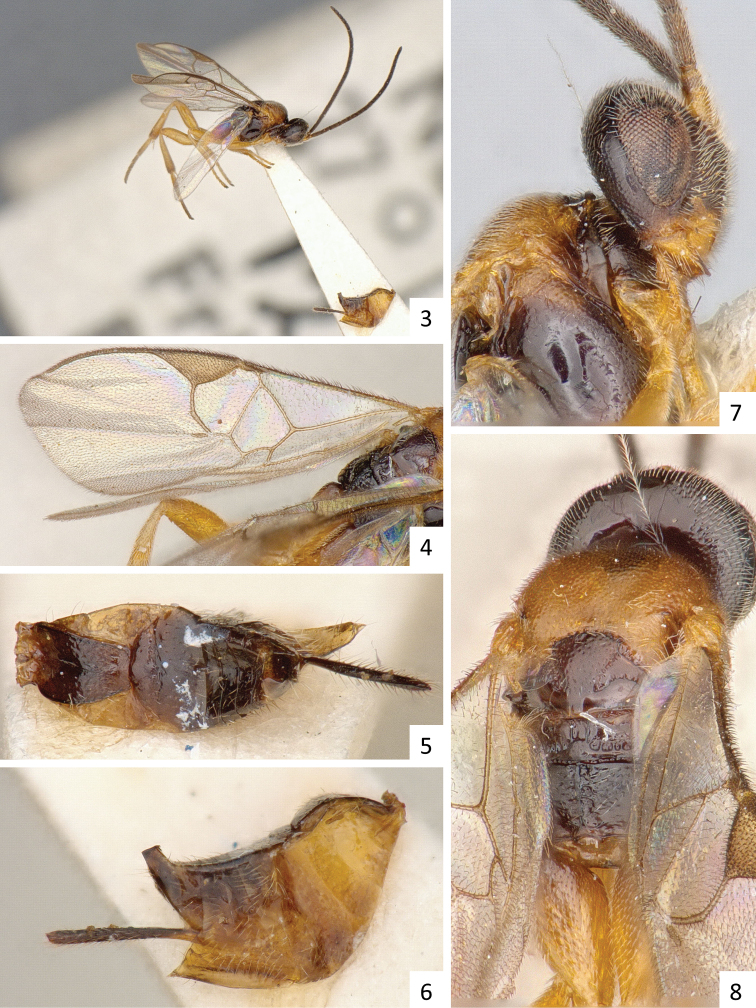
*Pseudapanteles abantidas*. **3** Habitus, lateral view **4** Fore wing **5** Metasoma, dorsal view **6** Metasoma, lateral view **7** Head and mesosoma (partially) lateral view **8** Mesosoma, dorsal view.

**Figures 9–13.**
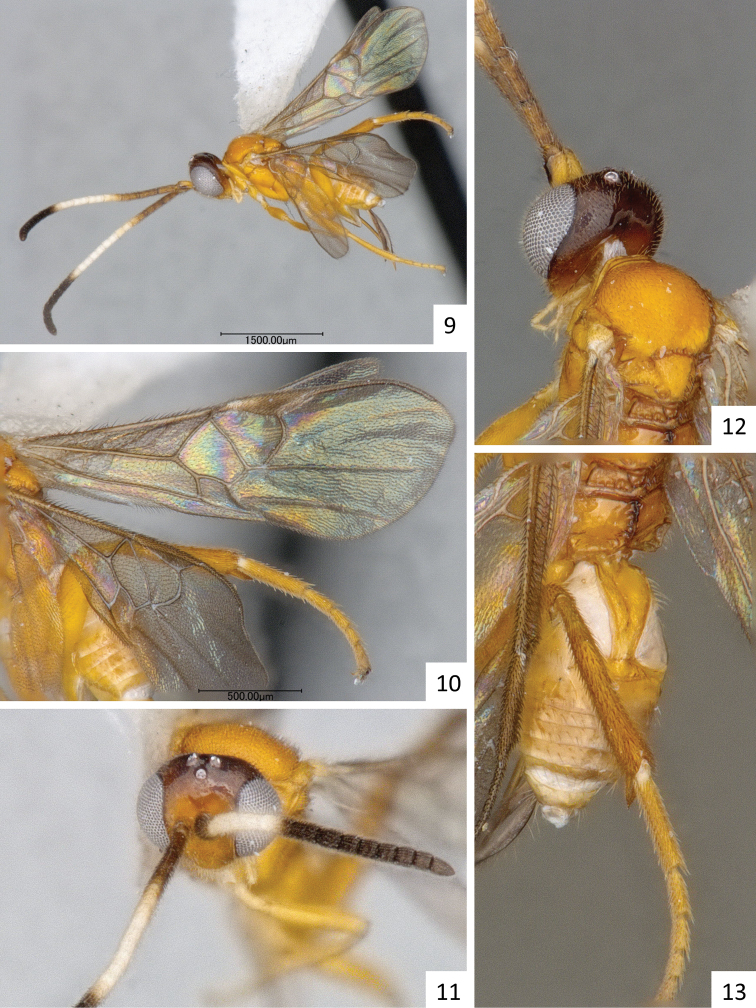
*Pseudapanteles alfiopivai*. **9** Habitus, lateral view **10** Fore wing **11** Head, frontal view **12** Head and mesosoma (partially) dorso-lateral view **13** Propodeum and metasoma, lateral view.

**Figures 14–18.**
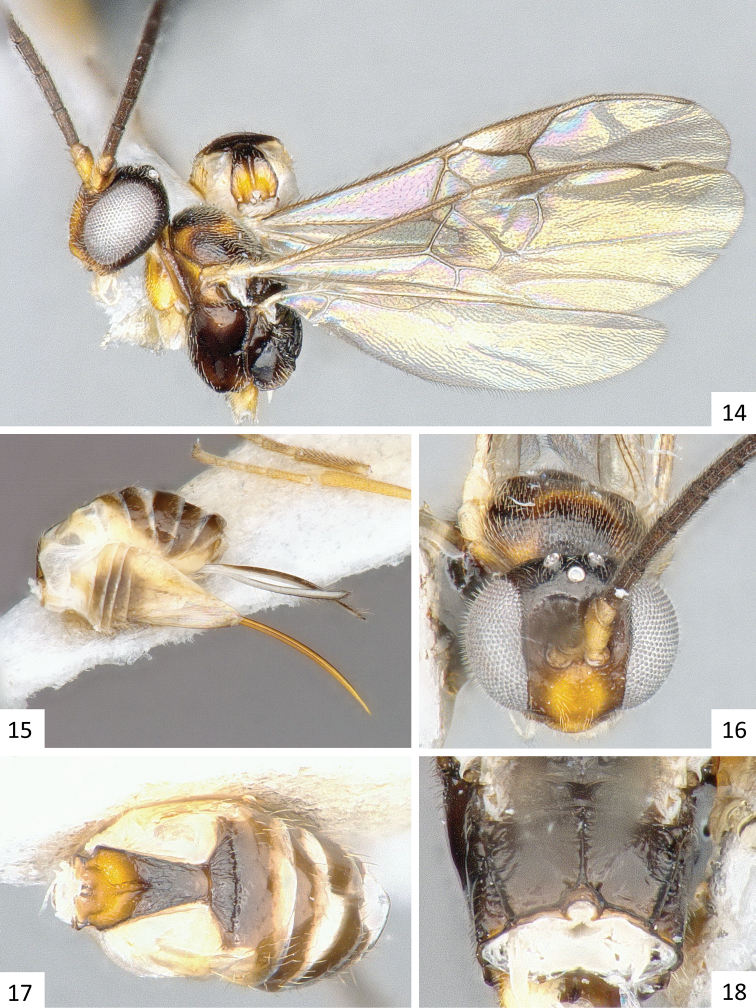
*Pseudapanteles alvaroumanai*. **14** Head, mesosoma and wings, lateral view (with partial metasoma visible in the background) **15** Metasoma and hind leg (partially), lateral view **16** Head, frontal view **17** Metasoma, dorsal view **18** Propodeum.

**Figures 19–23.**
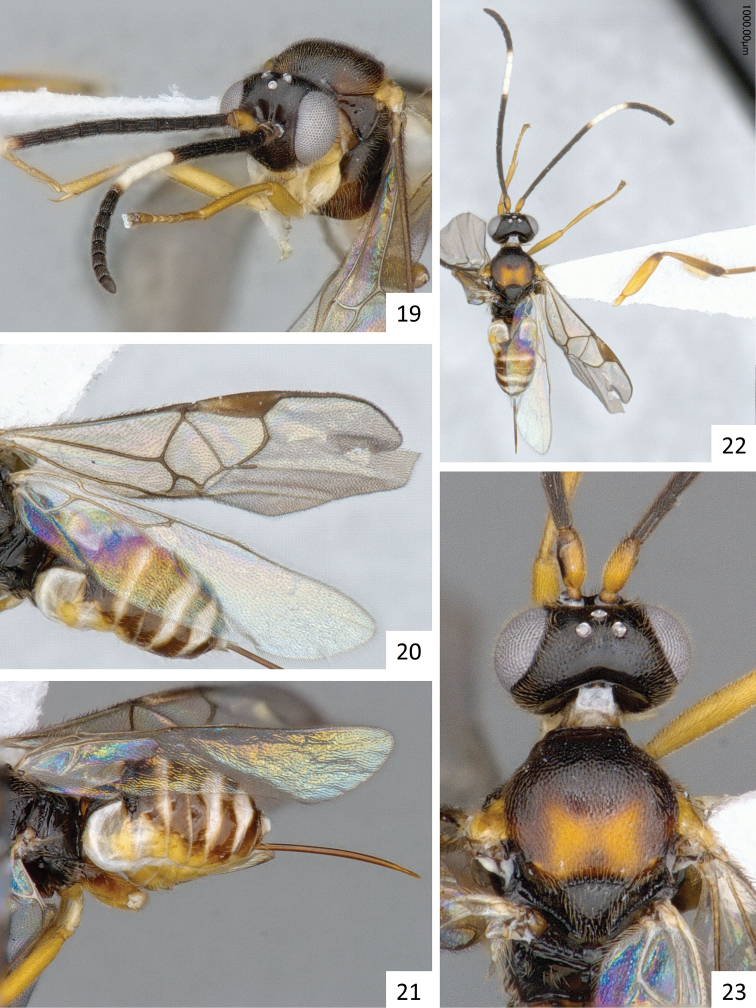
*Pseudapanteles analorenaguevarae*. **19** Head and mesosoma (partially), dorso-lateral view **20** Fore wing **21** Metasoma, lateral view **22** Habitus, dorsal view **23** Head and mesosoma, dorsal view.

**Figures 24–31.**
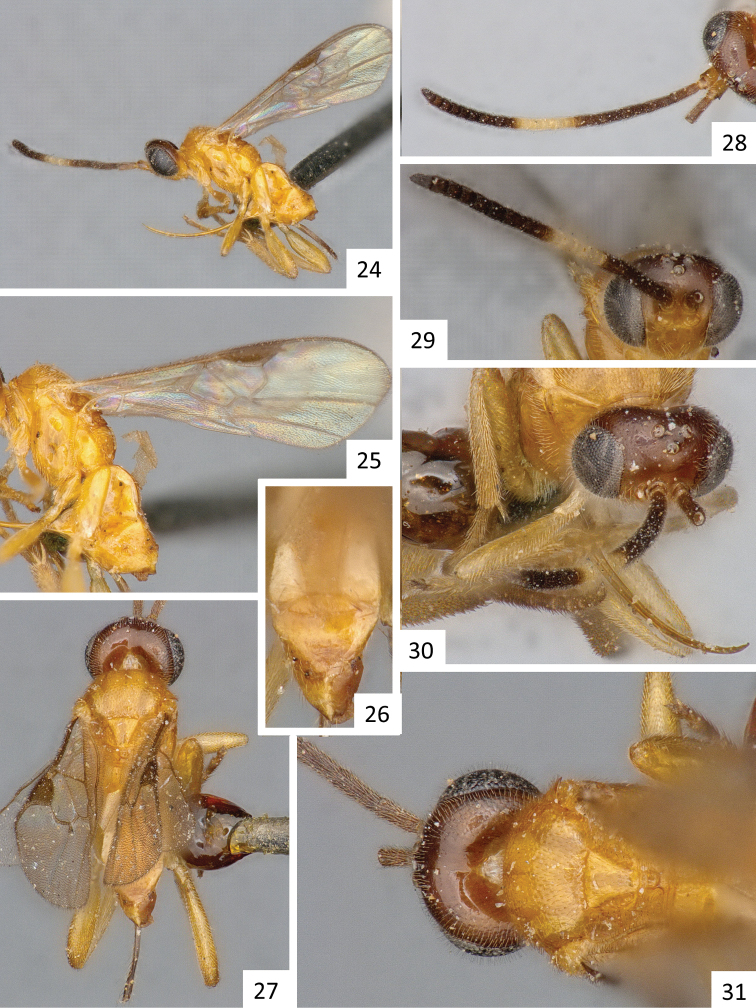
*Pseudapanteles annulicornis*. **24** Habitus, lateral view **25** Fore wing. **26** Metasoma (partially), dorsal view (picture blurriness caused by wings) **27** Habitus, dorsal view **28** Antenna **29** Head, frontal view **30** Head (frontal), and metasoma (ventrally) showing hypopygium and ovipositor **31** Head and mesosoma (partially), dorsal view.

**Figures 32–36.**
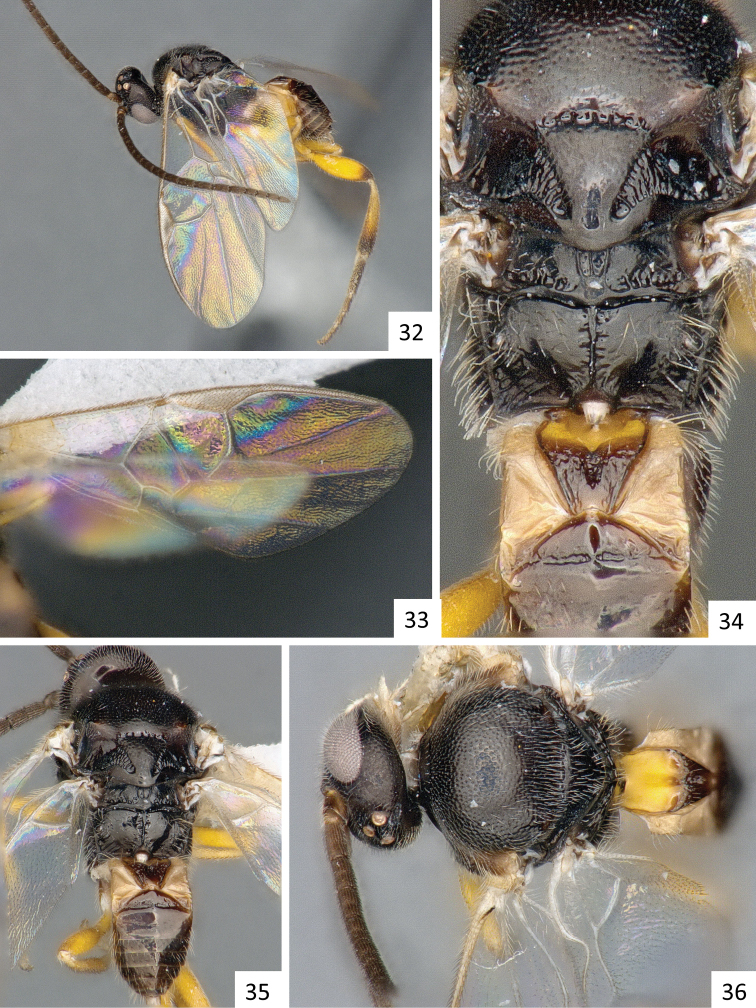
*Pseudapanteles carlosespinachi*. **32** Habitus, lateral view **33** Fore wing **34** Mesosoma and metasoma (partially), dorsal view **35** Habitus, dorsal view **36** Detail of anteromesoscutum and mediotergite 1, dorsal view.

**Figures 37–41.**
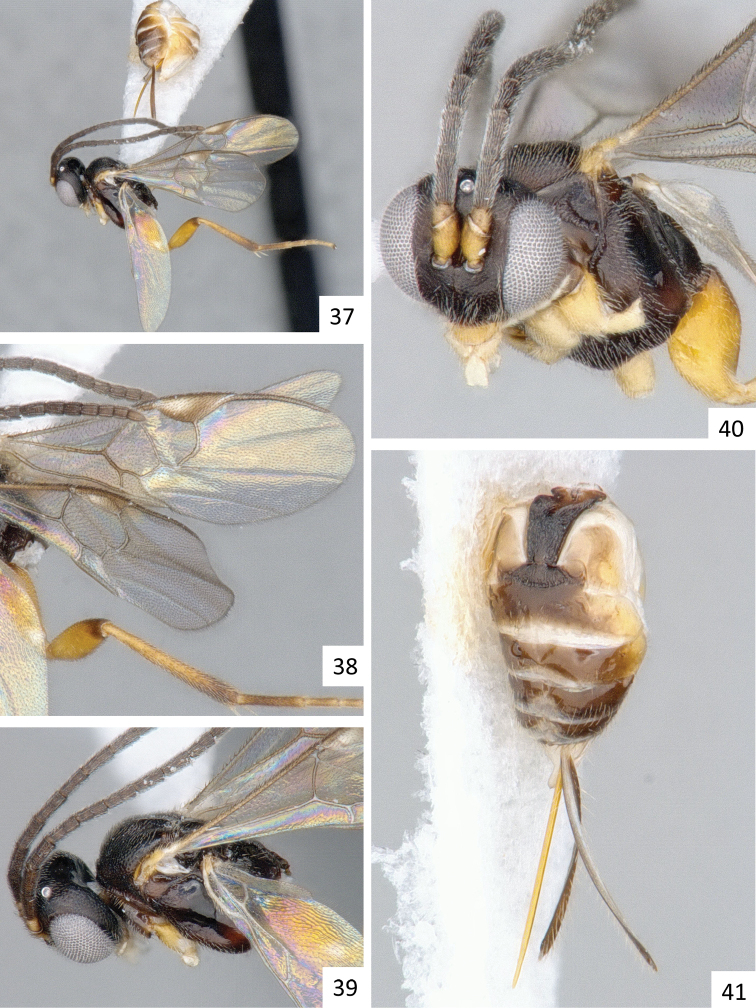
*Pseudapanteles carlosrodriguezi*. **37** Habitus, lateral view **38** Fore wing **39** Head and mesosoma, lateral view **40** Head and mesosoma, frontal view **41** Metasoma, dorsal view.

**Figures 42–47.**
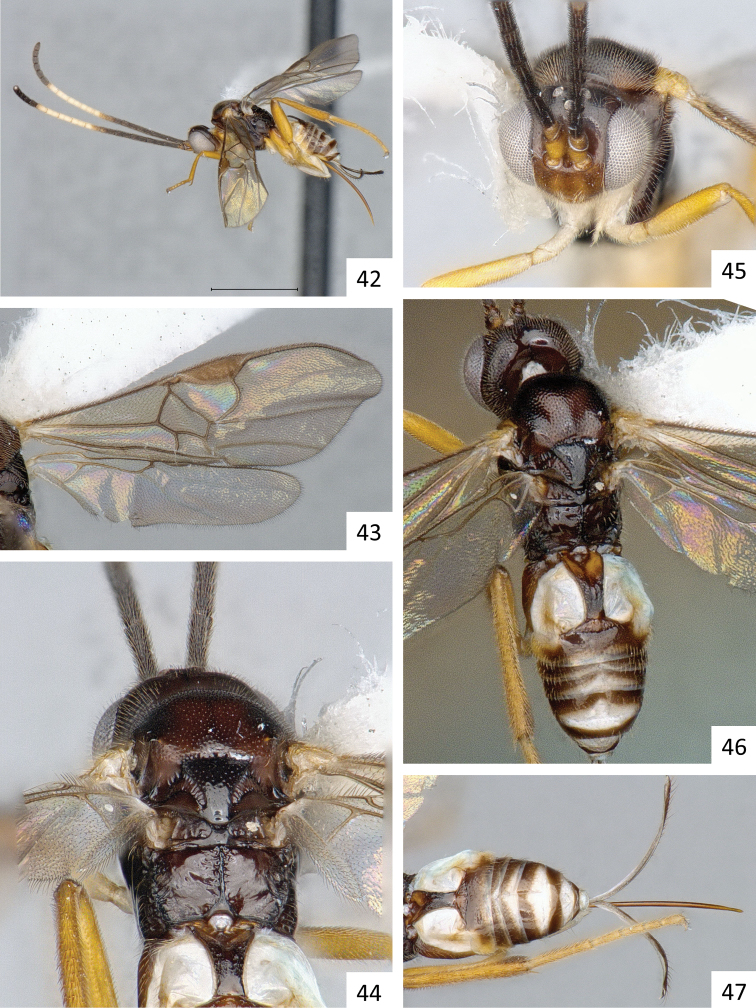
*Pseudapanteles christianafigueresae*. **42** Habitus, lateral view **43** Wings **44** Mesosoma and mediotergites 1 and 2, dorsal view **45** Head, frontal view **46** Habitus, dorsal view **47** Metasoma, dorsal view.

**Figures 48–52.**
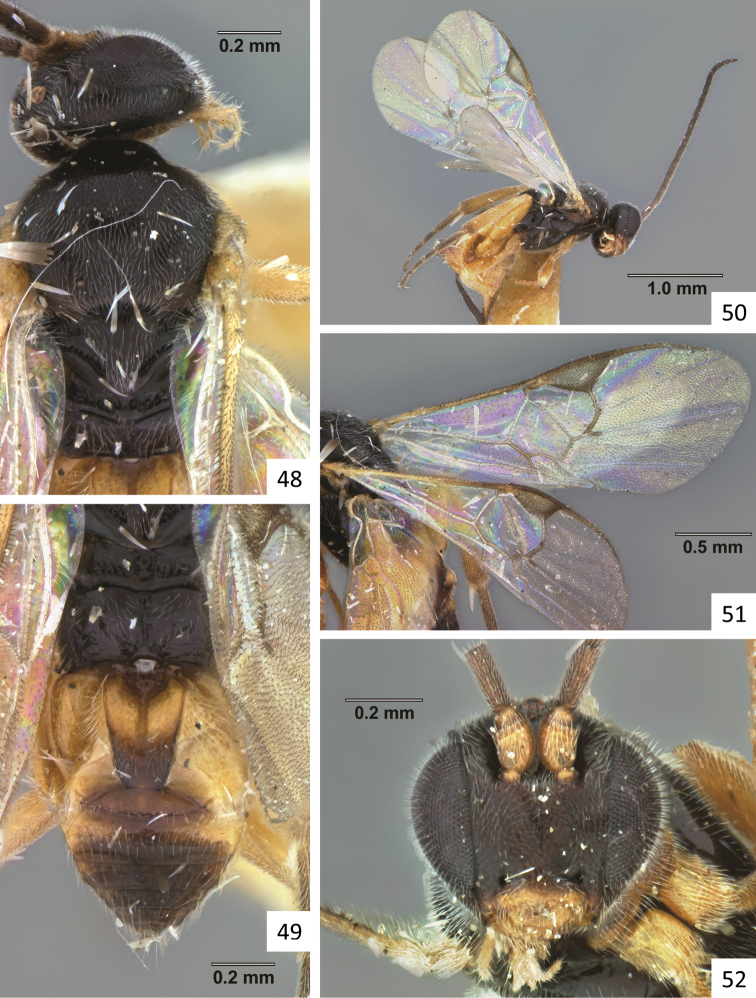
*Pseudapanteles dignus*. **48** Head and mesosoma, dorsal view **49** Propodeum and metasoma, dorsal view **50** Habitus, lateral view **51** Fore wing **52** Head, frontal view.

**Figures 53–58.**
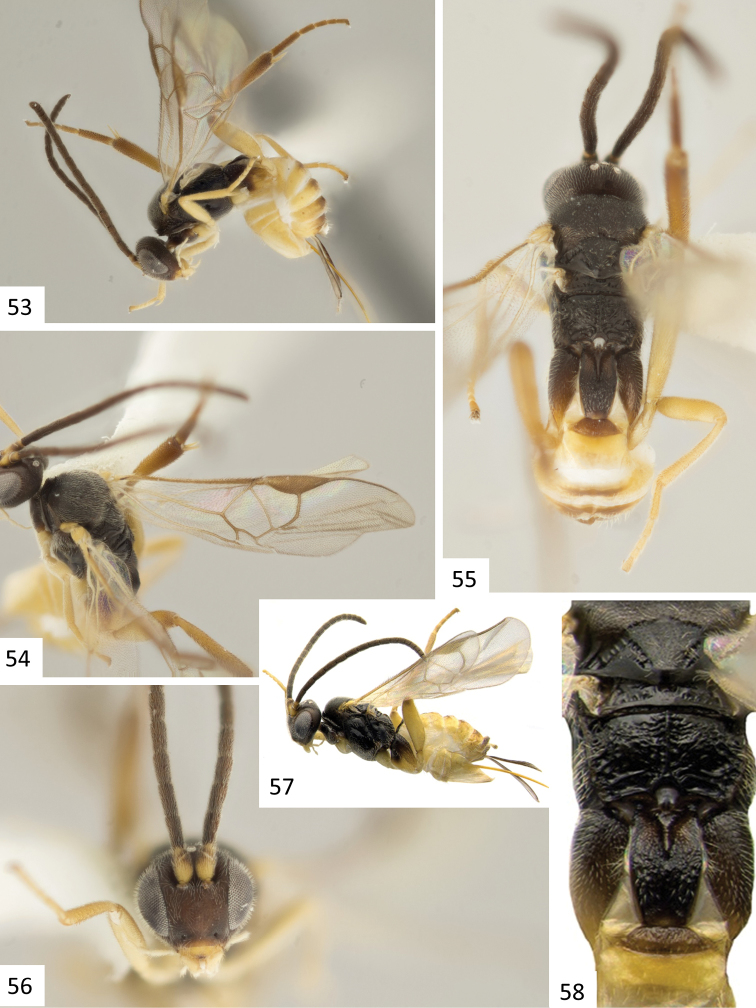
*Pseudapanteles gouleti*. Holotype specimen (**53–58**) and paratype specimen (**57–58**) **53** Habitus, lateral view **54** Wings, lateral view and metasoma (partially), dorso-lateral view **55** Habitus, dorsal view **56** Head, frontal view **57** Habitus, lateral view **58** Mesosoma and metasoma (partially), dorsal view.

**Figures 59–64.**
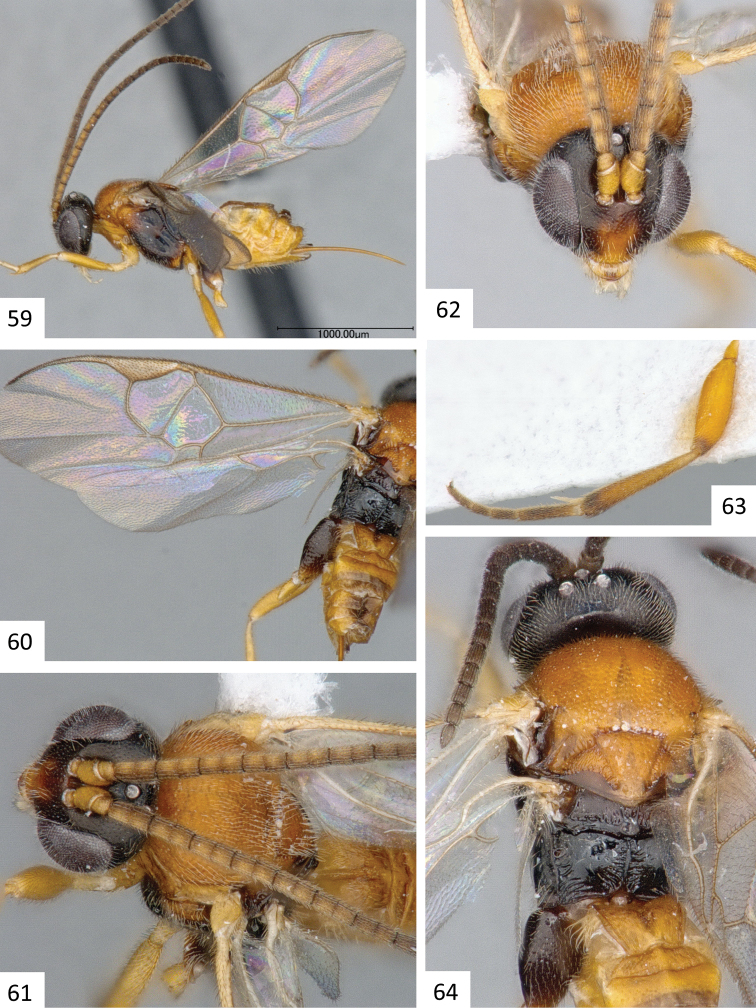
*Pseudapanteles hernanbravoi*. **59** Habitus, lateral view **60** Wings. **61** Head, mesosoma and mediotergite 1, dorsal view **62** Head, frontal view **63** Hind leg (except for coxa) **64** Habitus (partially), dorsal view.

**Figures 65–69.**
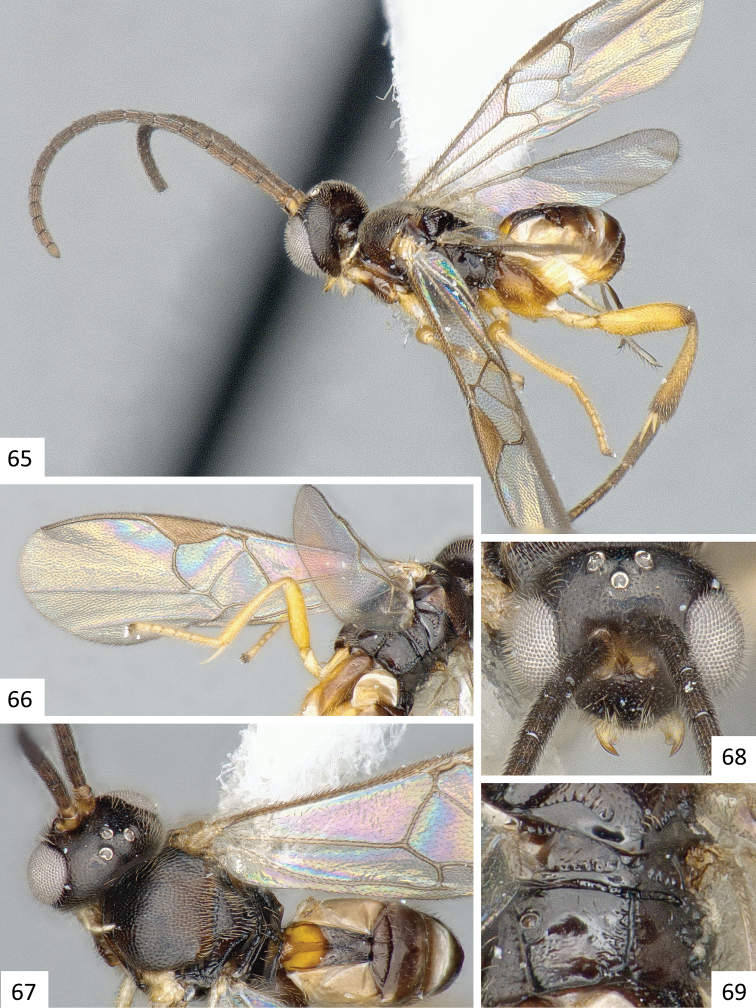
*Pseudapanteles jorgerodriguezi*. **65** Habitus, lateral view **66** Wings **67** Habitus, dorsal view **68** Head, frontal view **69** Propodeum.

**Figures 70–73.**
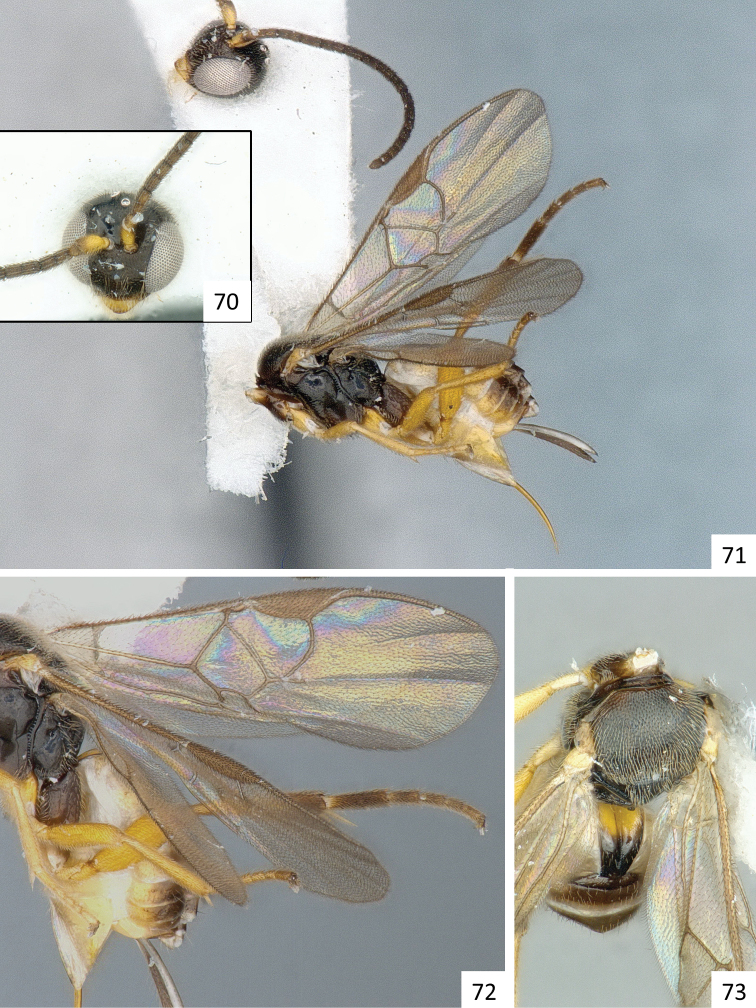
*Pseudapanteles josefigueresi*. **70** Head, frontal view **71** Habitus, lateral view **72** Wings **73** Habitus, dorsal view.

**Figures 74–78.**
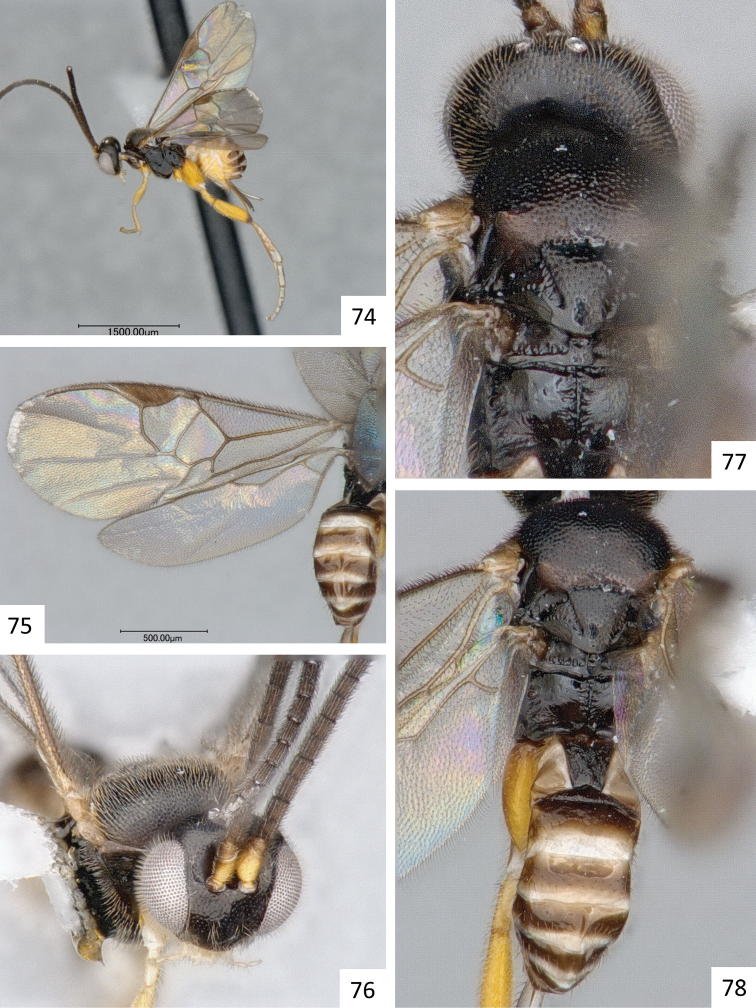
*Pseudapanteles laurachinchillae*. **74** Habitus, lateral view **75** Wings **76** Head, frontal view **77** Head and mesosoma, dorsal view **78** Mesosoma and metasoma, dorsal view.

**Figures 79–83.**
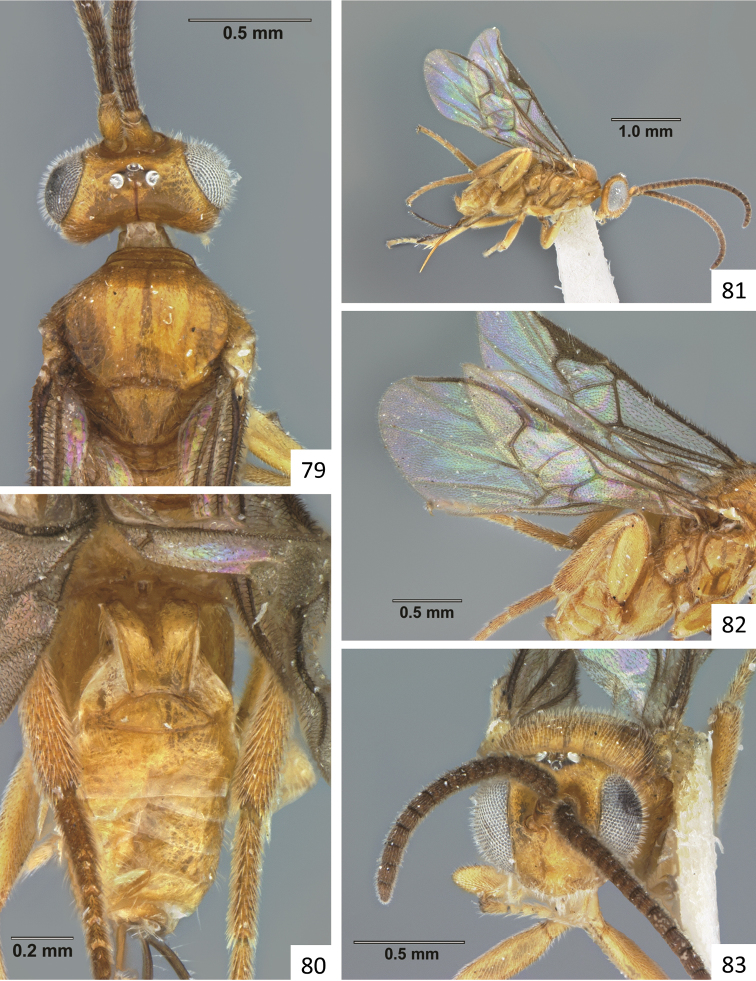
*Pseudapanteles lipomeringis*. **79** Head and mesosoma (partially), dorsal view **80** Propodeum and metasoma, dorsal view **81** Habitus, lateral view **82** Wings **83** Head, frontal view.

**Figures 84–88.**
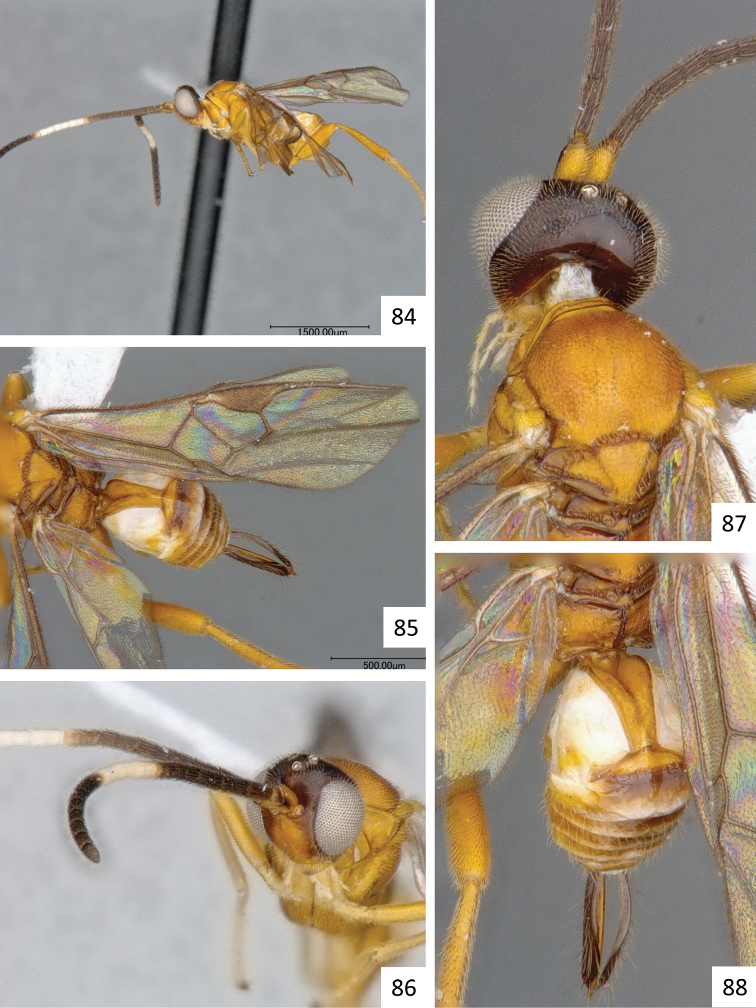
*Pseudapanteles luisguillermosolisi*. **84** Habitus, lateral view **85** Wings **86** Head, frontal view **87** Head and mesosoma (partially), dorsal view **88** Propodeum and metasoma, dorsal view.

**Figures 89–93.**
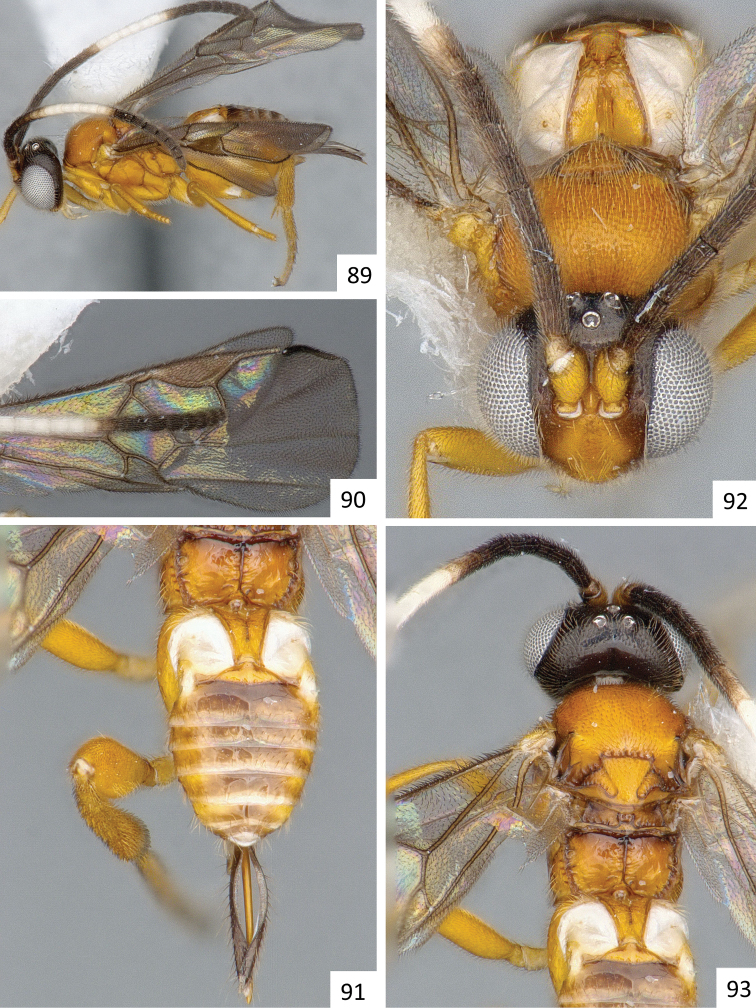
*Pseudapanteles margaritapenonae*. **89** Habitus, lateral view **90** Fore wing **91** Propodeum and metasoma, dorsal view **92** Head, frontal view (partial mesosoma and metasoma in the background) **93** Head, mesosoma and mediotergites 1–4, dorsal view.

**Figures 94–98.**
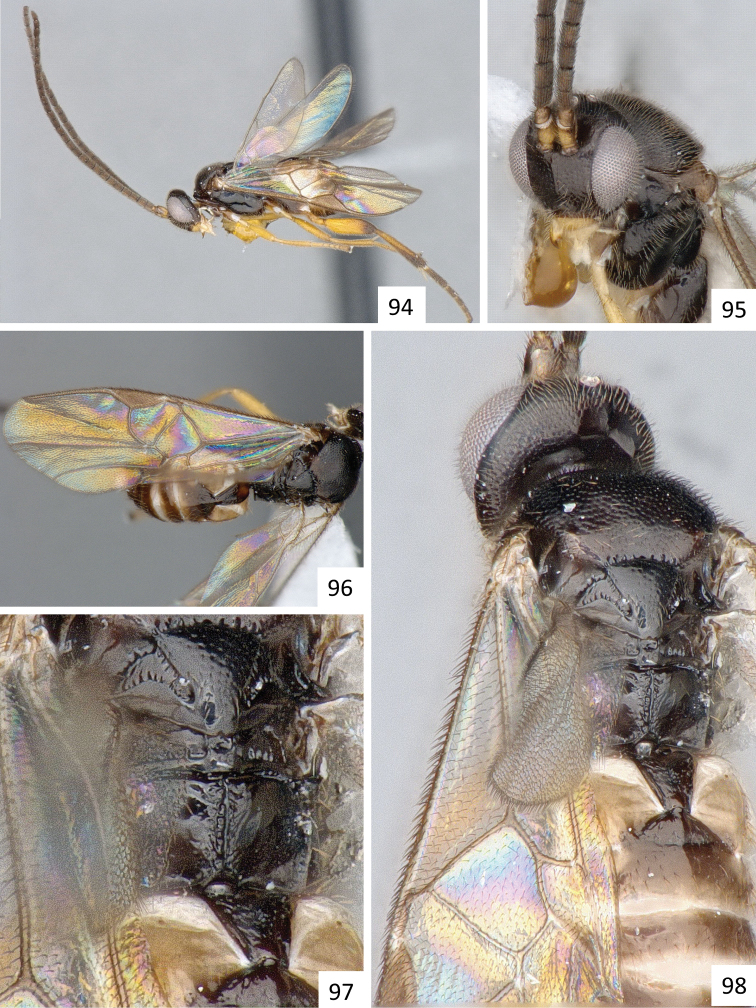
*Pseudapanteles mariobozai*. **94** Habitus, lateral view **95** Head, frontal view **96** Wings **97** Mesosoma and metasoma (partially), dorsal view **98** Habitus (partially), dorsal view.

**Figures 99–104.**
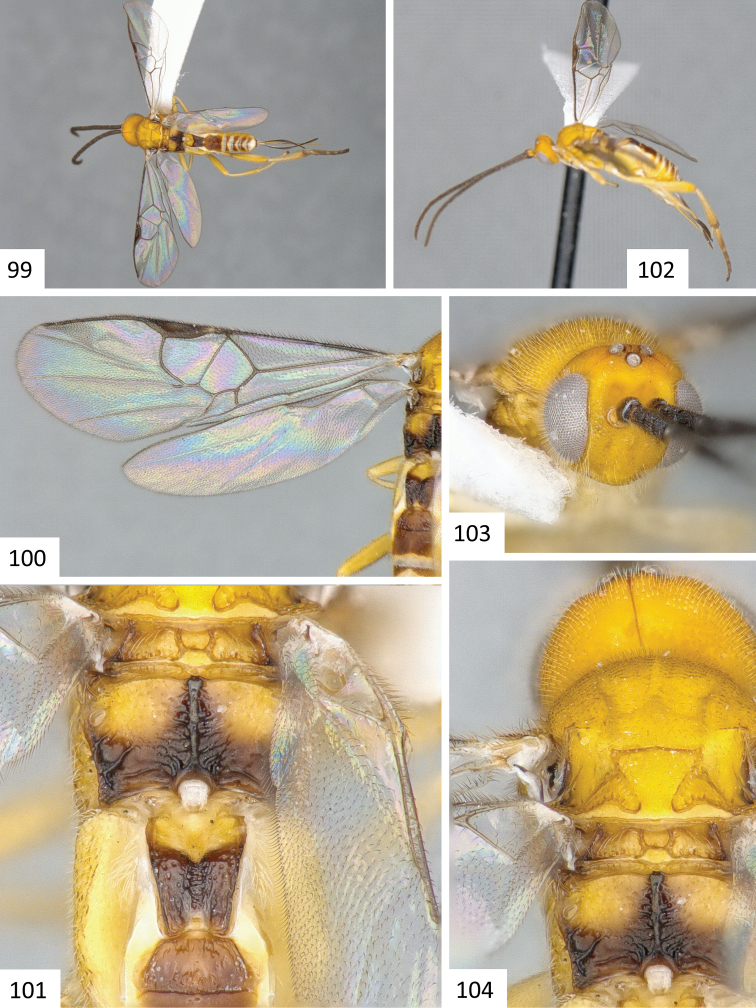
*Pseudapanteles mariocarvajali*. **99** Habitus, dorsal view **100** Wings **101** Propodeum, mediotergires 1–2, dorsal view **102** Habitus, lateral view **103** Head, frontal view **104** Head and mesosoma, dorsal view.

**Figures 105–109.**
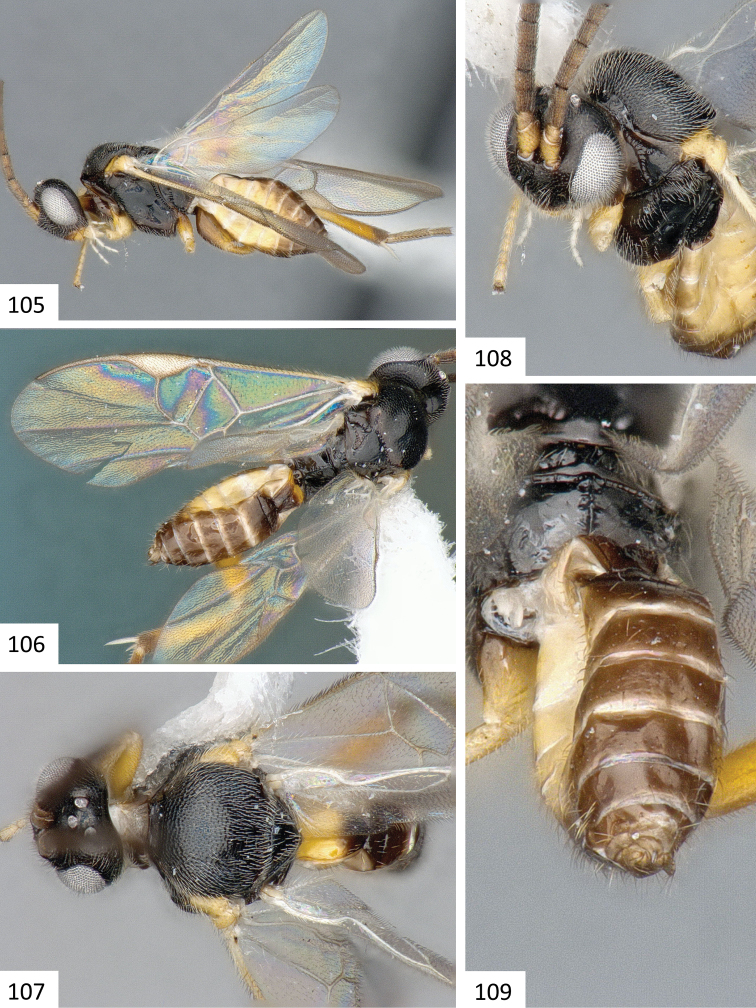
*Pseudapanteles maureenballesteroae*. **105** Habitus, lateral view **106** Wings **107** Habitus, dorsal view **108** Habitus, frontal-lateral view **109** Propodeum (partially) and metasoma, dorsal view.

**Figures 110–115.**
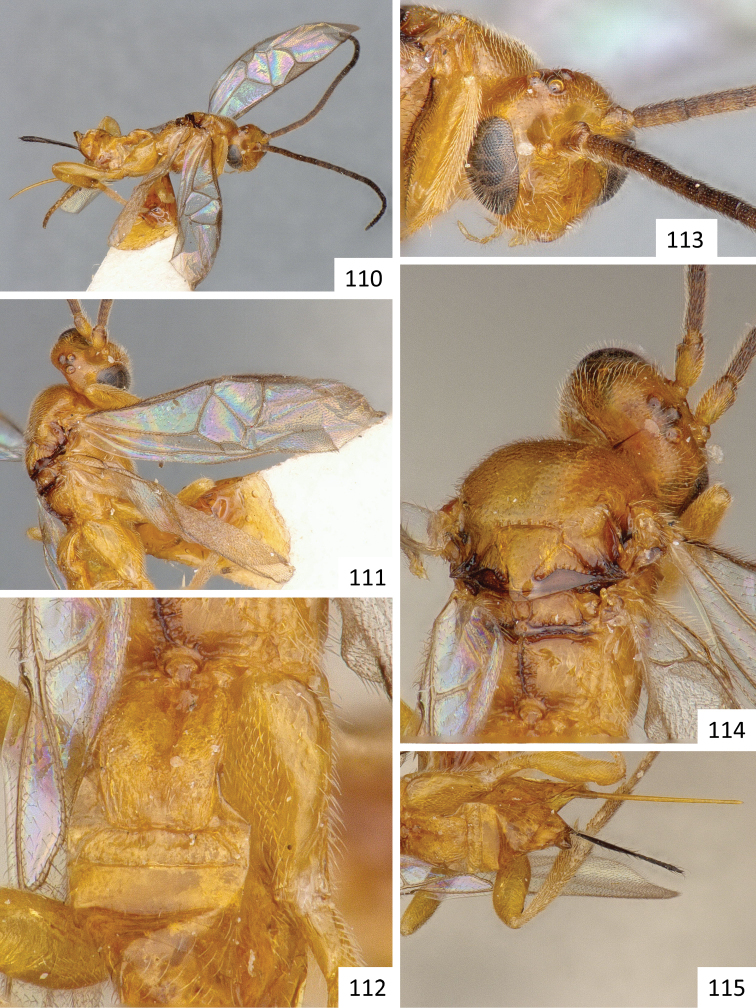
*Pseudapanteles moerens*. **110** Habitus, dorso-lateral view **111** Wings **112** Mediotergites 1–4, dorsal view **113** Head, frontal view **114** Head and mesosoma, dorsal view **115** Metasoma, dorso-lateral view.

**Figures 116–120.**
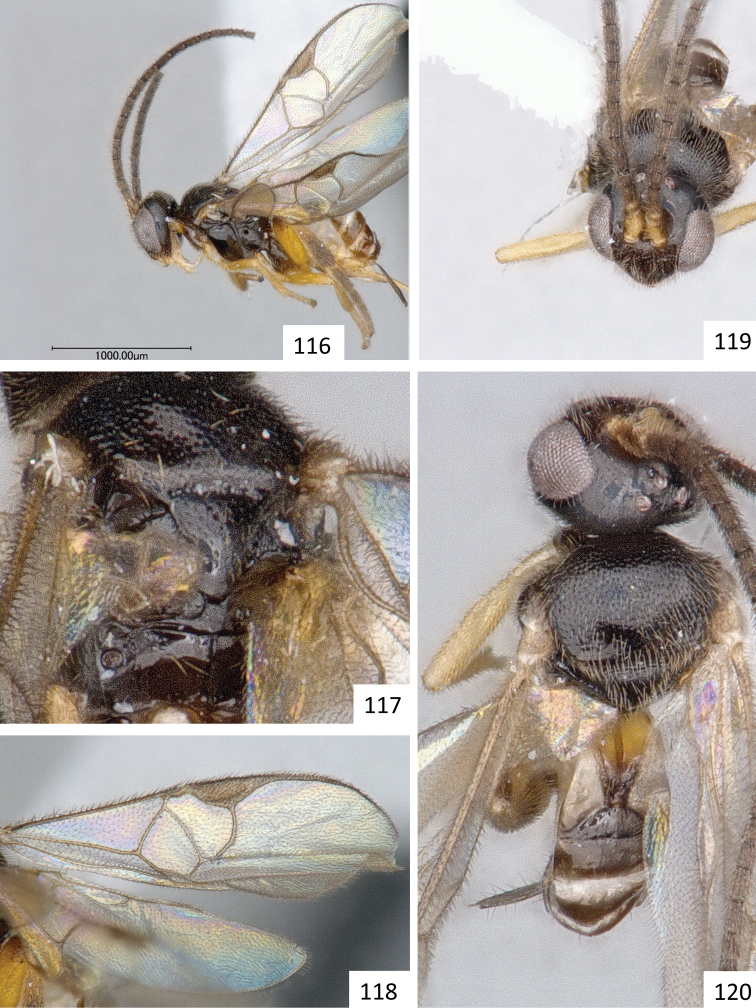
*Pseudapanteles munifigueresae*. **116** Habitus, lateral view **117** Mesosoma, dorsal view **118** Wings **119** Head and mesosoma (partially), frontal view **120** Habitus, dorsal view.

**Figures 121–125.**
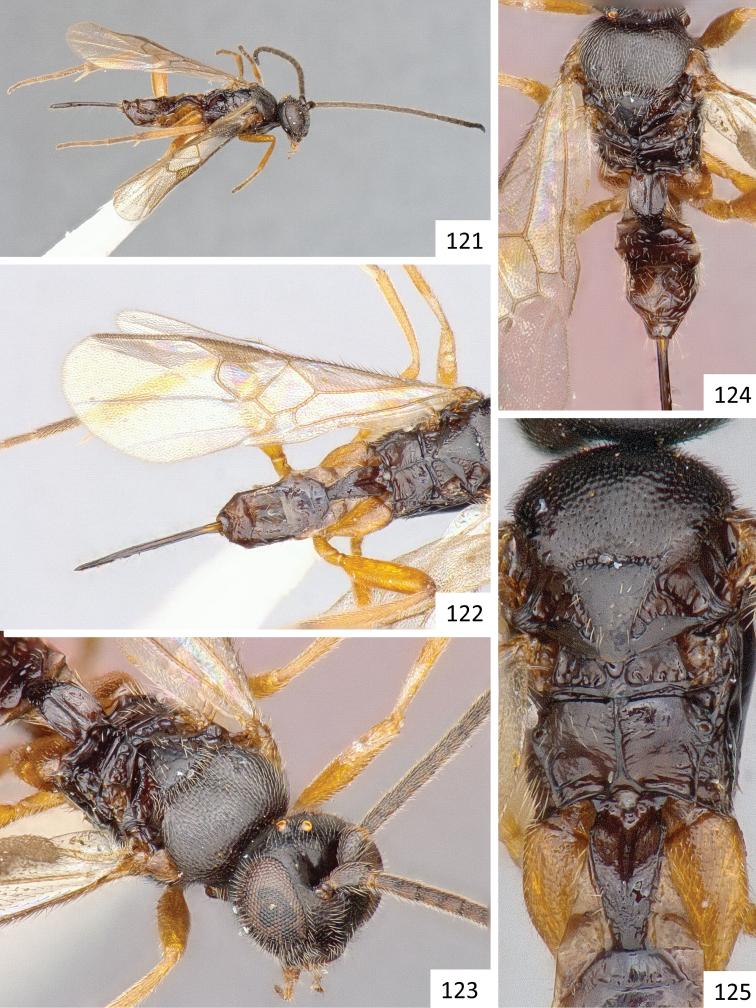
*Pseudapanteles nerion*. **121** Habitus, dorso-lateral view **122** Wings (partial habitus shown in dorsal view) **123** Head, mesosoma and metasoma (partially), frontal-lateral view **124** Mesosoma and metasoma, dorsal view **125** Details of mesosoma and mediotergites 1–3, dorsal view.

**Figures 126–130.**
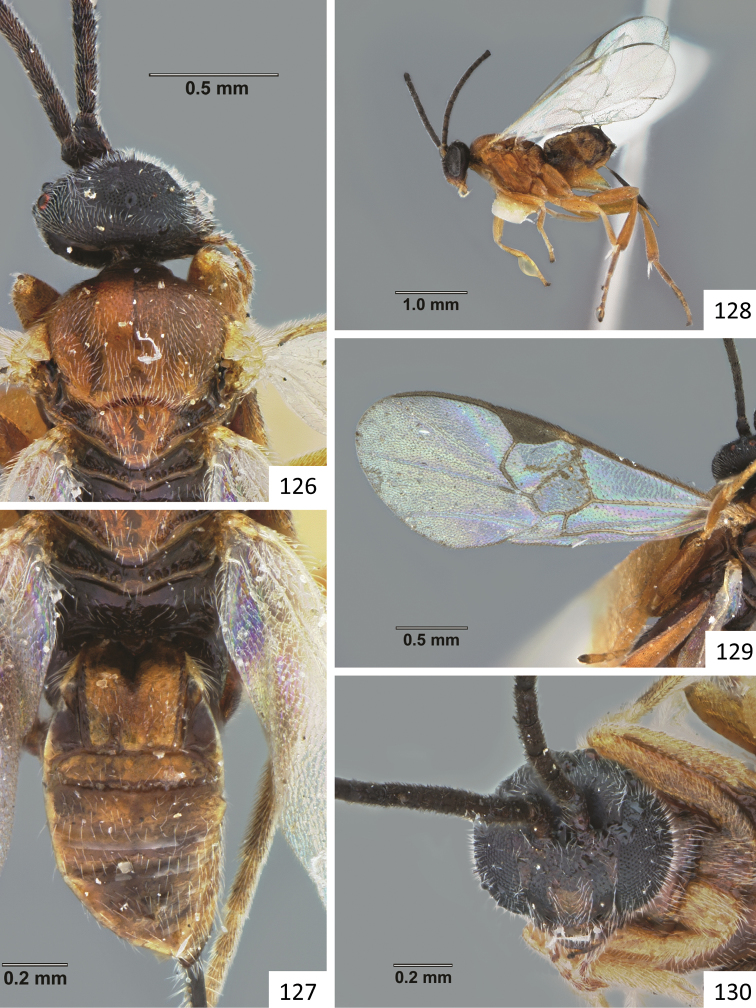
*Pseudapanteles nigrovariatus*. **126** Head and mesosoma (partially), dorsal view **127** Propodeum and metasoma, dorsal view **128** Habitus, dorsal view **129** Fore wing **130** Head, frontal view.

**Figures 131–135.**
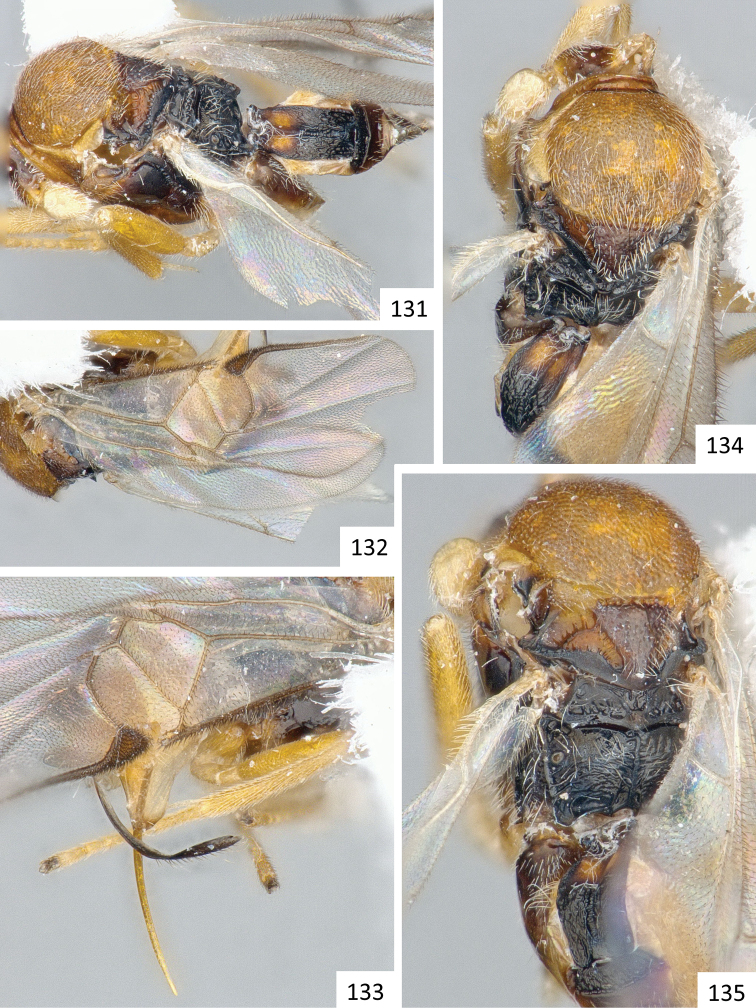
*Pseudapanteles oscarariasi*. **131** Habitus, dorso-lateral view **132** Wings **133** Hypopygium and ovipositor (partially), lateral view **134** Mesosoma, lateral view **135** Mesosoma and metasoma (partially), dorsal view.

**Figures 136–140.**
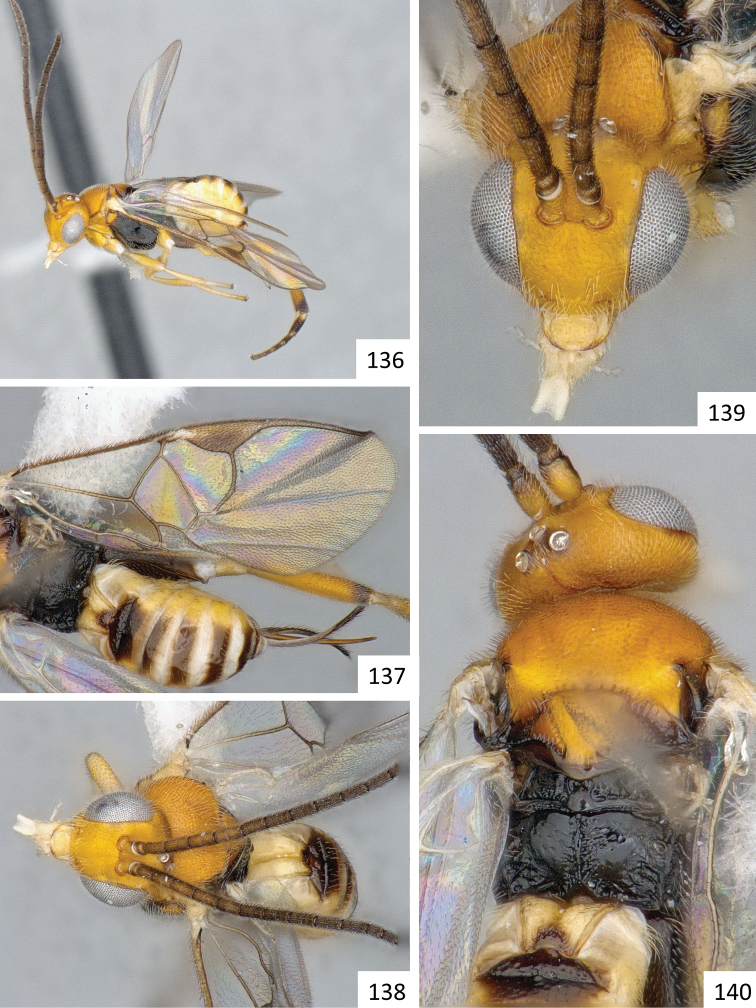
*Pseudapanteles ottonsolisi*. **136** Habitus, lateral view **137** Fore wing **138** Habitus, frontal-lateral view **139** Head, frontal view **140** Head, mesosoma and mediotergites 1–2, dorsal view.

**Figures 141–145.**
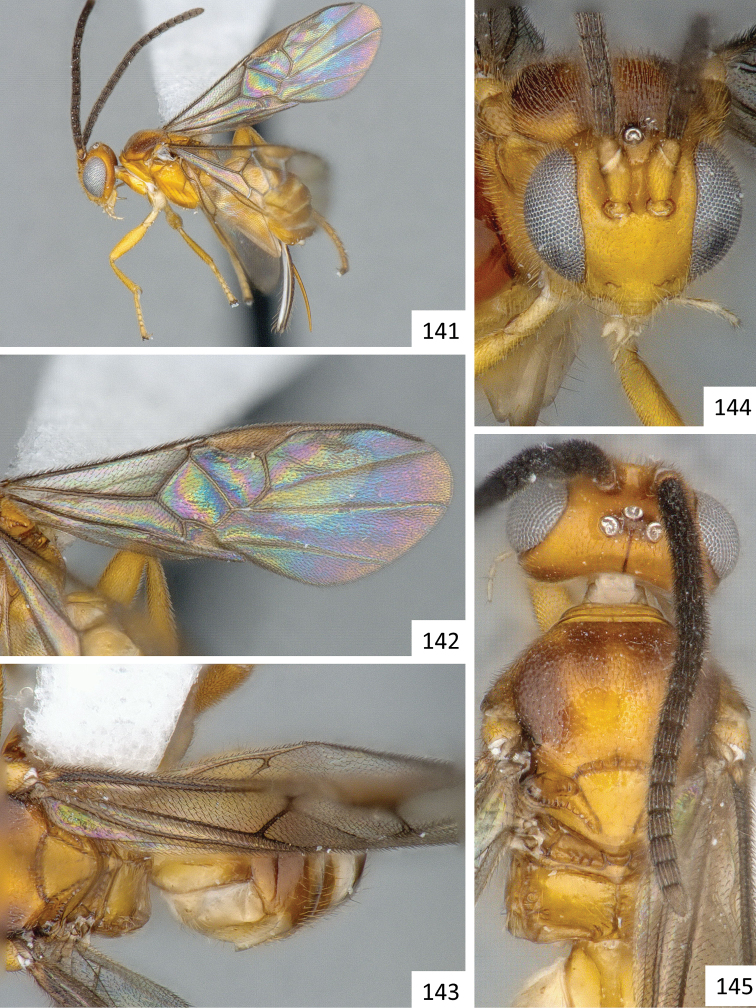
*Pseudapanteles pedroleoni*. **141** Habitus, lateral view **142** Fore wing **143** Mesosoma and metasoma (partially), dorsal view **144** head, frontal view **145** Head and mesosoma, dorsal view.

**Figures 146–150.**
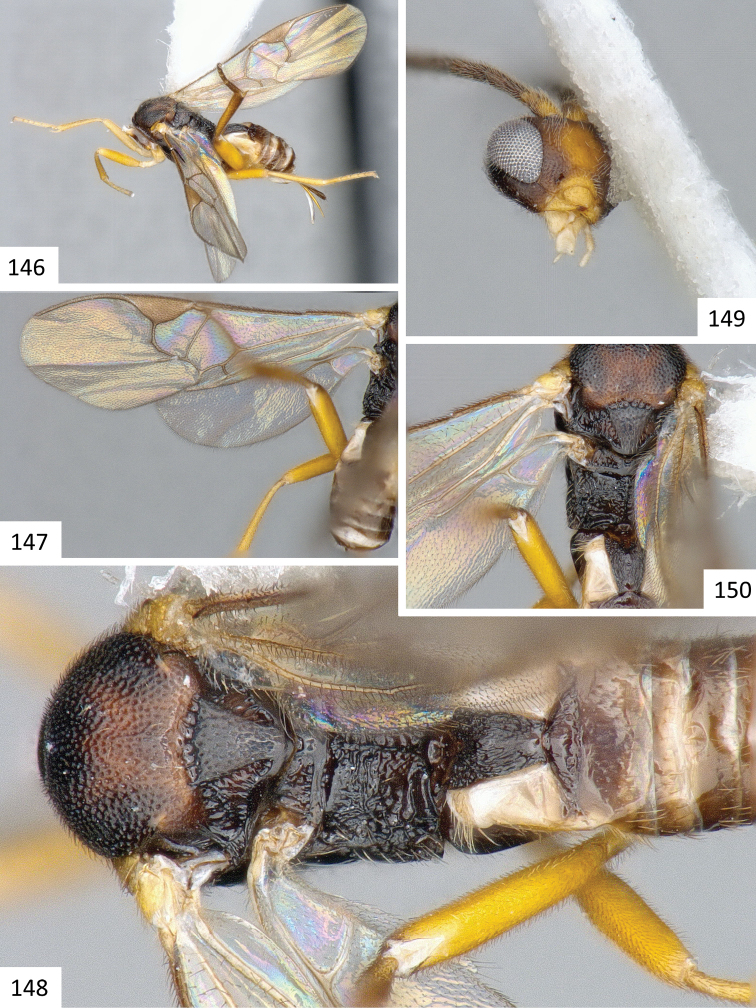
*Pseudapanteles raulsolorzanoi*. **146** Habitus, lateral view **147** Wings **148** Mesosoma and metasoma (partially), dorsal view **149** Head (partially), dorsal view **150** Mesosoma and mediotergites 1–2, dorsal view.

**Figures 151–155.**
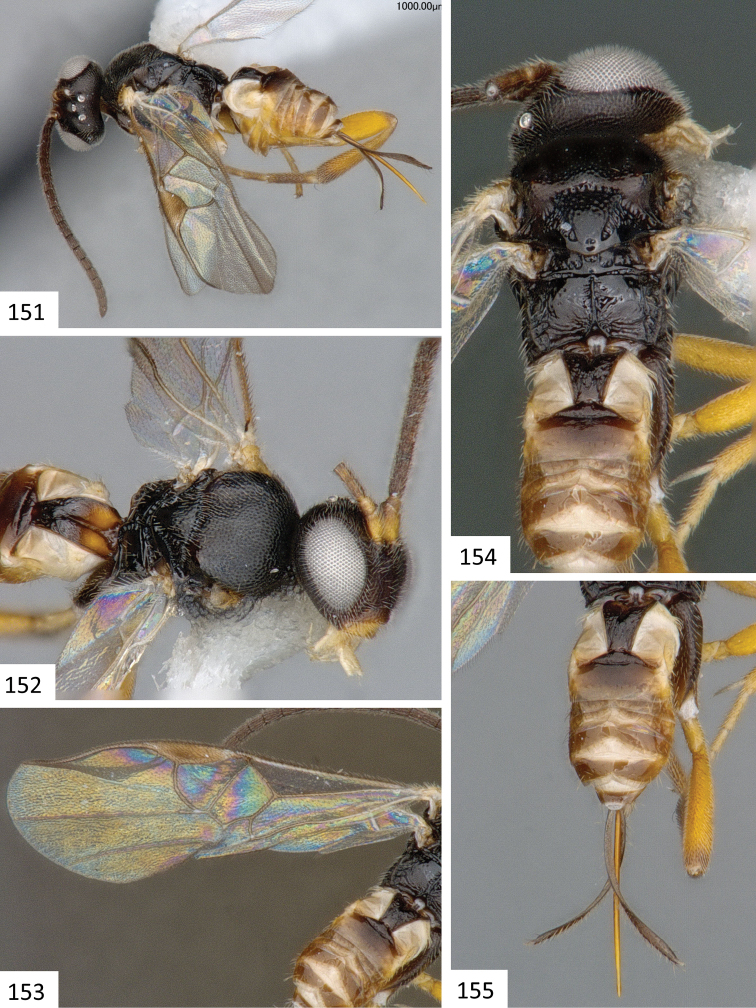
*Pseudapanteles renecastroi*. **151** Habitus, dorso-lateral view **152** Head, lateral; mesosoma and mediotergites 1–4, dorso-lateral view **153** Wings **154** Habitus, dorsal view **155** Metasoma, dorsal view.

**Figures 156–161.**
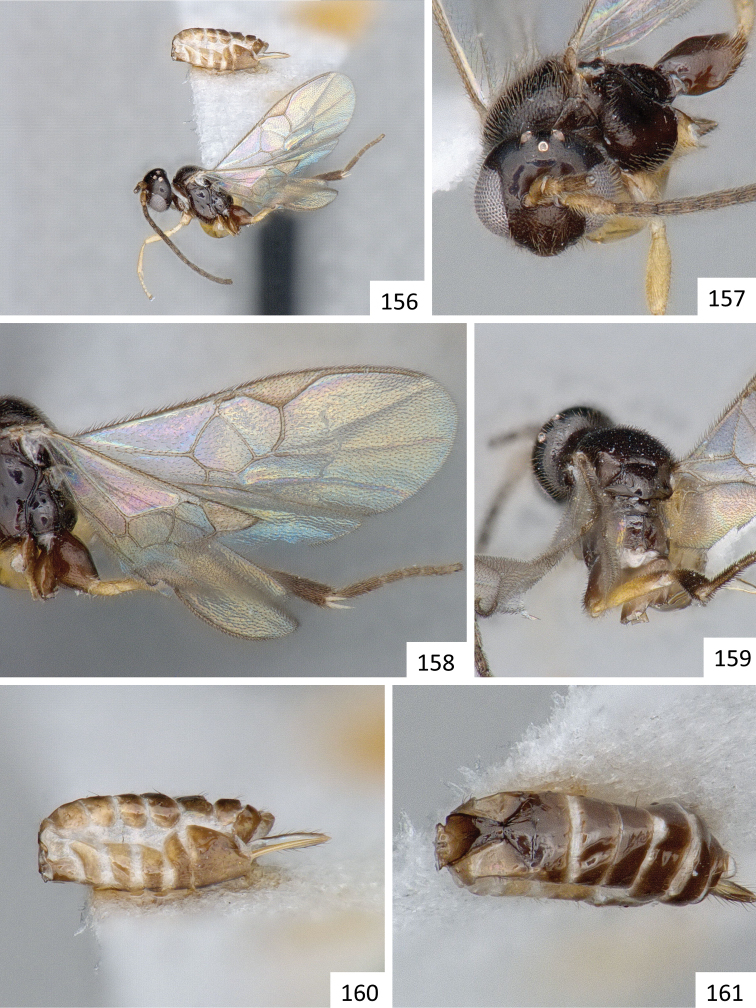
*Pseudapanteles rodrigogamezi*. **156** Habitus, lateral view **157** Head, frontal view **158** Wings **159** Mesosoma, dorsal view **160** Metasoma, lateral view **161** Metasoma, dorsal view.

**Figures 162–165.**
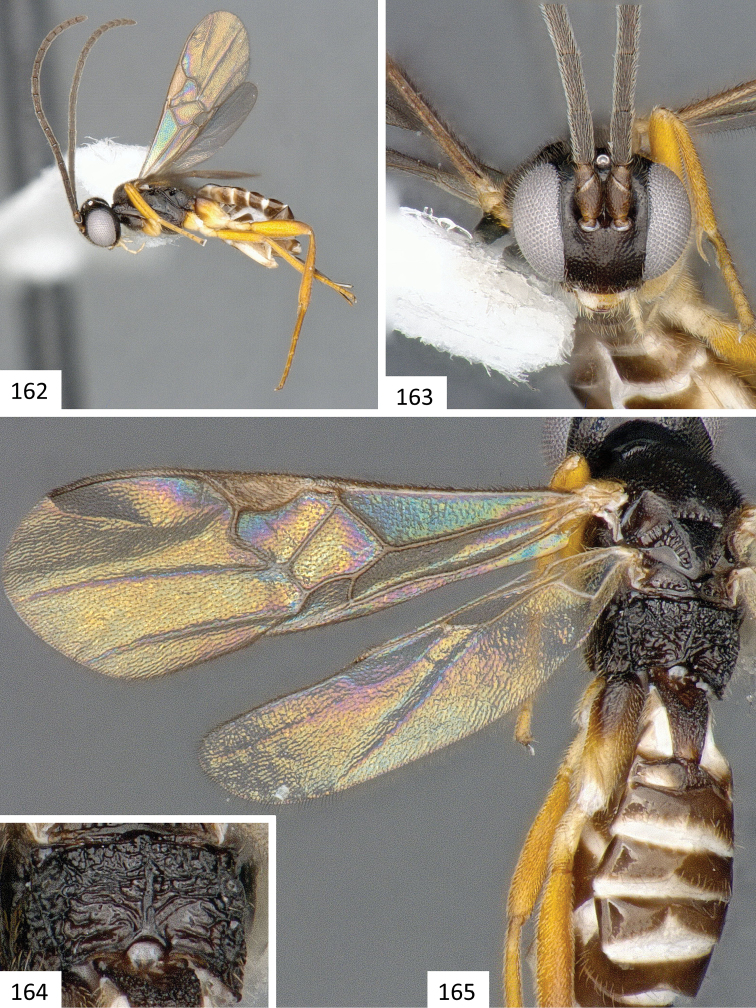
*Pseudapanteles rosemarykarpinskiae*. **162** Habitus, lateral view **163** Head, frontal view **164** Propodeum **165** Wings and most of body, dorsal view.

**Figures 169–173.**
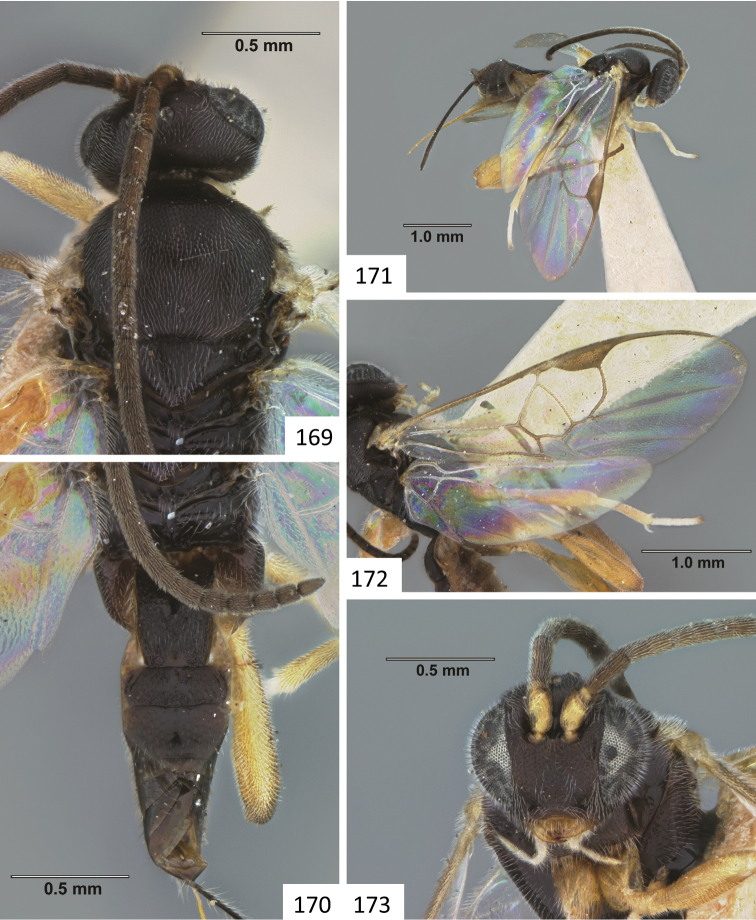
*Pseudapanteles sesiae*. **169** Head and mesosoma (partially), dorsal view **170** Propodeum and metasoma, dorsal view **171** Habitus, lateral view **172** Wings **173** Head, frontal view.

**Figures 174–179.**
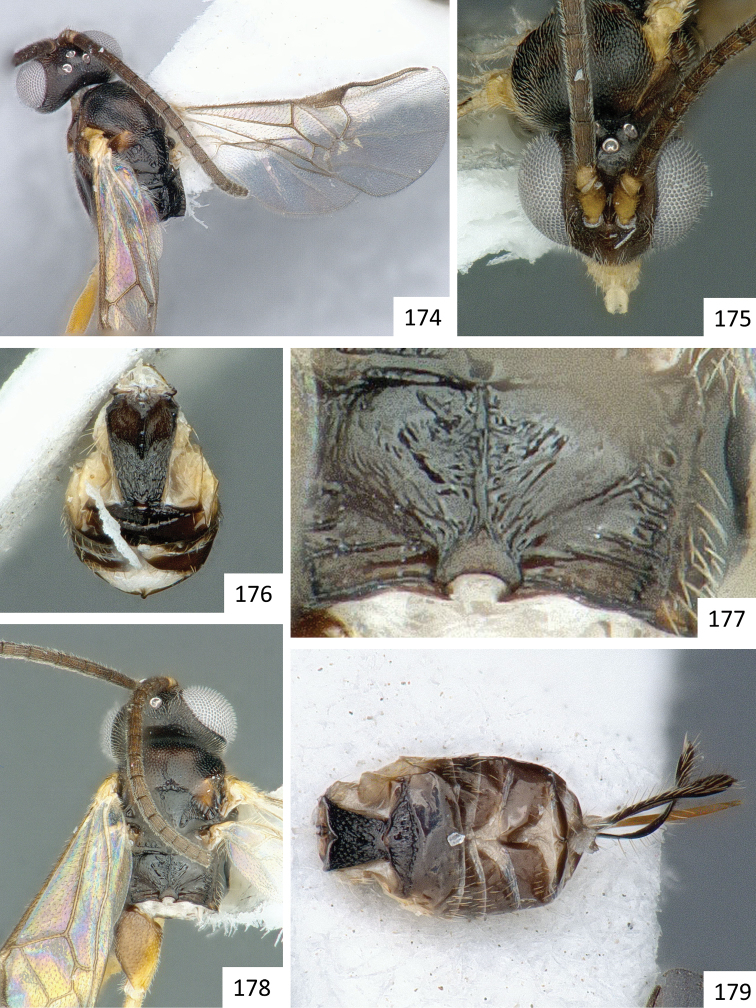
*Pseudapanteles soniapicadoae*. **174** Head and mesosoma, dorso-lateral view **175** Head, frontal view **176** Metasoma (partially), dorsal view **177** Propodeum **178** Head and mesosoma, dorsal view **179** Metasoma, dorsal view.

**Figures 180–185.**
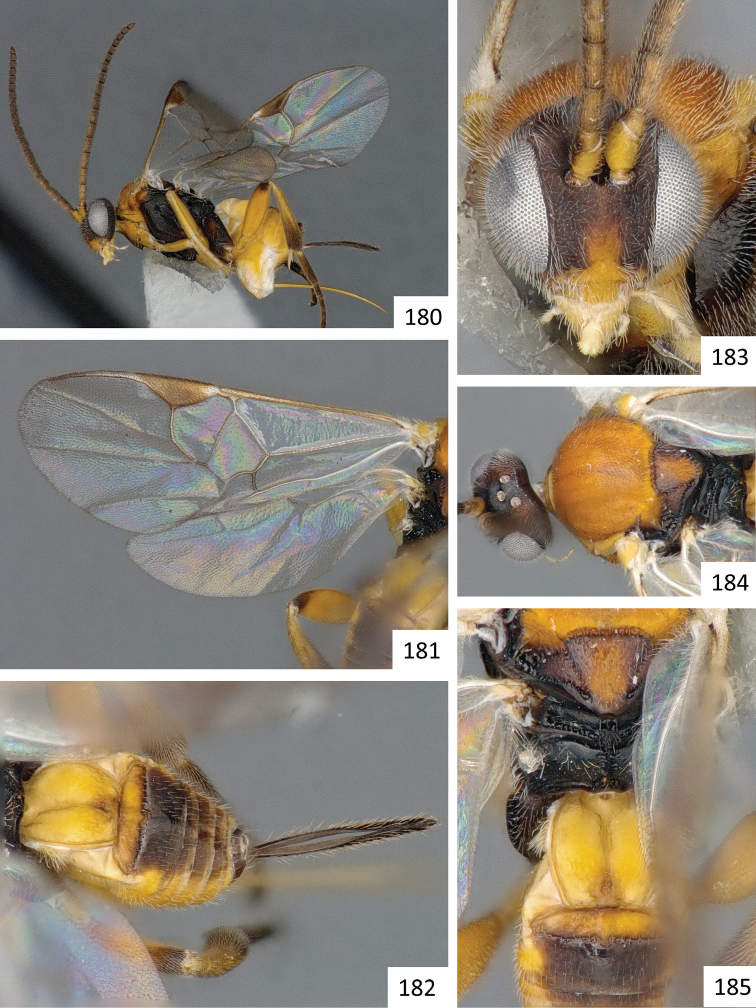
*Pseudapanteles teofilodelatorrei*. **180** Habitus, lateral view **181** Wings **182** Metasoma, dorso-lateral view **183** Head, frontal view **184** Head and mesosoma, dorsal view **185** Mesosoma and metasoma (partially), dorsal view.

## Supplementary materials

Supplementary material 1 BOLD TaxonID Tree Data type: phylogenetic data Explanation note: K2P of Pseudapanteles sequences. This dataset is made available under the Open Database License (http://opendatacommons.org/licenses/odbl/1.0/). The Open Database License (ODbL) is a license agreement intended to allow users to freely share, modify, and use this Dataset while maintaining this same freedom for others, provided that the original source and author(s) are credited.Jose L. Fernández-Triana, Daniel H. Janzen, Winnie Hallwachs, James B. Whitfield, M. Alex Smith, Robert Kula

Supplementary material 2 All sequence and specimen information Data type: specimens data Explanation note: Excel file with complete information of the specimens with DNA barcodes. This dataset is made available under the Open Database License (http://opendatacommons.org/licenses/odbl/1.0/). The Open Database License (ODbL) is a license agreement intended to allow users to freely share, modify, and use this Dataset while maintaining this same freedom for others, provided that the original source and author(s) are credited.Jose L. Fernández-Triana, Daniel H. Janzen, Winnie Hallwachs, James B. Whitfield, M. Alex Smith, Robert Kula
