# Supplementary material for: Revision of the genus Pseudapanteles (Hymenoptera, Braconidae, Microgastrinae), with emphasis on the species in Area de Conservación Guanacaste, northwestern Costa Rica
Source: Zookeys. 2014 Oct 14;(446):1–82. doi: 10.3897/zookeys.446.8195 (PMC4205727; doi:10.3897/zookeys.446.8195)
Supplement: Supplementary material 1 — BOLD TaxonID Tree [file zookeys-446-001-s001.pdf]

BOLD TaxonID Tree

```
Title            : SEARCH: Process ids(378 ids) [SEARCH1]
Date             : 24-June-2014
Data Type        : Nucleotide
Distance Model   : Kimura 2 Parameter
Marker           : COI-5P
Codon Positions  : 1st, 2nd, 3rd
Labels           : Extra Info, SampleID, Sequence Length
Filters          : Length > 200
Colorization     : [blue]=Stop Codons [red]=Contamination or misidentification

Sequence Count   : 378
Species count    : 33
Genus count      : 1
Family count     : 1
Unidentified     : 0
```

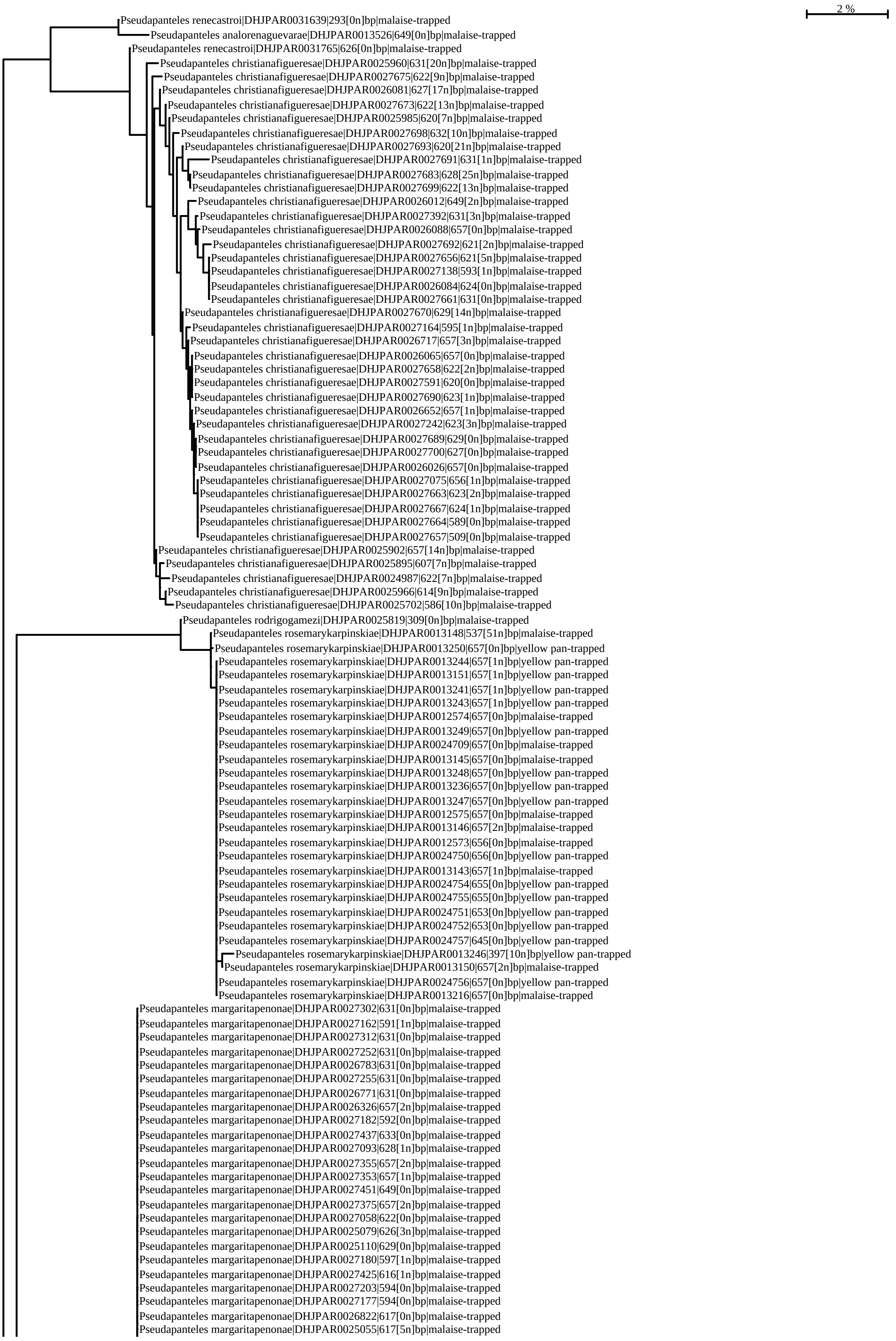
2 %
Pseudapanteles renecastroi|DHJPAR0031639|293[0n]bp|malaise-trapped
Pseudapanteles analorenaguevarae|DHJPAR0013526|649[0n]bp|malaise-trapped
Pseudapanteles renecastroi|DHJPAR0031765|626[0n]bp|malaise-trapped
Pseudapanteles christianafigueresae|DHJPAR0025960|631[20n]bp|malaise-trapped
Pseudapanteles christianafigueresae|DHJPAR0027675|622[9n]bp|malaise-trapped
Pseudapanteles christianafigueresae|DHJPAR0026081|627[17n]bp|malaise-trapped
Pseudapanteles christianafigueresae|DHJPAR0027673|622[13n]bp|malaise-trapped
Pseudapanteles christianafigueresae|DHJPAR0025985|620[7n]bp|malaise-trapped
Pseudapanteles christianafigueresae|DHJPAR0027698|632[10n]bp|malaise-trapped
Pseudapanteles christianafigueresae|DHJPAR0027693|620[21n]bp|malaise-trapped
Pseudapanteles christianafigueresae|DHJPAR0027691|631[1n]bp|malaise-trapped
Pseudapanteles christianafigueresae|DHJPAR0027683|628[25n]bp|malaise-trapped
Pseudapanteles christianafigueresae|DHJPAR0027699|622[13n]bp|malaise-trapped
Pseudapanteles christianafigueresae|DHJPAR0026012|649[2n]bp|malaise-trapped
Pseudapanteles christianafigueresae|DHJPAR0027392|631[3n]bp|malaise-trapped
Pseudapanteles christianafigueresae|DHJPAR0026088|657[0n]bp|malaise-trapped
Pseudapanteles christianafigueresae|DHJPAR0027692|621[2n]bp|malaise-trapped
Pseudapanteles christianafigueresae|DHJPAR0027656|621[5n]bp|malaise-trapped
Pseudapanteles christianafigueresae|DHJPAR0027138|593[1n]bp|malaise-trapped
Pseudapanteles christianafigueresae|DHJPAR0026084|624[0n]bp|malaise-trapped
Pseudapanteles christianafigueresae|DHJPAR0027661|631[0n]bp|malaise-trapped
Pseudapanteles christianafigueresae|DHJPAR0027670|629[14n]bp|malaise-trapped
Pseudapanteles christianafigueresae|DHJPAR0027164|595[1n]bp|malaise-trapped
Pseudapanteles christianafigueresae|DHJPAR0026717|657[3n]bp|malaise-trapped
Pseudapanteles christianafigueresae|DHJPAR0026065|657[0n]bp|malaise-trapped
Pseudapanteles christianafigueresae|DHJPAR0027658|622[2n]bp|malaise-trapped
Pseudapanteles christianafigueresae|DHJPAR0027591|620[0n]bp|malaise-trapped
Pseudapanteles christianafigueresae|DHJPAR0027690|623[1n]bp|malaise-trapped
Pseudapanteles christianafigueresae|DHJPAR0026652|657[1n]bp|malaise-trapped
Pseudapanteles christianafigueresae|DHJPAR0027242|623[3n]bp|malaise-trapped
Pseudapanteles christianafigueresae|DHJPAR0027689|629[0n]bp|malaise-trapped
Pseudapanteles christianafigueresae|DHJPAR0027700|627[0n]bp|malaise-trapped
Pseudapanteles christianafigueresae|DHJPAR0026026|657[0n]bp|malaise-trapped
Pseudapanteles christianafigueresae|DHJPAR0027075|656[1n]bp|malaise-trapped
Pseudapanteles christianafigueresae|DHJPAR0027663|623[2n]bp|malaise-trapped
Pseudapanteles christianafigueresae|DHJPAR0027667|624[1n]bp|malaise-trapped
Pseudapanteles christianafigueresae|DHJPAR0027664|589[0n]bp|malaise-trapped
Pseudapanteles christianafigueresae|DHJPAR0027657|509[0n]bp|malaise-trapped
Pseudapanteles christianafigueresae|DHJPAR0025902|657[14n]bp|malaise-trapped
Pseudapanteles christianafigueresae|DHJPAR0025895|607[7n]bp|malaise-trapped
Pseudapanteles christianafigueresae|DHJPAR0024987|622[7n]bp|malaise-trapped
Pseudapanteles christianafigueresae|DHJPAR0025966|614[9n]bp|malaise-trapped
Pseudapanteles christianafigueresae|DHJPAR0025702|586[10n]bp|malaise-trapped
Pseudapanteles rodrigogamezi|DHJPAR0025819|309[0n]bp|malaise-trapped
Pseudapanteles rosemarykarpinskiae|DHJPAR0013148|537[51n]bp|malaise-trapped
Pseudapanteles rosemarykarpinskiae|DHJPAR0013250|657[0n]bp|yellow pan-trapped
Pseudapanteles rosemarykarpinskiae|DHJPAR0013244|657[1n]bp|yellow pan-trapped
Pseudapanteles rosemarykarpinskiae|DHJPAR0013151|657[1n]bp|yellow pan-trapped
Pseudapanteles rosemarykarpinskiae|DHJPAR0013241|657[1n]bp|yellow pan-trapped
Pseudapanteles rosemarykarpinskiae|DHJPAR0013243|657[1n]bp|yellow pan-trapped
Pseudapanteles rosemarykarpinskiae|DHJPAR0012574|657[0n]bp|malaise-trapped
Pseudapanteles rosemarykarpinskiae|DHJPAR0013249|657[0n]bp|yellow pan-trapped
Pseudapanteles rosemarykarpinskiae|DHJPAR0024709|657[0n]bp|malaise-trapped
Pseudapanteles rosemarykarpinskiae|DHJPAR0013145|657[0n]bp|malaise-trapped
Pseudapanteles rosemarykarpinskiae|DHJPAR0013248|657[0n]bp|yellow pan-trapped
Pseudapanteles rosemarykarpinskiae|DHJPAR0013236|657[0n]bp|yellow pan-trapped
Pseudapanteles rosemarykarpinskiae|DHJPAR0013247|657[0n]bp|yellow pan-trapped
Pseudapanteles rosemarykarpinskiae|DHJPAR0012575|657[0n]bp|malaise-trapped
Pseudapanteles rosemarykarpinskiae|DHJPAR0013146|657[2n]bp|malaise-trapped
Pseudapanteles rosemarykarpinskiae|DHJPAR0012573|656[0n]bp|malaise-trapped
Pseudapanteles rosemarykarpinskiae|DHJPAR0024750|656[0n]bp|yellow pan-trapped
Pseudapanteles rosemarykarpinskiae|DHJPAR0013143|657[1n]bp|malaise-trapped
Pseudapanteles rosemarykarpinskiae|DHJPAR0024754|655[0n]bp|yellow pan-trapped
Pseudapanteles rosemarykarpinskiae|DHJPAR0024755|655[0n]bp|yellow pan-trapped
Pseudapanteles rosemarykarpinskiae|DHJPAR0024751|653[0n]bp|yellow pan-trapped
Pseudapanteles rosemarykarpinskiae|DHJPAR0024752|653[0n]bp|yellow pan-trapped
Pseudapanteles rosemarykarpinskiae|DHJPAR0024757|645[0n]bp|yellow pan-trapped
Pseudapanteles rosemarykarpinskiae|DHJPAR0013246|397[10n]bp|yellow pan-trapped
Pseudapanteles rosemarykarpinskiae|DHJPAR0013150|657[2n]bp|malaise-trapped
Pseudapanteles rosemarykarpinskiae|DHJPAR0024756|657[0n]bp|yellow pan-trapped
Pseudapanteles rosemarykarpinskiae|DHJPAR0013216|657[0n]bp|malaise-trapped
Pseudapanteles margaritapenonae|DHJPAR0027302|631[0n]bp|malaise-trapped
Pseudapanteles margaritapenonae|DHJPAR0027162|591[1n]bp|malaise-trapped
Pseudapanteles margaritapenonae|DHJPAR0027312|631[0n]bp|malaise-trapped
Pseudapanteles margaritapenonae|DHJPAR0027252|631[0n]bp|malaise-trapped
Pseudapanteles margaritapenonae|DHJPAR0026783|631[0n]bp|malaise-trapped
Pseudapanteles margaritapenonae|DHJPAR0027255|631[0n]bp|malaise-trapped
Pseudapanteles margaritapenonae|DHJPAR0026771|631[0n]bp|malaise-trapped
Pseudapanteles margaritapenonae|DHJPAR0026326|657[2n]bp|malaise-trapped
Pseudapanteles margaritapenonae|DHJPAR0027182|592[0n]bp|malaise-trapped
Pseudapanteles margaritapenonae|DHJPAR0027437|633[0n]bp|malaise-trapped
Pseudapanteles margaritapenonae|DHJPAR0027093|628[1n]bp|malaise-trapped
Pseudapanteles margaritapenonae|DHJPAR0027355|657[2n]bp|malaise-trapped
Pseudapanteles margaritapenonae|DHJPAR0027353|657[1n]bp|malaise-trapped
Pseudapanteles margaritapenonae|DHJPAR0027451|649[0n]bp|malaise-trapped
Pseudapanteles margaritapenonae|DHJPAR0027375|657[2n]bp|malaise-trapped
Pseudapanteles margaritapenonae|DHJPAR0027058|622[0n]bp|malaise-trapped
Pseudapanteles margaritapenonae|DHJPAR0025079|626[3n]bp|malaise-trapped
Pseudapanteles margaritapenonae|DHJPAR0025110|629[0n]bp|malaise-trapped
Pseudapanteles margaritapenonae|DHJPAR0027180|597[1n]bp|malaise-trapped
Pseudapanteles margaritapenonae|DHJPAR0027425|616[1n]bp|malaise-trapped
Pseudapanteles margaritapenonae|DHJPAR0027203|594[0n]bp|malaise-trapped
Pseudapanteles margaritapenonae|DHJPAR0027177|594[0n]bp|malaise-trapped
Pseudapanteles margaritapenonae|DHJPAR0026822|617[0n]bp|malaise-trapped
Pseudapanteles margaritapenonae|DHJPAR0025055|617[5n]bp|malaise-trapped

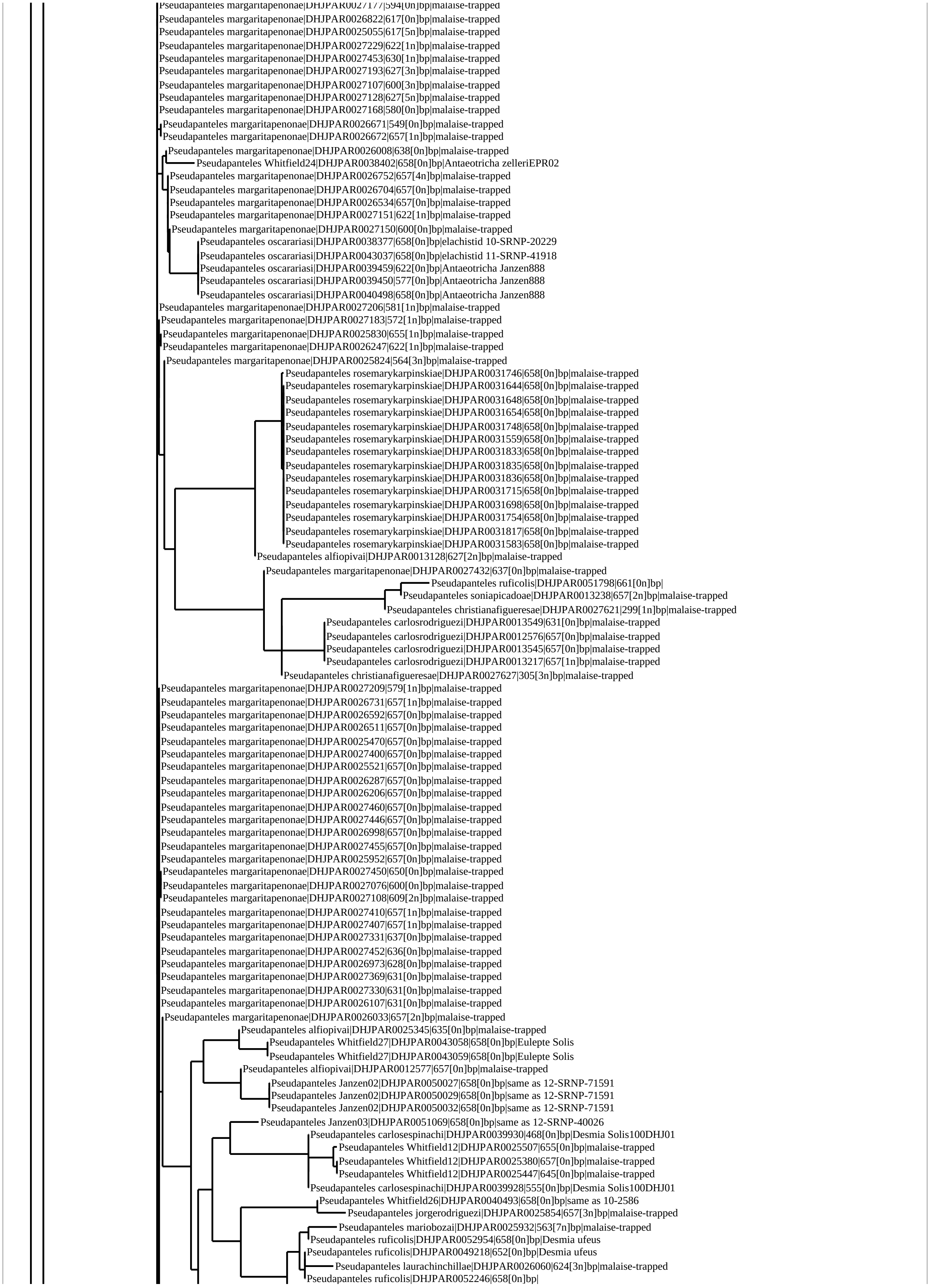
Pseudapanteles margaritapenonae|DHJPAR0027177|594[0n]bp|malaise-trapped
Pseudapanteles margaritapenonae|DHJPAR0026822|617[0n]bp|malaise-trapped
Pseudapanteles margaritapenonae|DHJPAR0025055|617[5n]bp|malaise-trapped
Pseudapanteles margaritapenonae|DHJPAR0027229|622[1n]bp|malaise-trapped
Pseudapanteles margaritapenonae|DHJPAR0027453|630[1n]bp|malaise-trapped
Pseudapanteles margaritapenonae|DHJPAR0027193|627[3n]bp|malaise-trapped
Pseudapanteles margaritapenonae|DHJPAR0027107|600[3n]bp|malaise-trapped
Pseudapanteles margaritapenonae|DHJPAR0027128|627[5n]bp|malaise-trapped
Pseudapanteles margaritapenonae|DHJPAR0027168|580[0n]bp|malaise-trapped
Pseudapanteles margaritapenonae|DHJPAR0026671|549[0n]bp|malaise-trapped
Pseudapanteles margaritapenonae|DHJPAR0026672|657[1n]bp|malaise-trapped
Pseudapanteles margaritapenonae|DHJPAR0026008|638[0n]bp|malaise-trapped
Pseudapanteles Whitfield24|DHJPAR0038402|658[0n]bp|Antaeotricha zelleriEPR02
Pseudapanteles margaritapenonae|DHJPAR0026752|657[4n]bp|malaise-trapped
Pseudapanteles margaritapenonae|DHJPAR0026704|657[0n]bp|malaise-trapped
Pseudapanteles margaritapenonae|DHJPAR0026534|657[0n]bp|malaise-trapped
Pseudapanteles margaritapenonae|DHJPAR0027151|622[1n]bp|malaise-trapped
Pseudapanteles margaritapenonae|DHJPAR0027150|600[0n]bp|malaise-trapped
Pseudapanteles oscarariasi|DHJPAR0038377|658[0n]bp|elachistid 10-SRNP-20229
Pseudapanteles oscarariasi|DHJPAR0043037|658[0n]bp|elachistid 11-SRNP-41918
Pseudapanteles oscarariasi|DHJPAR0039459|622[0n]bp|Antaeotricha Janzen888
Pseudapanteles oscarariasi|DHJPAR0039450|577[0n]bp|Antaeotricha Janzen888
Pseudapanteles oscarariasi|DHJPAR0040498|658[0n]bp|Antaeotricha Janzen888
Pseudapanteles margaritapenonae|DHJPAR0027206|581[1n]bp|malaise-trapped
Pseudapanteles margaritapenonae|DHJPAR0027183|572[1n]bp|malaise-trapped
Pseudapanteles margaritapenonae|DHJPAR0025830|655[1n]bp|malaise-trapped
Pseudapanteles margaritapenonae|DHJPAR0026247|622[1n]bp|malaise-trapped
Pseudapanteles margaritapenonae|DHJPAR0025824|564[3n]bp|malaise-trapped
Pseudapanteles rosemarykarpinskiae|DHJPAR0031746|658[0n]bp|malaise-trapped
Pseudapanteles rosemarykarpinskiae|DHJPAR0031644|658[0n]bp|malaise-trapped
Pseudapanteles rosemarykarpinskiae|DHJPAR0031648|658[0n]bp|malaise-trapped
Pseudapanteles rosemarykarpinskiae|DHJPAR0031654|658[0n]bp|malaise-trapped
Pseudapanteles rosemarykarpinskiae|DHJPAR0031748|658[0n]bp|malaise-trapped
Pseudapanteles rosemarykarpinskiae|DHJPAR0031559|658[0n]bp|malaise-trapped
Pseudapanteles rosemarykarpinskiae|DHJPAR0031833|658[0n]bp|malaise-trapped
Pseudapanteles rosemarykarpinskiae|DHJPAR0031835|658[0n]bp|malaise-trapped
Pseudapanteles rosemarykarpinskiae|DHJPAR0031836|658[0n]bp|malaise-trapped
Pseudapanteles rosemarykarpinskiae|DHJPAR0031715|658[0n]bp|malaise-trapped
Pseudapanteles rosemarykarpinskiae|DHJPAR0031698|658[0n]bp|malaise-trapped
Pseudapanteles rosemarykarpinskiae|DHJPAR0031754|658[0n]bp|malaise-trapped
Pseudapanteles rosemarykarpinskiae|DHJPAR0031817|658[0n]bp|malaise-trapped
Pseudapanteles rosemarykarpinskiae|DHJPAR0031583|658[0n]bp|malaise-trapped
Pseudapanteles alfiopivai|DHJPAR0013128|627[2n]bp|malaise-trapped
Pseudapanteles margaritapenonae|DHJPAR0027432|637[0n]bp|malaise-trapped
Pseudapanteles ruficolis|DHJPAR0051798|661[0n]bp|
Pseudapanteles soniapicadoae|DHJPAR0013238|657[2n]bp|malaise-trapped
Pseudapanteles christianafigueresae|DHJPAR0027621|299[1n]bp|malaise-trapped
Pseudapanteles carlosrodriguezi|DHJPAR0013549|631[0n]bp|malaise-trapped
Pseudapanteles carlosrodriguezi|DHJPAR0012576|657[0n]bp|malaise-trapped
Pseudapanteles carlosrodriguezi|DHJPAR0013545|657[0n]bp|malaise-trapped
Pseudapanteles carlosrodriguezi|DHJPAR0013217|657[1n]bp|malaise-trapped
Pseudapanteles christianafigueresae|DHJPAR0027627|305[3n]bp|malaise-trapped
Pseudapanteles margaritapenonae|DHJPAR0027209|579[1n]bp|malaise-trapped
Pseudapanteles margaritapenonae|DHJPAR0026731|657[1n]bp|malaise-trapped
Pseudapanteles margaritapenonae|DHJPAR0026592|657[0n]bp|malaise-trapped
Pseudapanteles margaritapenonae|DHJPAR0026511|657[0n]bp|malaise-trapped
Pseudapanteles margaritapenonae|DHJPAR0025470|657[0n]bp|malaise-trapped
Pseudapanteles margaritapenonae|DHJPAR0027400|657[0n]bp|malaise-trapped
Pseudapanteles margaritapenonae|DHJPAR0025521|657[0n]bp|malaise-trapped
Pseudapanteles margaritapenonae|DHJPAR0026287|657[0n]bp|malaise-trapped
Pseudapanteles margaritapenonae|DHJPAR0026206|657[0n]bp|malaise-trapped
Pseudapanteles margaritapenonae|DHJPAR0027460|657[0n]bp|malaise-trapped
Pseudapanteles margaritapenonae|DHJPAR0027446|657[0n]bp|malaise-trapped
Pseudapanteles margaritapenonae|DHJPAR0026998|657[0n]bp|malaise-trapped
Pseudapanteles margaritapenonae|DHJPAR0027455|657[0n]bp|malaise-trapped
Pseudapanteles margaritapenonae|DHJPAR0025952|657[0n]bp|malaise-trapped
Pseudapanteles margaritapenonae|DHJPAR0027450|650[0n]bp|malaise-trapped
Pseudapanteles margaritapenonae|DHJPAR0027076|600[0n]bp|malaise-trapped
Pseudapanteles margaritapenonae|DHJPAR0027108|609[2n]bp|malaise-trapped
Pseudapanteles margaritapenonae|DHJPAR0027410|657[1n]bp|malaise-trapped
Pseudapanteles margaritapenonae|DHJPAR0027407|657[1n]bp|malaise-trapped
Pseudapanteles margaritapenonae|DHJPAR0027331|637[0n]bp|malaise-trapped
Pseudapanteles margaritapenonae|DHJPAR0027452|636[0n]bp|malaise-trapped
Pseudapanteles margaritapenonae|DHJPAR0026973|628[0n]bp|malaise-trapped
Pseudapanteles margaritapenonae|DHJPAR0027369|631[0n]bp|malaise-trapped
Pseudapanteles margaritapenonae|DHJPAR0027330|631[0n]bp|malaise-trapped
Pseudapanteles margaritapenonae|DHJPAR0026107|631[0n]bp|malaise-trapped
Pseudapanteles margaritapenonae|DHJPAR0026033|657[2n]bp|malaise-trapped
Pseudapanteles alfiopivai|DHJPAR0025345|635[0n]bp|malaise-trapped
Pseudapanteles Whitfield27|DHJPAR0043058|658[0n]bp|Eulepte Solis
Pseudapanteles Whitfield27|DHJPAR0043059|658[0n]bp|Eulepte Solis
Pseudapanteles alfiopivai|DHJPAR0012577|657[0n]bp|malaise-trapped
Pseudapanteles Janzen02|DHJPAR0050027|658[0n]bp|same as 12-SRNP-71591
Pseudapanteles Janzen02|DHJPAR0050029|658[0n]bp|same as 12-SRNP-71591
Pseudapanteles Janzen02|DHJPAR0050032|658[0n]bp|same as 12-SRNP-71591
Pseudapanteles Janzen03|DHJPAR0051069|658[0n]bp|same as 12-SRNP-40026
Pseudapanteles carlosespinachi|DHJPAR0039930|468[0n]bp|Desmia Solis100DHJ01
Pseudapanteles Whitfield12|DHJPAR0025507|655[0n]bp|malaise-trapped
Pseudapanteles Whitfield12|DHJPAR0025380|657[0n]bp|malaise-trapped
Pseudapanteles Whitfield12|DHJPAR0025447|645[0n]bp|malaise-trapped
Pseudapanteles carlosespinachi|DHJPAR0039928|555[0n]bp|Desmia Solis100DHJ01
Pseudapanteles Whitfield26|DHJPAR0040493|658[0n]bp|same as 10-2586
Pseudapanteles jorgerodriguezi|DHJPAR0025854|657[3n]bp|malaise-trapped
Pseudapanteles mariobozai|DHJPAR0025932|563[7n]bp|malaise-trapped
Pseudapanteles ruficolis|DHJPAR0052954|658[0n]bp|Desmia ufeus
Pseudapanteles ruficolis|DHJPAR0049218|652[0n]bp|Desmia ufeus
Pseudapanteles laurachinchillae|DHJPAR0026060|624[3n]bp|malaise-trapped
Pseudapanteles ruficolis|DHJPAR0052246|658[0n]bp|

Pseudapanteles ruficolis|DHJPAR0049218|652[0n]bp|Desmia ufeus
Pseudapanteles laurachinchillae|DHJPAR0026060|624[3n]bp|malaise-trapped
Pseudapanteles ruficolis|DHJPAR0052246|658[0n]bp|
Pseudapanteles ruficolis|DHJPAR0047133|658[0n]bp|Desmia see voucher
Pseudapanteles ruficolis|DHJPAR0047117|658[0n]bp|Desmia ufeus
Pseudapanteles carlosespinachi|DHJPAR0004755|606[2n]bp|Desmia Solis100DHJ01
Pseudapanteles ruficolis|DHJPAR0052908|658[0n]bp|Desmia ufeus
Pseudapanteles hernanbravoi|DHJPAR0031742|658[0n]bp|malaise-trapped
Pseudapanteles luisguillermosolisi|DHJPAR0027669|624[0n]bp|malaise-trapped
Pseudapanteles margaritapenonae|DHJPAR0026711|657[2n]bp|malaise-trapped
Pseudapanteles margaritapenonae|DHJPAR0026981|657[0n]bp|malaise-trapped
Pseudapanteles margaritapenonae|DHJPAR0026726|657[1n]bp|malaise-trapped
Pseudapanteles margaritapenonae|DHJPAR0027212|598[2n]bp|malaise-trapped
Pseudapanteles margaritapenonae|DHJPAR0025840|657[1n]bp|malaise-trapped
Pseudapanteles margaritapenonae|DHJPAR0027094|576[0n]bp|malaise-trapped
Pseudapanteles margaritapenonae|DHJPAR0027614|537[21n]bp|malaise-trapped
Pseudapanteles margaritapenonae|DHJPAR0027668|627[9n]bp|malaise-trapped
Pseudapanteles analorenaguevarae|DHJPAR0031191|658[0n]bp|malaise-trapped
Pseudapanteles analorenaguevarae|DHJPAR0034081|658[0n]bp|malaise-trapped
Pseudapanteles analorenaguevarae|DHJPAR0031302|658[0n]bp|malaise-trapped
Pseudapanteles analorenaguevarae|DHJPAR0031187|658[0n]bp|malaise-trapped
Pseudapanteles analorenaguevarae|DHJPAR0031307|658[0n]bp|malaise-trapped
Pseudapanteles analorenaguevarae|DHJPAR0031193|658[0n]bp|malaise-trapped
Pseudapanteles analorenaguevarae|DHJPAR0031329|658[0n]bp|malaise-trapped
Pseudapanteles analorenaguevarae|DHJPAR0031367|658[0n]bp|malaise-trapped
Pseudapanteles analorenaguevarae|DHJPAR0031192|658[0n]bp|malaise-trapped
Pseudapanteles analorenaguevarae|DHJPAR0031398|658[0n]bp|malaise-trapped
Pseudapanteles analorenaguevarae|DHJPAR0031202|658[0n]bp|malaise-trapped
Pseudapanteles analorenaguevarae|DHJPAR0031186|658[0n]bp|malaise-trapped
Pseudapanteles analorenaguevarae|DHJPAR0031332|658[0n]bp|malaise-trapped
Pseudapanteles analorenaguevarae|DHJPAR0031209|658[0n]bp|malaise-trapped
Pseudapanteles analorenaguevarae|DHJPAR0031268|658[0n]bp|malaise-trapped
Pseudapanteles analorenaguevarae|DHJPAR0031347|658[0n]bp|malaise-trapped
Pseudapanteles analorenaguevarae|DHJPAR0031204|658[0n]bp|malaise-trapped
Pseudapanteles analorenaguevarae|DHJPAR0031236|658[0n]bp|malaise-trapped
Pseudapanteles analorenaguevarae|DHJPAR0031321|658[0n]bp|malaise-trapped
Pseudapanteles margaritapenonae|DHJPAR0027088|597[2n]bp|malaise-trapped
Pseudapanteles margaritapenonae|DHJPAR0027176|591[1n]bp|malaise-trapped
Pseudapanteles margaritapenonae|DHJPAR0025860|621[3n]bp|malaise-trapped
Pseudapanteles margaritapenonae|DHJPAR0025866|657[5n]bp|malaise-trapped
Pseudapanteles ottonsolisi|DHJPAR0031749|658[0n]bp|malaise-trapped
Pseudapanteles margaritapenonae|DHJPAR0025826|635[1n]bp|malaise-trapped
Pseudapanteles margaritapenonae|DHJPAR0025858|657[4n]bp|malaise-trapped
Pseudapanteles margaritapenonae|DHJPAR0025827|657[1n]bp|malaise-trapped
Pseudapanteles margaritapenonae|DHJPAR0026275|624[1n]bp|malaise-trapped
Pseudapanteles Janzen02|DHJPAR0052983|658[0n]bp|spiloBioLep01 BioLep414
Pseudapanteles Janzen02|DHJPAR0049258|658[0n]bp|same as 09-SRNP-2456
Pseudapanteles Janzen02|DHJPAR0052317|658[0n]bp|
Pseudapanteles Janzen02|DHJPAR0052275|658[0n]bp|
Pseudapanteles margaritapenonae|DHJPAR0025910|657[0n]bp|malaise-trapped
Pseudapanteles margaritapenonae|DHJPAR0025355|657[0n]bp|malaise-trapped
Pseudapanteles margaritapenonae|DHJPAR0026497|657[0n]bp|malaise-trapped
Pseudapanteles margaritapenonae|DHJPAR0026745|657[0n]bp|malaise-trapped
Pseudapanteles margaritapenonae|DHJPAR0027401|657[0n]bp|malaise-trapped
Pseudapanteles margaritapenonae|DHJPAR0024807|657[0n]bp|malaise-trapped
Pseudapanteles margaritapenonae|DHJPAR0025178|657[0n]bp|malaise-trapped
Pseudapanteles margaritapenonae|DHJPAR0026556|657[0n]bp|malaise-trapped
Pseudapanteles margaritapenonae|DHJPAR0026796|657[0n]bp|malaise-trapped
Pseudapanteles margaritapenonae|DHJPAR0026289|657[0n]bp|malaise-trapped
Pseudapanteles margaritapenonae|DHJPAR0026806|657[0n]bp|malaise-trapped
Pseudapanteles margaritapenonae|DHJPAR0026769|657[0n]bp|malaise-trapped
Pseudapanteles margaritapenonae|DHJPAR0027403|657[0n]bp|malaise-trapped
Pseudapanteles margaritapenonae|DHJPAR0027405|657[0n]bp|malaise-trapped
Pseudapanteles margaritapenonae|DHJPAR0025109|657[0n]bp|malaise-trapped
Pseudapanteles margaritapenonae|DHJPAR0026525|657[0n]bp|malaise-trapped
Pseudapanteles margaritapenonae|DHJPAR0026964|657[0n]bp|malaise-trapped
Pseudapanteles margaritapenonae|DHJPAR0026690|657[0n]bp|malaise-trapped
Pseudapanteles margaritapenonae|DHJPAR0025449|657[0n]bp|malaise-trapped
Pseudapanteles margaritapenonae|DHJPAR0026488|657[0n]bp|malaise-trapped
Pseudapanteles margaritapenonae|DHJPAR0026336|657[0n]bp|malaise-trapped
Pseudapanteles margaritapenonae|DHJPAR0025075|629[0n]bp|malaise-trapped
Pseudapanteles margaritapenonae|DHJPAR0024859|657[0n]bp|malaise-trapped
Pseudapanteles margaritapenonae|DHJPAR0026744|657[0n]bp|malaise-trapped
Pseudapanteles margaritapenonae|DHJPAR0027256|629[0n]bp|malaise-trapped
Pseudapanteles margaritapenonae|DHJPAR0026835|628[0n]bp|malaise-trapped
Pseudapanteles margaritapenonae|DHJPAR0025061|618[0n]bp|malaise-trapped
Pseudapanteles margaritapenonae|DHJPAR0026703|628[0n]bp|malaise-trapped
Pseudapanteles margaritapenonae|DHJPAR0027000|637[0n]bp|malaise-trapped
Pseudapanteles margaritapenonae|DHJPAR0026972|636[0n]bp|malaise-trapped
Pseudapanteles margaritapenonae|DHJPAR0026268|629[0n]bp|malaise-trapped
Pseudapanteles teophilodelatorrei|DHJPAR0048241|658[0n]bp|see description
Pseudapanteles teophilodelatorrei|DHJPAR0048162|658[0n]bp|see description
Pseudapanteles teophilodelatorrei|DHJPAR0045346|658[2n]bp|see description
Pseudapanteles margaritapenonae|DHJPAR0027346|657[0n]bp|malaise-trapped
Pseudapanteles margaritapenonae|DHJPAR0026653|657[0n]bp|malaise-trapped
Pseudapanteles margaritapenonae|DHJPAR0024910|657[0n]bp|malaise-trapped
Pseudapanteles margaritapenonae|DHJPAR0025083|657[0n]bp|malaise-trapped
Pseudapanteles margaritapenonae|DHJPAR0027129|651[0n]bp|malaise-trapped
Pseudapanteles margaritapenonae|DHJPAR0024842|657[0n]bp|malaise-trapped
Pseudapanteles margaritapenonae|DHJPAR0026485|657[0n]bp|malaise-trapped
Pseudapanteles margaritapenonae|DHJPAR0026623|657[0n]bp|malaise-trapped
Pseudapanteles margaritapenonae|DHJPAR0026526|657[0n]bp|malaise-trapped
Pseudapanteles margaritapenonae|DHJPAR0027376|642[0n]bp|malaise-trapped
Pseudapanteles margaritapenonae|DHJPAR0025101|640[0n]bp|malaise-trapped
Pseudapanteles margaritapenonae|DHJPAR0026798|603[0n]bp|malaise-trapped
Pseudapanteles margaritapenonae|DHJPAR0026884|613[3n]bp|malaise-trapped
Pseudapanteles margaritapenonae|DHJPAR0025959|657[3n]bp|malaise-trapped
Pseudapanteles alvaroumanai|DHJPAR0033744|658[0n]bp|malaise-trapped

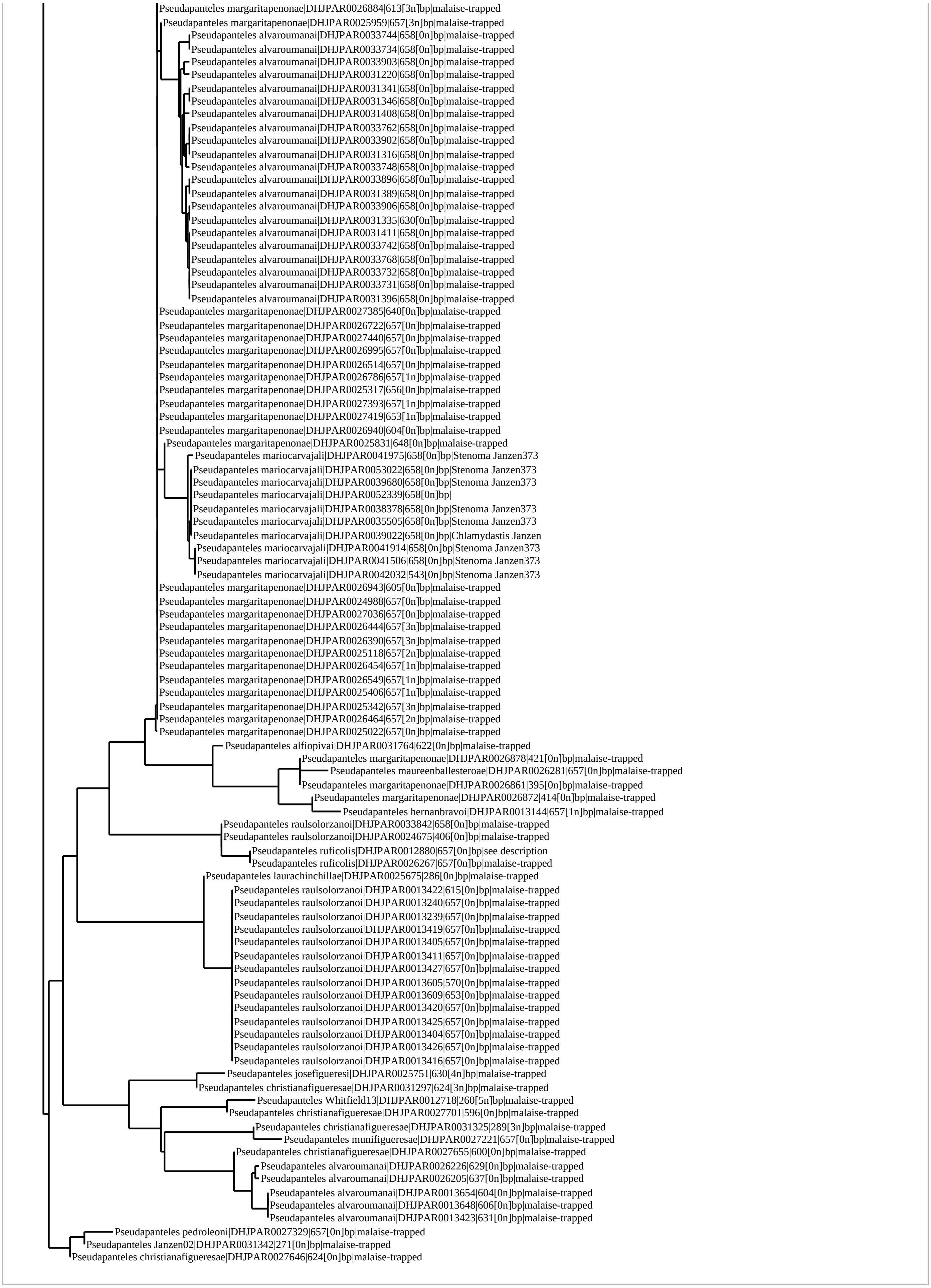
Pseudapanteles margaritapenonae|DHJPAR0026884|613[3n]bp|malaise-trapped
Pseudapanteles margaritapenonae|DHJPAR0025959|657[3n]bp|malaise-trapped
Pseudapanteles alvaroumanai|DHJPAR0033744|658[0n]bp|malaise-trapped
Pseudapanteles alvaroumanai|DHJPAR0033734|658[0n]bp|malaise-trapped
Pseudapanteles alvaroumanai|DHJPAR0033903|658[0n]bp|malaise-trapped
Pseudapanteles alvaroumanai|DHJPAR0031220|658[0n]bp|malaise-trapped
Pseudapanteles alvaroumanai|DHJPAR0031341|658[0n]bp|malaise-trapped
Pseudapanteles alvaroumanai|DHJPAR0031346|658[0n]bp|malaise-trapped
Pseudapanteles alvaroumanai|DHJPAR0031408|658[0n]bp|malaise-trapped
Pseudapanteles alvaroumanai|DHJPAR0033762|658[0n]bp|malaise-trapped
Pseudapanteles alvaroumanai|DHJPAR0033902|658[0n]bp|malaise-trapped
Pseudapanteles alvaroumanai|DHJPAR0031316|658[0n]bp|malaise-trapped
Pseudapanteles alvaroumanai|DHJPAR0033748|658[0n]bp|malaise-trapped
Pseudapanteles alvaroumanai|DHJPAR0033896|658[0n]bp|malaise-trapped
Pseudapanteles alvaroumanai|DHJPAR0031389|658[0n]bp|malaise-trapped
Pseudapanteles alvaroumanai|DHJPAR0033906|658[0n]bp|malaise-trapped
Pseudapanteles alvaroumanai|DHJPAR0031335|630[0n]bp|malaise-trapped
Pseudapanteles alvaroumanai|DHJPAR0031411|658[0n]bp|malaise-trapped
Pseudapanteles alvaroumanai|DHJPAR0033742|658[0n]bp|malaise-trapped
Pseudapanteles alvaroumanai|DHJPAR0033768|658[0n]bp|malaise-trapped
Pseudapanteles alvaroumanai|DHJPAR0033732|658[0n]bp|malaise-trapped
Pseudapanteles alvaroumanai|DHJPAR0033731|658[0n]bp|malaise-trapped
Pseudapanteles alvaroumanai|DHJPAR0031396|658[0n]bp|malaise-trapped
Pseudapanteles margaritapenonae|DHJPAR0027385|640[0n]bp|malaise-trapped
Pseudapanteles margaritapenonae|DHJPAR0026722|657[0n]bp|malaise-trapped
Pseudapanteles margaritapenonae|DHJPAR0027440|657[0n]bp|malaise-trapped
Pseudapanteles margaritapenonae|DHJPAR0026995|657[0n]bp|malaise-trapped
Pseudapanteles margaritapenonae|DHJPAR0026514|657[0n]bp|malaise-trapped
Pseudapanteles margaritapenonae|DHJPAR0026786|657[1n]bp|malaise-trapped
Pseudapanteles margaritapenonae|DHJPAR0025317|656[0n]bp|malaise-trapped
Pseudapanteles margaritapenonae|DHJPAR0027393|657[1n]bp|malaise-trapped
Pseudapanteles margaritapenonae|DHJPAR0027419|653[1n]bp|malaise-trapped
Pseudapanteles margaritapenonae|DHJPAR0026940|604[0n]bp|malaise-trapped
Pseudapanteles margaritapenonae|DHJPAR0025831|648[0n]bp|malaise-trapped
Pseudapanteles mariocarvajali|DHJPAR0041975|658[0n]bp|Stenoma Janzen373
Pseudapanteles mariocarvajali|DHJPAR0053022|658[0n]bp|Stenoma Janzen373
Pseudapanteles mariocarvajali|DHJPAR0039680|658[0n]bp|Stenoma Janzen373
Pseudapanteles mariocarvajali|DHJPAR0052339|658[0n]bp|
Pseudapanteles mariocarvajali|DHJPAR0038378|658[0n]bp|Stenoma Janzen373
Pseudapanteles mariocarvajali|DHJPAR0035505|658[0n]bp|Stenoma Janzen373
Pseudapanteles mariocarvajali|DHJPAR0039022|658[0n]bp|Chlamydastis Janzen
Pseudapanteles mariocarvajali|DHJPAR0041914|658[0n]bp|Stenoma Janzen373
Pseudapanteles mariocarvajali|DHJPAR0041506|658[0n]bp|Stenoma Janzen373
Pseudapanteles mariocarvajali|DHJPAR0042032|543[0n]bp|Stenoma Janzen373
Pseudapanteles margaritapenonae|DHJPAR0026943|605[0n]bp|malaise-trapped
Pseudapanteles margaritapenonae|DHJPAR0024988|657[0n]bp|malaise-trapped
Pseudapanteles margaritapenonae|DHJPAR0027036|657[0n]bp|malaise-trapped
Pseudapanteles margaritapenonae|DHJPAR0026444|657[3n]bp|malaise-trapped
Pseudapanteles margaritapenonae|DHJPAR0026390|657[3n]bp|malaise-trapped
Pseudapanteles margaritapenonae|DHJPAR0025118|657[2n]bp|malaise-trapped
Pseudapanteles margaritapenonae|DHJPAR0026454|657[1n]bp|malaise-trapped
Pseudapanteles margaritapenonae|DHJPAR0026549|657[1n]bp|malaise-trapped
Pseudapanteles margaritapenonae|DHJPAR0025406|657[1n]bp|malaise-trapped
Pseudapanteles margaritapenonae|DHJPAR0025342|657[3n]bp|malaise-trapped
Pseudapanteles margaritapenonae|DHJPAR0026464|657[2n]bp|malaise-trapped
Pseudapanteles margaritapenonae|DHJPAR0025022|657[0n]bp|malaise-trapped
Pseudapanteles alfiopivai|DHJPAR0031764|622[0n]bp|malaise-trapped
Pseudapanteles margaritapenonae|DHJPAR0026878|421[0n]bp|malaise-trapped
Pseudapanteles maureenballesteroae|DHJPAR0026281|657[0n]bp|malaise-trapped
Pseudapanteles margaritapenonae|DHJPAR0026861|395[0n]bp|malaise-trapped
Pseudapanteles margaritapenonae|DHJPAR0026872|414[0n]bp|malaise-trapped
Pseudapanteles hernanbravoi|DHJPAR0013144|657[1n]bp|malaise-trapped
Pseudapanteles raulsolorzanoi|DHJPAR0033842|658[0n]bp|malaise-trapped
Pseudapanteles raulsolorzanoi|DHJPAR0024675|406[0n]bp|malaise-trapped
Pseudapanteles ruficolis|DHJPAR0012880|657[0n]bp|see description
Pseudapanteles ruficolis|DHJPAR0026267|657[0n]bp|malaise-trapped
Pseudapanteles laurachinchillae|DHJPAR0025675|286[0n]bp|malaise-trapped
Pseudapanteles raulsolorzanoi|DHJPAR0013422|615[0n]bp|malaise-trapped
Pseudapanteles raulsolorzanoi|DHJPAR0013240|657[0n]bp|malaise-trapped
Pseudapanteles raulsolorzanoi|DHJPAR0013239|657[0n]bp|malaise-trapped
Pseudapanteles raulsolorzanoi|DHJPAR0013419|657[0n]bp|malaise-trapped
Pseudapanteles raulsolorzanoi|DHJPAR0013405|657[0n]bp|malaise-trapped
Pseudapanteles raulsolorzanoi|DHJPAR0013411|657[0n]bp|malaise-trapped
Pseudapanteles raulsolorzanoi|DHJPAR0013427|657[0n]bp|malaise-trapped
Pseudapanteles raulsolorzanoi|DHJPAR0013605|570[0n]bp|malaise-trapped
Pseudapanteles raulsolorzanoi|DHJPAR0013609|653[0n]bp|malaise-trapped
Pseudapanteles raulsolorzanoi|DHJPAR0013420|657[0n]bp|malaise-trapped
Pseudapanteles raulsolorzanoi|DHJPAR0013425|657[0n]bp|malaise-trapped
Pseudapanteles raulsolorzanoi|DHJPAR0013404|657[0n]bp|malaise-trapped
Pseudapanteles raulsolorzanoi|DHJPAR0013426|657[0n]bp|malaise-trapped
Pseudapanteles raulsolorzanoi|DHJPAR0013416|657[0n]bp|malaise-trapped
Pseudapanteles josefigueresi|DHJPAR0025751|630[4n]bp|malaise-trapped
Pseudapanteles christianafigueresae|DHJPAR0031297|624[3n]bp|malaise-trapped
Pseudapanteles Whitfield13|DHJPAR0012718|260[5n]bp|malaise-trapped
Pseudapanteles christianafigueresae|DHJPAR0027701|596[0n]bp|malaise-trapped
Pseudapanteles christianafigueresae|DHJPAR0031325|289[3n]bp|malaise-trapped
Pseudapanteles munifigueresae|DHJPAR0027221|657[0n]bp|malaise-trapped
Pseudapanteles christianafigueresae|DHJPAR0027655|600[0n]bp|malaise-trapped
Pseudapanteles alvaroumanai|DHJPAR0026226|629[0n]bp|malaise-trapped
Pseudapanteles alvaroumanai|DHJPAR0026205|637[0n]bp|malaise-trapped
Pseudapanteles alvaroumanai|DHJPAR0013654|604[0n]bp|malaise-trapped
Pseudapanteles alvaroumanai|DHJPAR0013648|606[0n]bp|malaise-trapped
Pseudapanteles alvaroumanai|DHJPAR0013423|631[0n]bp|malaise-trapped
Pseudapanteles pedroleoni|DHJPAR0027329|657[0n]bp|malaise-trapped
Pseudapanteles Janzen02|DHJPAR0031342|271[0n]bp|malaise-trapped
Pseudapanteles christianafigueresae|DHJPAR0027646|624[0n]bp|malaise-trapped
